# Systemic pharmacological interventions for Ménière’s disease

**DOI:** 10.1002/14651858.CD015171.pub2

**Published:** 2023-02-23

**Authors:** Katie E Webster, Kevin Galbraith, Natasha A Harrington-Benton, Owen Judd, Diego Kaski, Otto R Maarsingh, Samuel MacKeith, Jaydip Ray, Vincent A Van Vugt, Martin J Burton

**Affiliations:** Cochrane ENTNuffield Department of Surgical Sciences, University of OxfordOxfordUK; Ménière’s SocietyWootonUK; ENT DepartmentUniversity Hospitals of Derby and Burton NHS Foundation TrustDerbyUK; National Hospital for Neurology and NeurosurgeryLondonUK; Department of General Practice, Amsterdam UMCVrije Universiteit Amsterdam, Amsterdam Public Health Research InstituteAmsterdamNetherlands; ENT DepartmentOxford University Hospitals NHS Foundation TrustOxfordUK; University of SheffieldSheffieldUK; Cochrane UKOxfordUK

**Keywords:** Adult, Humans, Adrenal Cortex Hormones, Betahistine, Diuretics, Histamine Antagonists, Meniere Disease, Meniere Disease/therapy, Tinnitus, Vertigo

## Abstract

**Background:**

Ménière's disease is a condition that causes recurrent episodes of vertigo, associated with hearing loss and tinnitus. A number of pharmacological interventions have been used in the management of this condition, including betahistine, diuretics, antiviral medications and corticosteroids. The underlying cause of Ménière's disease is unknown, as is the way in which these treatments may work. The efficacy of these different interventions at preventing vertigo attacks, and their associated symptoms, is currently unclear.

**Objectives:**

To evaluate the benefits and harms of systemic pharmacological interventions versus placebo or no treatment in people with Ménière's disease.

**Search methods:**

The Cochrane ENT Information Specialist searched the Cochrane ENT Register; Central Register of Controlled Trials (CENTRAL); Ovid MEDLINE; Ovid Embase; Web of Science; ClinicalTrials.gov; ICTRP and additional sources for published and unpublished trials. The date of the search was 14 September 2022.

**Selection criteria:**

We included randomised controlled trials (RCTs) and quasi‐RCTs in adults with definite or probable Ménière's disease comparing betahistine, diuretics, antihistamines, antivirals or systemic corticosteroids with either placebo or no treatment. We excluded studies with follow‐up of less than three months, or with a cross‐over design (unless data from the first phase of the study could be identified).

**Data collection and analysis:**

We used standard Cochrane methods. Our primary outcomes were: 1) improvement in vertigo (assessed as a dichotomous outcome ‐ improved or not improved), 2) change in vertigo (assessed as a continuous outcome, with a score on a numerical scale) and 3) serious adverse events. Our secondary outcomes were: 4) disease‐specific health‐related quality of life, 5) change in hearing, 6) change in tinnitus and 7) other adverse effects. We considered outcomes reported at three time points: 3 to < 6 months, 6 to ≤ 12 months and > 12 months. We used GRADE to assess the certainty of evidence for each outcome.

**Main results:**

We included 10 studies with a total of 848 participants. The studies evaluated the following interventions: betahistine, diuretics, antivirals and corticosteroids. We did not identify any evidence on antihistamines.

**Betahistine**

Seven RCTs (548 participants) addressed this comparison. However, we were unable to conduct any meta‐analyses for our primary outcomes as not all outcomes were considered by every study, and studies that did report the same outcome used different time points for follow‐up, or assessed the outcome using different methods. Therefore, we were unable to draw meaningful conclusions from the numerical results. Some data were available for each of our primary outcomes, but the evidence was low‐ or very low‐certainty throughout. One study reported on the outcome 'improvement in vertigo' at 6 to ≤ 12 months, and another study reported this outcome at > 12 months. Four studies reported on the change in vertigo, but again all used different methods of assessment (vertigo frequency, or a global score of vertigo severity) or different time points. A single study reported on serious adverse events.

**Diuretics**

Two RCTs addressed this comparison. One considered the use of isosorbide (220 participants), and the other used a combination of amiloride hydrochloride and hydrochlorothiazide (80 participants). Again, we were unable to conduct any meta‐analyses for our primary outcomes, as only one study reported on the outcome 'improvement in vertigo' (at 6 to ≤ 12 months), one study reported on change in vertigo (at 3 to < 6 months) and neither study assessed serious adverse events. Therefore, we were unable to draw meaningful conclusions from the numerical results. The evidence was all very low‐certainty.

**Other pharmacological interventions**

We also identified one study that assessed antivirals (24 participants), and one study that assessed corticosteroids (16 participants). The evidence for these interventions was all very low‐certainty. Again, serious adverse events were not considered by either study.

**Authors' conclusions:**

The evidence for systemic pharmacological interventions for Ménière's disease is very uncertain. There are few RCTs that compare these interventions to placebo or no treatment, and the evidence that is currently available from these studies is of low or very low certainty. This means that we have very low confidence that the effects reported are accurate estimates of the true effect of these interventions. Consensus on the appropriate outcomes to measure in studies of Ménière's disease is needed (i.e. a core outcome set) in order to guide future studies in this area and enable meta‐analyses of the results. This must include appropriate consideration of the potential harms of treatment, as well as the benefits.

## Plain language summary

**What are the benefits and risks of different medications for Ménière's disease?**

**Key messages**

Due to a lack of robust evidence, it is not clear whether any oral medicines (tablets) used to treat Ménière's disease work to improve people's symptoms, despite their routine use in clinical practice. When used for other conditions, these medications may cause side effects. However, there was too little information from the studies in Ménière’s disease for us to know if there are serious risks from these treatments.

Larger, well‐conducted studies are needed in order to identify whether some medications may be effective, and assess whether there are any harmful effects of treatment.

Further work also needs to be done to find out how best to measure the symptoms of people with Ménière's disease, in order to assess whether treatments are beneficial or not. This should include the development of a 'core outcome set' ‐ a list of things that should be measured in all studies on Ménière's disease.

**What is Ménière's disease?**

Ménière's disease is a condition that affects the inner ear. It causes repeated attacks of dizziness or vertigo (a spinning sensation), together with hearing problems, tinnitus (ringing, humming or buzzing noises in the ears) and a feeling of fullness or pressure in the ear. It usually affects adults, and starts in middle age.

**How is Ménière's disease treated?**

Oral medications (tablets) are often used as the first treatment for Ménière's disease. Medications like betahistine and diuretics are commonly used, but other treatments have also been used, including corticosteroids and antiviral treatments. Other treatment options are also available (for example, injections into the ear or surgery).

**What did we want to find out?**

We wanted to find out:

‐ whether there was evidence that any oral medications work at reducing the symptoms of Ménière's disease;

‐ whether the treatments might cause any harm.

**What did we do?**

We searched for studies that compared different types of treatment to either no treatment or sham (placebo) treatment.

**What did we find?**

We found 10 studies, which included a total of 848 people. They lasted between three months and two years. Most of the studies looked at treatment with betahistine, two studies looked at diuretics, one looked at corticosteroids and one looked at antivirals.

*Betahistine*

It is unclear whether betahistine has an effect on vertigo symptoms. It is also unclear whether it causes any harm. Although we found several studies, many did not report on the things we were interested in, or reported them at very different times, so we were not able to combine the results of different studies to get a more accurate answer.

*Diuretics*

It is also unclear whether diuretics have an effect on vertigo symptoms. We found no studies that reported on possible harms from this treatment.

*Antivirals and corticosteroids*

For each we only found one small study that looked at these treatments, so we are very uncertain whether they have any effect on vertigo.

**What are the limitations of the evidence?**

We have very little confidence in the evidence because most of the studies conducted were very small and had problems in their conduct, which means that the results may be unreliable. This may be surprising, as some of these treatments are widely used for Ménière's disease. However, larger, well‐conducted studies are needed to try and work out how effective the different treatments really are.

**How up‐to‐date is this evidence?**

This evidence is up‐to‐date to September 2022.

## Summary of findings

**Summary of findings 1:** Betahistine compared to placebo/no treatment for Ménière’s disease

**Betahistine compared to placebo/no treatment for Ménière’s disease**						
**Patient or population:** Ménière’s disease **Setting: **outpatients **Intervention:** betahistine (total daily dose ranging from 24 mg to 144 mg) **Comparison:** placebo/no treatment						
**Outcomes**	Anticipated absolute effects^*^ (95% CI)		**Relative effect (95% CI)**	**№ of participants (studies)**	**Certainty of the evidence (GRADE)**	**Comments**
	**Risk with placebo/no treatment**	**Risk with betahistine**				
Improvement in vertigo frequency Assessed with: self‐rated improvement in either frequency or severity of vertigo Follow‐up: range 6 months to ≤ 12 months	Study population		RR 1.50 (0.98 to 2.29)	70 (1 RCT)	⊕⊝⊝⊝ **very low**^1,2,3,4,5^	The evidence is very uncertain about the effect of betahistine on improvement in vertigo frequency at 6 to ≤ 12 months.
	457 participants per 1000 would report that their vertigo had improved	686 participants per 1000 would report that their vertigo had improved (from 448 to 1000)				
Improvement in vertigo frequency Assessed with: AAO‐HNS 1995 class A, B or C Follow‐up: range > 12 months	Study population		RR 1.11 (0.93 to 1.32)	62 (1 RCT)	⊕⊝⊝⊝ **very low**^4,5,6,7^	The evidence is very uncertain about the effect of betahistine on improvement in vertigo frequency at > 12 months.
	844 participants per 1000 would report that their vertigo had improved	937 participants per 1000 would report that their vertigo had improved (from 785 to 1000)				
Vertigo global score Assessed with: geometric mean of monthly imbalance score (range 0 to 63, higher scores = worse symptoms) Follow‐up: range 3 months to < 6 months	The mean vertigo global score was 6.2 points	MD 0.7 points higher (6.67 lower to 8.07 higher)	—	34 (1 RCT)	⊕⊝⊝⊝ **very low**^4,8,9^	The evidence is very uncertain about the effect of betahistine on change in vertigo (using a global score) at 3 to < 6 months.
Change in vertigo frequency Assessed with: number of attacks per month Follow‐up: range 3 months to < 6 months	The mean vertigo frequency was 4.68 attacks per month	MD 1.90 attacks per month lower (3.05 lower to 0.74 lower)	—	117 (2 RCTs)	⊕⊝⊝⊝ **very low**^4,5,10,11^	The evidence is very uncertain about the effect of betahistine on change in vertigo (using the frequency of attacks) at 3 to < 6 months.
Change in vertigo frequency Assessed with: average number of attacks in 30 days Follow‐up: range 6 months to ≤ 12 months	The mean vertigo frequency was 3.084 attacks per 30 days	MD 0.63 attacks per 30 days higher (4.07 lower to 5.33 higher)	—	214 (1 RCT)	⊕⊝⊝⊝ **very low**^4,9,12^	The evidence is very uncertain about the effect of betahistine on change in vertigo (using the frequency of attacks) at 6 to ≤ 12 months.
Serious adverse events	Study population		RR 1.20 (0.63 to 2.29)	220 (1 RCT)	⊕⊝⊝⊝ **very low**^4,9,12^	The evidence is very uncertain about the effect of betahistine on serious adverse events.
	149 per 1000	178 per 1000 (94 to 340)				
*The risk in the intervention group (and its 95% confidence interval) is based on the assumed risk in the comparison group and the **relative effect** of the intervention (and its 95% CI). **CI:** confidence interval; **MD:** mean difference; **RCT:** randomised controlled trial; **RR:** risk ratio						
**GRADE Working Group grades of evidence** **High certainty:** we are very confident that the true effect lies close to that of the estimate of the effect. **Moderate certainty:** we are moderately confident in the effect estimate: the true effect is likely to be close to the estimate of the effect, but there is a possibility that it is substantially different. **Low certainty:** our confidence in the effect estimate is limited: the true effect may be substantially different from the estimate of the effect. **Very low certainty:** we have very little confidence in the effect estimate: the true effect is likely to be substantially different from the estimate of effect.						

^1^High risk of bias for 5 domains in this study, and unclear risk of bias for remaining 2 domains. ^2^The criteria used for the diagnosis of Ménière's disease were poorly defined, therefore the population may not be appropriate. ^3^This outcome was reported as an improvement in either frequency or severity of attacks, not only frequency. ^4^Optimal information size was not reached (taken as < 300 events for dichotomous outcomes or < 400 participants for continuous outcomes, as a rule of thumb). ^5^Confidence interval ranges from a likely trivial effect to potential benefit. ^6^Unclear risk of bias for several domains, and high risk of bias due to differential use of intratympanic steroids in the intervention and control group. ^7^All participants also received intratympanic dexamethasone injections throughout the trial. ^8^Multiple domains at unclear risk of bias leading to an overall concern about the risk for this trial. ^9^Confidence interval ranges from potential harm to potential benefit. ^10^Multiple bias domains rated at unclear risk of bias. High risk of selective reporting bias due to incomplete outcome data for this result. ^11^Numeric data used in this analysis were estimated due to incomplete reporting in the article. ^12^High risk of attrition bias, and potential for selective reporting.

**Summary of findings 2:** Diuretic compared to placebo/no treatment for Ménière’s disease

**Diuretic compared to placebo/no treatment for Ménière’s disease**						
**Patient or population:** Ménière’s disease **Setting:** outpatients **Intervention:** diuretic (isosorbide or amiloride/hydrochlorothiazide combination) **Comparison:** placebo/no treatment						
**Outcomes**	Anticipated absolute effects^*^ (95% CI)		**Relative effect (95% CI)**	**№ of participants (studies)**	**Certainty of the evidence (GRADE)**	**Comments**
	**Risk with placebo/no treatment**	**Risk with diuretic**				
Improvement in vertigo frequency Assessed with: self‐rated improvement in either frequency or severity of vertigo Follow‐up: range 6 months to ≤ 12 months	Study population		RR 1.69 (1.13 to 2.53)	70 (1 RCT)	⊕⊝⊝⊝ **very low**^1,2,3,4^	The evidence is very uncertain about the effect of diuretics on improvement in vertigo frequency at 6 to ≤ 12 months.
	457 participants per 1000 would report that their vertigo had improved	773 participants per 1000 would report that their vertigo had improved (from 517 to 1000)				
Change in vertigo frequency Assessed with: number of episodes during a 4 week‐period Follow‐up: range 3 months to ≤ 6 months	The mean change in vertigo frequency was ‐1.4 episodes per 4 weeks	MD 2.44 episodes per 4 weeks lower (4.98 lower to 0.1 higher)	‐	220 (1 RCT)	⊕⊝⊝⊝ **very low**^4,5,6,7^	The evidence is very uncertain about the effect of diuretics on the change in vertigo frequency at 6 to ≤ 12 months.
*The risk in the intervention group (and its 95% confidence interval) is based on the assumed risk in the comparison group and the **relative effect** of the intervention (and its 95% CI). **CI:** confidence interval; **MD:** mean difference; **RCT:** randomised controlled trial; **RR:** risk ratio						
**GRADE Working Group grades of evidence** **High certainty:** we are very confident that the true effect lies close to that of the estimate of the effect. **Moderate certainty:** we are moderately confident in the effect estimate: the true effect is likely to be close to the estimate of the effect, but there is a possibility that it is substantially different. **Low certainty:** our confidence in the effect estimate is limited: the true effect may be substantially different from the estimate of the effect. **Very low certainty:** we have very little confidence in the effect estimate: the true effect is likely to be substantially different from the estimate of effect.						

^1^High risk of bias for five domains in this study and unclear risk of bias for the remaining two domains. ^2^The criteria used for the diagnosis of Ménière's disease were poorly defined, therefore the population may not be appropriate. ^3^This outcome was reported as an improvement in either frequency or severity of attacks, not only frequency. ^4^Optimal information size was not reached (taken as < 300 events for dichotomous outcomes or < 400 participants for continuous outcomes, as a rule of thumb). ^5^High risk of performance and detection bias. Unclear risk of bias for multiple domains. ^6^All participants were also taking betahistine for the duration of the trial. ^7^Confidence interval ranges from a trivial effect to potential benefit.

## Background

### Description of the condition

Ménière's disease was first described by Prosper Ménière in 1861 as a condition characterised by episodes of vertigo, associated with hearing loss and tinnitus (Baloh 2001). Sufferers may also report a feeling of fullness in the affected ear. Typically, it initially affects one ear, although some individuals may progress to develop bilateral disease. A hallmark of the condition is that symptoms are intermittent ‐ occurring as discrete attacks that last from minutes to several hours, then resolve. However, over time there is usually a gradual deterioration in hearing, and there may be progressive loss of balance function, leading to chronic dizziness.

The diagnosis of Ménière's disease is challenging, due to the episodic nature of the condition, clinical heterogeneity and the lack of a 'gold standard' diagnostic test. Even the agreed, international classification system has scope for two categories of diagnosis – 'definite' and 'probable' (Lopez‐Escamez 2015). In brief, a diagnosis of definite Ménière's disease requires at least two episodes of vertigo, each lasting 20 minutes to 12 hours, together with audiometrically confirmed hearing loss and fluctuating aural symptoms (reduction in hearing, tinnitus or fullness) in the affected ear. 'Probable' Ménière's disease includes similar features, but without the requirement for audiometry to diagnose hearing loss, and with scope for the vertigo episodes to last longer (up to 24 hours). Both categories ('definite' and 'probable') require that the symptoms are not more likely to be due to an alternative diagnosis, due to the recognised challenges in distinguishing between balance disorders. 

Given the difficulties in diagnosis, the true incidence and prevalence of the disease are difficult to ascertain. A population‐based study in the UK using general practice data estimated the incidence to be 13.1 per 100,000 person‐years (Bruderer 2017), and the prevalence of the disease has been estimated at 190 per 100,000 people in the US (Harris 2010). It is a disorder of midlife, with diagnosis typically occurring between the ages of 30 and 60 (Harcourt 2014). Some studies report a slight female preponderance, and there may be a familial association, with approximately 10% of patients reporting the presence of the disease in a first, second or third degree relative (Requena 2014).

The underlying cause of Ménière's disease is usually not known. Ménière's disease has been associated with an increase in the volume of fluid in the inner ear (endolymphatic hydrops). This may be caused by the abnormal production or resorption of endolymph (Hallpike 1938; Yamakawa 1938). However, it is not clear whether this is the underlying cause of the condition, or merely associated with the disease. Some authors have proposed other underlying causes for Ménière's disease, including viral infections (Gacek 2009), and allergic (Banks 2012) or autoimmune disease processes (Greco 2012). A genetic predisposition has also been noted (Chiarella 2015). Occasionally, the symptoms may be secondary to a known cause (such as a head injury or other inner ear disorder) – in these cases it may be referred to as Ménière's syndrome.

Although Ménière's disease is relatively uncommon, it has a profound impact on quality of life. The unpredictable, episodic nature of the condition and severe, disabling attacks of vertigo cause a huge amount of distress. Quality of life (including physical and psychosocial aspects) is significantly reduced for those with Ménière's disease (Söderman 2002). The costs of the condition are also considerable, both in relation to medical interventions (appointments, diagnostic tests and treatments) and loss of productivity or sick days for those affected by the condition (Tyrrell 2016).

### Description of the intervention

A variety of different interventions have been proposed to treat people with Ménière's disease. These include dietary or lifestyle changes, oral treatments, treatments administered by injection into the ear (intratympanic) and surgical treatments. This review focuses on the use of medications that are given systemically (typically orally) to treat the symptoms of Ménière's disease. A survey of consultant otolaryngologists in the UK identified that 66% of them always prescribed medication for individuals with Ménière's disease, and a further 30% sometimes prescribed medication (Smith 2005).

Two of the most common treatments for Ménière's disease are betahistine and diuretics. Both of these treatments are taken regularly. Different doses of betahistine may be used for Ménière's disease, and people may take their tablets either two or three times a day. Diuretics include many classes of drugs – those commonly used for Ménière's disease are thiazides, but others that may be used include potassium sparing diuretics, carbonic anhydrase inhibitors and loop diuretics. A UK‐based survey found betahistine to be the most commonly prescribed medication (used by 85% of ENT surgeons; Smith 2005). A similar pattern was seen in a survey of Italian ENT surgeons, where 78.4% used betahistine as maintenance treatment for the disease, compared to 52.8% who used diuretics (Quaranta 2019). However, betahistine remains unlicensed by the Food and Drug Administration, so its use is likely to be much lower in the USA. 

Less frequently, other oral treatments may be used. For example, antiviral medicines, antihistamines (other than betahistine) or oral steroids.

At present, there is no agreement on which is the ideal treatment for people with Ménière's disease – consequently there is no 'gold standard' treatment with which to compare these medications. 

### How the intervention might work

As the underlying cause of Ménière's disease is poorly understood, so too are the ways in which the interventions may work.

Several classes of histamine receptor are found within the inner ear. Betahistine is a histamine H₃ antagonist, and a weak H₁ agonist (Arrang 1985). Betahistine is thought to increase the blood flow in the inner ear ‐ this may impact upon endolymphatic fluid pressure. It may also have a direct effect on the vestibular nerve to reduce nerve cell firing and the frequency of vertigo attacks (Botta 1998; Chávez 2005). Other antihistamines may also be used, such as cinnarizine or dimenhydrinate. 

Diuretics are used with the intention that they will reduce the volume of endolymph and the pressure in the endolymphatic system, by altering the electrolyte balance and promoting water loss through the kidneys. The mechanism of action varies depending on the class of drug (reviewed in Odlind 1984) and includes:

- inhibition of renal carbonic anhydrase in the proximal tubules, resulting in increased bicarbonate and sodium excretion (carbonic anhydrase inhibitors);
- inhibition of chloride transport in the ascending loop (loop diuretics);
- inhibition of sodium and calcium resorption in the distal tubules (thiazides); and
- alteration of electrolyte transport in the distal tubules and collecting ducts (potassium sparing diuretics).

As noted above, it has been suggested that some cases of Ménière's disease may be caused by a viral infection. Consequently, there has been interest in the use of antiviral medication, such as aciclovir, to try and treat any underlying viral trigger.

The possibility of an allergic or autoimmune cause for the condition, together with presumed inflammation of the audiovestibular structures in the inner ear (Frejo 2017) has also led to trials of systemic steroids as a treatment for the disease, as these drugs are widely used for their anti‐inflammatory and immunomodulatory effects.

### Why it is important to do this review

Balance disorders can be difficult to diagnose and treat. There are few specific diagnostic tests, a variety of related disorders with similar symptoms and a limited number of interventions that are known to be effective. To determine which topics within this area should be addressed with new or updated systematic reviews we conducted a scoping and prioritisation process, involving stakeholders (https://ent.cochrane.org/balance-disorders-ent). Ménière's disease was ranked as one of the highest priority topics during this process (along with vestibular migraine and persistent postural perceptual dizziness). 

Although Ménière's disease is a relatively uncommon condition, the significant impact it has on quality of life demonstrates the clear importance of identifying effective interventions to alleviate the symptoms. There is considerable variation in the management of Ménière's disease on both a national and international scale, with a lack of consensus about appropriate first‐line and subsequent therapies. 

This review is part of a suite of six that consider different interventions for Ménière's disease. Through these reviews, we hope to provide a thorough summary of the efficacy (benefits and harms) of the different treatment options, to support people with Ménière's disease (and healthcare professionals) when making decisions about their care. 

## Objectives

To evaluate the benefits and harms of systemic pharmacological interventions versus placebo or no treatment in people with Ménière's disease.

## Methods

### Criteria for considering studies for this review

#### Types of studies

We included randomised controlled trials (RCTs) and quasi‐randomised trials (where trials were designed as RCTs, but the sequence generation for allocation of treatment used methods such as alternate allocation, birth dates etc). 

Ménière's disease is known to fluctuate over time, which may mean that cross‐over trials are not an appropriate study design for this condition. Cross‐over RCTs were only included if data could be extracted for the first phase of the study (this applied to a single RCT: Schmidt 1992). No cluster‐RCTs were identified as relevant for inclusion in this review.

We included studies reported as full text, those published as conference abstracts only and unpublished data. 

Ménière's disease is characterised by episodic balance disturbance ‐ the frequency of attacks may change over time (Huppert 2010). For studies to obtain accurate estimates of the effect of different interventions, we considered that follow‐up of participants should be for at least three months ‐ to ensure that participants are likely to have experienced a number of attacks during the follow‐up period. Studies that followed up participants for fewer than three months were excluded from the review.

#### Types of participants

We included studies that recruited adult participants (aged 18 years or older) with a diagnosis of definite or probable Ménière's disease, according to the agreed criteria of the American Academy Otolaryngology ‐ Head and Neck Surgery (AAO‐HNS), the Japan Society for Equilibrium Research, the European Academy of Otology and Neurotology and the Bárány Society. These criteria are outlined in Appendix 1 and described in Lopez‐Escamez 2015. 

If studies used different criteria to diagnose Ménière's disease, we included them if those criteria were clearly analogous to those described in Lopez‐Escamez 2015. For example, studies that used earlier definitions of Ménière's disease (from the AAO‐HNS guidelines of 1995) were also included. If there was uncertainty over the criteria used for the study, then we made a decision on whether to include the study. This decision was taken by authors who were masked to other features of the studies (such as study size, other aspects of methodology, results of the study) to avoid the introduction of bias in study selection. If a study was conducted in an ENT department and participants were diagnosed with Ménière's disease then we considered it was likely that other diagnoses had been excluded, and included the study. However, we reflected this uncertainty in diagnosis by considering the study at risk of indirectness when using GRADE to assess the certainty of the evidence (see 'Summary of findings and assessment of certainty of the evidence'). 

We anticipated that most studies would include participants with active Ménière's disease. We did not exclude studies if the frequency of attacks at baseline was not reported or was unclear, but we planned to highlight if there were differences between studies that may impact on our ability to pool the data, or affect the applicability of our findings.

We excluded studies where participants had previously undergone destructive/ablative treatment for Ménière's disease in the affected ear (such as vestibular neurectomy, chemical or surgical labyrinthectomy), as we considered that they were unlikely to respond to interventions in the same way as those who had not undergone such treatment.

#### Types of interventions

We included the following interventions:

- Betahistine
- Diuretics
- Antihistamines (other than betahistine)
- Antiviral medication
- Corticosteroids

Studies using any systemic route of administration were included (oral, parenteral). Intratympanic administration of corticosteroids is assessed as part of a separate review (Webster 2021a), therefore is not included here. As betahistine has histamine antagonist and agonist effects, it was considered separately to other antihistamines.  

The main comparisons are the following:

- Betahistine versus placebo/no treatment
- Diuretics versus placebo/no treatment
- Antihistamines versus placebo/no treatment
- Antivirals versus placebo/no treatment
- Steroids versus placebo/no treatment

##### Concurrent treatments

There were no limits on the type of concurrent treatments used, providing these were used equally in each arm of the study. We pooled studies that included concurrent treatments with those where participants did not receive concurrent treatment. We planned to conduct subgroup analysis to determine whether the effect estimates may be different in those receiving additional treatment. However, due to the small number of studies included in the review this was not possible (see Subgroup analysis and investigation of heterogeneity).  

#### Types of outcome measures

We assessed all outcomes at the following time points: 

- 3 to < 6 months
- 6 to ≤ 12 months
- > 12 months

The exception was for adverse event data, when we used the longest time period of follow‐up. 

We searched the COMET database for existing core outcome sets of relevance to Ménière's disease and vertigo, but were unable to find any published core outcome sets. We therefore conducted a survey of individuals with experience of (or an interest in) balance disorders to help identify the outcomes that should be prioritised. This online survey was conducted with the support of the Ménière's Society and the Migraine Trust, and included 324 participants who provided information regarding priority outcomes. The review author team used the results of this survey to inform the choice of outcome measures in this review. 

We analysed the following outcomes in the review, but did not use them as a basis for including or excluding studies.

##### Primary outcomes

- Improvement in vertigo
  - Measured as a dichotomous outcome (improved/not improved), according to self‐report, or according to a change of a specified score (as described by the study authors) on a vertigo rating scale.
- Change in vertigo
  - Measured as a continuous outcome, to identify the extent of change in vertigo symptoms.
- Serious adverse events
  - Including any event that causes death, is life‐threatening, requires hospitalisation, results in disability or permanent damage, or in congenital abnormality. Measured as the number of participants who experienced at least one serious adverse event during the follow‐up period.

Vertigo symptoms comprise a variety of different features, including frequency of episodes, duration of episodes and severity/intensity of the episodes. Where possible, we included data for the vertigo outcomes that encompassed all of these three aspects (frequency, duration and severity/intensity of symptoms). However, we anticipated that these data may not be available from all studies. We therefore extracted data on the frequency of vertigo episodes as an alternative measure for these outcomes. 

##### Secondary outcomes

- Disease‐specific health‐related quality of life
  - Measured with the Dizziness Handicap Inventory (DHI, Jacobsen 1990), a validated measurement scale in widespread use. If data from the DHI were unavailable we extracted data from alternative validated measurement scales, according to the order of preference described in the list below (based on the validity of the scales for this outcome):
    - DHI short form (Tesio 1999);
    - DHI screening tool (Jacobsen 1998);
    - Vertigo Handicap Questionnaire (Yardley 1992a);
    - Ménière's Disease Patient Oriented Symptoms Inventory (MDPOSI, Murphy 1999);
    - University of California Los Angeles Dizziness Questionnaire (UCLADQ, Honrubia 1996);
    - AAO‐HNS Functional Level Scale (FLS, AAO‐HNS 1995).
- Hearing
  - Measured with pure tone audiometry and reported as the change in pure tone average (PTA), or (alternatively) by patient report, if data from PTA were not available.
- Tinnitus
  - Measured using any validated, patient‐reported questionnaire relating to the impact of tinnitus, for example the Tinnitus Handicap Inventory (THI, Newman 1996) or the Tinnitus Functional Index (TFI, Meikle 2012). We included data that considered the impact of tinnitus on quality of life; not assessments of the loudness, pitch or frequency of tinnitus. 
- Other adverse effects
  - Measured as the number of participants who experienced at least one episode of the specified adverse events during the follow‐up period. This included the following specified adverse effects:
    - Headache
    - Gastrointestinal disturbance (including nausea, indigestion, abdominal pain or diarrhoea)
    - Sleep disturbance (including drowsiness or insomnia)
    - Dry mouth
    - Steroid‐related side effects (including increased appetite, weight gain, abnormalities of blood sugar, mood disturbance, hypertension or Cushing's syndrome).

### Search methods for identification of studies

The Cochrane ENT Information Specialist conducted systematic searches for randomised controlled trials and controlled clinical trials in October 2021 and September 2022. There were no language, publication year or publication status restrictions. The date of the latest search was 14 September 2022. 

#### Electronic searches

The Information Specialist searched:

- the Cochrane ENT Trials Register (search via the Cochrane Register of Studies to 14 September 2022);
- the Cochrane Central Register of Controlled Trials (CENTRAL) (search via the Cochrane Register of Studies to 14 September 2022);
- Ovid MEDLINE(R) Epub Ahead of Print, In‐Process & Other Non‐Indexed Citations, Ovid MEDLINE(R) Daily and Ovid MEDLINE(R) (1946 to 14 September 2022);
- Ovid Embase (1974 to 14 September 2022);
- Web of Knowledge, Web of Science (1945 to 14 September 2022);
- ClinicalTrials.gov, www.clinicaltrials.gov (to 14 September 2022);
- World Health Organization (WHO) International Clinical Trials Registry Platform (ICTRP), https://trialsearch.who.int/ (to 14 September 2021).

The Information Specialist modelled subject strategies for databases on the search strategy designed for CENTRAL. The strategy has been designed to identify all relevant studies for a suite of reviews on various interventions for Ménière's disease. Where appropriate, they were combined with subject strategy adaptations of the highly sensitive search strategy designed by Cochrane for identifying randomised controlled trials and controlled clinical trials (as described in the *Cochrane Handbook for Systematic Reviews of Interventions* Version 5.1.0, Box 6.4.b (Handbook 2011). Search strategies for major databases including CENTRAL are provided in Appendix 2.

#### Searching other resources

We scanned the reference lists of identified publications for additional trials and contacted trial authors where necessary. In addition, the Information Specialist searched Ovid MEDLINE to retrieve existing systematic reviews relevant to this systematic review, so that we could scan their reference lists for additional trials. In addition, the Information Specialist ran a non‐systematic search of Google Scholar to identify trials not published in mainstream journals. 

We did not perform a separate search for adverse effects. We considered adverse effects described in included studies only.

### Data collection and analysis

#### Selection of studies

The Cochrane ENT Information Specialist used the first two components of Cochrane's Screen4Me workflow to help assess the search results: 

1. Known assessments – a service that matches records in the search results to records that have already been screened in Cochrane Crowd and been labelled as 'a RCT' or as 'not a RCT'. 
2. The machine learning classifier (RCT model) (Wallace 2017), available in the Cochrane Register of Studies (CRS‐Web), which assigns a probability of being a true RCT (from 0 to 100) to each citation. Citations that were assigned a probability score below the cut‐point at a recall of 99% were assumed to be non‐RCTs. We manually dual screened the results for those that scored on or above the cut‐point. 

At least two review authors (KG, KW) or co‐workers (BG, AL, SC listed in Acknowledgements) independently screened the remaining titles and abstracts using Covidence, to identify studies that may be relevant for the review. Any discrepancies were resolved by consensus, or by retrieving the full text of the study for further assessment. 

We obtained the full text for any study that was considered possibly relevant and two authors (KG, KW) or co‐workers (BG, AL) again independently checked this to determine whether it met the inclusion criteria for the review. Any differences were resolved by discussion and consensus, or through recourse to a third author if necessary. 

We excluded any studies that were retrieved in full text but subsequently deemed to be inappropriate for the review (according to the inclusion/exclusion criteria), according to the main reason for exclusion. 

The unit of interest for the review is the study, therefore multiple papers or reports of a single study are grouped together under a single reference identification. The process for study selection is recorded in Figure 1. 

**1:**
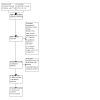
Flow chart of study retrieval and selection.

##### Screening eligible studies for trustworthiness

We assessed studies meeting our inclusion criteria for trustworthiness using a screening tool developed by Cochrane Pregnancy and Childbirth. This tool includes specified criteria to identify studies that are considered sufficiently trustworthy to be included in the review (see Appendix 3 and Figure 2). If studies were assessed as being potentially 'high‐risk', we attempted to contact the study authors to obtain further information or address any concerns. We planned to exclude studies from the main analyses of the review if there were persisting concerns over trustworthiness, or we were unable to contact the authors. However, over the course of the review it became apparent that the majority of included studies had some concerns ‐ typically due to missing information that was not reported in the original study publications. 

**2:**
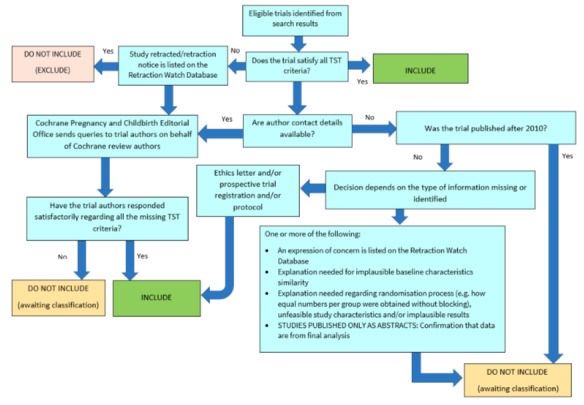
The Cochrane Pregnancy and Childbirth Trustworthiness Screening Tool

When using the trustworthiness tool, we had no concerns about two studies (Adrion 2016; Derebery 2004). Three studies were published after 2010 but did not have a registered protocol, or the authors were unable to supply us with a copy of the trial protocol (Albu 2016; Khan 2011; Park 2016). Five studies had an equal number of participants allocated to each group, but did not report the use of blocked randomisation, which may highlight a concern with the randomisation process (Albu 2016; Khan 2011; Park 2016; Ricci 1987; Schmidt 1992). Three studies provided very limited baseline information on participants with Ménière's disease, which was insufficient for us to determine whether there may have been issues with randomisation (Khan 2011; Mira 2003; Schmidt 1992). One study reported no loss to follow‐up at all (Ricci 1987), and two studies reported very substantial effect sizes, despite the relatively small size of the trials (Khan 2011; Morales‐Luckie 2005). 

We attempted to contact authors to clarify these issues, but we either received no reply, or the authors were unable to access the original trial data to clarify our queries. We had not anticipated this issue when drafting the protocol for our review, but it is likely to be a widespread issue for reviews that incorporate older studies.

There are several possible explanations for the large number of studies that had concerns when using the tool. One is that there are issues with the trustworthiness of the studies identified in this review, and the data included may not give reliable estimates of the true effect. Alternatively, the trustworthiness screening tool may be excessively sensitive, and flag studies that are trustworthy, but where information has not been fully reported. We note that this tool (and others used for the same purpose) has not yet been validated for use. 

We therefore took the decision to include the studies in the review, despite the potential concerns over trustworthiness. The uncertainty in the results is captured as part of our GRADE rating in the certainty of the evidence, using the domain 'study limitations'. 

#### Data extraction and management

Two review authors (KG, KW) independently extracted outcome data from each study using a standardised data collection form. Where a study had more than one publication, we retrieved all publications to ensure that we had a complete data set. We checked any discrepancies in the data extracted by the two authors against the original reports, and resolved differences through discussion and consensus. If required, we contacted the study authors for clarification.

We extracted data on the key characteristics of the studies, including the following information:

- study design, duration of the study, number of study centres and location, study setting and dates of the study;
- information on the participants, including the number randomised, those lost to follow‐up or withdrawn, the number analysed, the age of participants, gender, severity of the condition, diagnostic criteria used, inclusion and exclusion criteria for the individual studies;
- details of the intervention, comparator, and concomitant treatments or excluded medications;
- the outcomes specified and reported by the study authors, including the time points;
- funding for the study and any conflicts of interest for the study authors;
- information required to assess the risk of bias in the study, and to enable GRADE assessment of the evidence.

Once the extracted data were checked and any discrepancies resolved, a single author transferred the information to Review Manager 5 (RevMan 2020). 

The primary effect of interest for this review is the effect of treatment assignment (which reflects the outcomes of treatment for people who were assigned to the intervention) rather than a per protocol analysis (the outcomes of treatment only for those who completed the full course of treatment as planned). For the outcomes of interest in this review, we extracted the findings from the studies on an available case basis, i.e. all available data from all participants at each time point, based on the treatment to which they were randomised. This was irrespective of compliance, or whether participants had received the intervention as planned.

In addition to extracting pre‐specified information about study characteristics and aspects of methodology relevant to risk of bias, we extracted the following summary statistics for each study and outcome:

- For continuous data: the mean values, standard deviation and number of patients for each treatment group at the different time points for outcome measurement. Where change‐from‐baseline data were not available, we extracted the values for endpoint data instead. If values for the individual treatment groups were not reported, where possible we extracted summary statistics (e.g. mean difference) from the studies.
- For binary data: we extracted information on the number of participants experiencing an event, and the number of participants assessed at that time point. If values for the individual treatment groups were not reported, where possible we extracted summary statistics (e.g. risk ratio) from the studies.
- For ordinal scale data: if the data appeared to be normally distributed, or if the analysis performed by the investigators indicated that parametric tests are appropriate, then we treated the outcome measure as continuous data. Alternatively, if data were available, we converted these to binary data for analysis ‐ for example, for analysis of improvement in vertigo, when rated using the AAO‐HNS 1995 control of vertigo scale. 
- For time‐to‐event data: we did not identify any time‐to‐event data for the outcomes specified in the review. 

If necessary, we converted data found in the studies to a format appropriate for meta‐analysis, according to the methods described in the *Cochrane Handbook for Systematic Reviews of Interventions* (Handbook 2021). 

We pre‐specified time points of interest for the outcomes in this review. Where studies reported data at multiple time points, we took the longest available follow‐up point within each of the specific time frames. For example, if a study reported an outcome at 12 weeks and 20 weeks of follow‐up then we included the 20‐week data for the time period 3 to 6 months (12 to 24 weeks).

#### Assessment of risk of bias in included studies

Two authors (KG, KW) undertook assessment of the risk of bias of the included studies independently, with the following taken into consideration, as guided by the *Cochrane Handbook for Systematic Reviews of Interventions* (Handbook 2011):

- sequence generation;
- allocation concealment;
- blinding;
- incomplete outcome data;
- selective outcome reporting; and
- other sources of bias.

We used the Cochrane risk of bias tool (Handbook 2011), which involves describing each of these domains as reported in the study and then assigning a judgement about the adequacy of each entry: 'low', 'high' or 'unclear' risk of bias.

#### Measures of treatment effect

We summarised the effects of the majority of dichotomous outcomes (e.g. serious adverse effects) as risk ratios (RR) with 95% confidence intervals (CIs). We have also expressed the results as absolute numbers based on the pooled results and compared to the assumed risk in the summary of findings tables (Table 1; Table 2) and full GRADE profiles (Table 3; Table 4; Table 5; Table 6). 

**1:** GRADE profile: Betahistine for Ménière's disease

**Certainty assessment**							**Number of participants**		**Effect**		**Certainty**	**Comment**
**№ of studies**	**Study design**	**Risk of bias**	**Inconsistency**	**Indirectness**	**Imprecision**	**Other considerations**	**Betahistine**	**Placebo/no treatment**	**Relative** **(95% CI)**	**Absolute** **(95% CI)**		
**Improvement in vertigo frequency (follow‐up: range 6 months to ≤12 months; assessed with: Self‐rated improvement in either frequency or severity of vertigo)**												
1	randomised trials	very serious^a^	not serious	serious^b,c^	serious^d,e^	none	24/35 (68.6%)	16/35 (45.7%)	**RR 1.50** (0.98 to 2.29)	**229 more per 1000** (from 9 fewer to 590 more)	⊕⊝⊝⊝ Very low	
**Improvement in vertigo frequency (follow‐up: range >12 months; assessed with: AAO‐HNS 1995 class A, B or C)**												
1	randomised trials	serious^f^	not serious	serious^g^	serious^d,e^	none	28/30 (93.3%)	27/32 (84.4%)	**RR 1.11** (0.93 to 1.32)	**93 more per 1000** (from 59 fewer to 270 more)	⊕⊝⊝⊝ Very low	
**Improvement in vertigo frequency: sensitivity analysis for complete/substantial improvement (follow‐up: range 6 months to ≤ 12 months; assessed with: AAOO 1972 class A, B or C (complete resolution of vertigo)**												
1	randomised trials	serious^h^	not serious	serious^b,i^	very serious^j^	none	3/5 (60.0%)	1.0%	**Peto OR 13.08** (1.01 to 170.31)	**107 more per 1000** (from 0 fewer to 622 more)	⊕⊝⊝⊝ Very low	
								10.0%		**492 more per 1000** (from 1 more to 850 more)		
**Improvement in vertigo frequency: sensitivity analysis for complete/substantial improvement (follow‐up: range > 12 months; assessed with: AAO‐HNS 1995 class A or B)**												
1	randomised trials	serious^f^	not serious	serious^g^	serious^d,e^	none	27/30 (90.0%)	21/32 (65.6%)	**RR 1.37** (1.04 to 1.81)	**243 more per 1000** (from 26 more to 532 more)	⊕⊝⊝⊝ Very low	
**Vertigo global score (follow‐up: range 3 months to < 6 months; assessed with: geometric mean of monthly imbalance score)**												
1	randomised trials	serious^k^	not serious	not serious	very serious^d,l^	none	17	17	—	MD **0.7 points higher** (6.67 lower to 8.07 higher)	⊕⊝⊝⊝ Very low	
**Vertigo frequency (follow‐up: range 3 months to < 6 months; assessed with: number of attacks per month)**												
2	randomised trials	serious^m^	not serious	not serious	very serious^d,e,n^	none	60	57	—	MD **1.90 attacks per month lower** (3.05 lower to 0.74 lower)	⊕⊝⊝⊝ Very low	
**Vertigo frequency (follow‐up: range 6 months to ≤ 12 months; assessed with: average number of attacks in 30 days)**												
1	randomised trials	serious^o^	not serious	not serious	very serious^d,l^	none	142	72	—	MD **0.63 attacks per 30 days higher** (4.07 lower to 5.33 higher)	⊕⊝⊝⊝ Very low	
**Serious adverse events**												
1	randomised trials	serious^o^	not serious	not serious	very serious^d,l^	none	26/146 (17.8%)	11/74 (14.9%)	**RR 1.20** (0.63 to 2.29)	**30 more per 1000** (from 55 fewer to 192 more)	⊕⊝⊝⊝ Very low	
**Change in disease‐specific health‐related quality of life (follow‐up: range 6 months to ≤ 12 months; assessed with: Dizziness Handicap Inventory (mean score per question); scale from: 0 to 4)**												
1	randomised trials	serious^o^	not serious	not serious	serious^d^	none	114	56	—	MD **0.06 points higher** (0.17 lower to 0.29 higher)	⊕⊕⊝⊝ Low	
**Change in disease‐specific health‐related quality of life (follow‐up: range > 12 months; assessed with: Functional Level Scale, score 1 or 2)**												
1	randomised trials	serious^f^	not serious	serious^g^	serious^d,e^	none	29/30	23/32	**RR 1.34** (1.07 to 1.69)	**244 more per 1000** (from 50 more to 496 more)	⊕⊝⊝⊝ Very low	
**Change in hearing: continuous data only (follow‐up: range 3 months to < 6 months; assessed with: hearing threshold with pure tone audiometry)**												
1	randomised trials	serious^k^	not serious	not serious	serious^d,e^	none	18	17	—	MD **10.1 dB HL higher** (1.13 lower to 21.33 higher)	⊕⊕⊝⊝ Low	
**Change in hearing: continuous data only (follow‐up: range 6 months to ≤ 12 months; assessed with: change in PTA)**												
1	randomised trials	serious^o^	not serious	not serious	very serious^d,p^	none	79	34	—	MD **2.64 dB higher** (1.66 lower to 6.94 higher)	⊕⊝⊝⊝ Very low	
**Change in hearing: continuous data only (follow‐up: range > 12 months; assessed with: hearing threshold with PTA)**												
1	randomised trials	serious^f^	not serious	serious^g^	serious^d^	none	30	32	—	MD **1.4 dB HL higher** (7.1 lower to 9.9 higher)	⊕⊝⊝⊝ Very low	
**Change in hearing: dichotomous data only (follow‐up: range 6 months to ≤ 12 months; assessed with: improvement with pure tone audiometry)**												
2	randomised trials	very serious^q^	not serious	serious^b,r^	serious^d,e^	none	24/41 (58.5%)	13/41 (31.7%)	**Peto OR 3.14** (1.28 to 7.66)	**276 more per 1000** (from 56 more to 463 more)	⊕⊝⊝⊝ Very low	
**Change in tinnitus ‐ 6 to ≤ 12 months (assessed with: MiniTF score; scale from: 0 to 24)**												
1	randomised trials	serious^o^	not serious	not serious	serious^d^	none	114	54	—	MD **0.06 lower** (1.52 lower to 1.39 higher)	⊕⊕⊝⊝ Low	
**Tinnitus (follow‐up: range ≥ 12 months to 0; assessed with: THI; scale from: 0 to 100)**												
1	randomised trials	serious^f^	not serious	serious^g^	serious^d^	none	30	32	—	MD **0.9 points higher** (5.55 lower to 7.35 higher)	⨁◯◯◯ Very low	
**Other adverse effects ‐ headache**												
4	randomised trials	serious^s^	serious^t^	not serious	very serious^d,l^	none	62/226 (27.4%)	31/148 (20.9%)	**OR 1.16** (0.69 to 1.95)	**26 more per 1000** (from 55 fewer to 131 more)	⊕⊝⊝⊝ Very low	
**Other adverse effects ‐ gastrointestinal disturbance**												
4	randomised trials	serious^s^	serious^t^	not serious	very serious^d,l^	none	79/224 (35.3%)	41/148 (27.7%)	**OR 1.08** (0.65 to 1.78)	**16 more per 1000** (from 78 fewer to 128 more)	⊕⊝⊝⊝ Very low	
**Other adverse effects ‐ dry mouth**												
2	randomised trials	serious^s^	not serious	not serious	serious^d^	none	2/187 (1.1%)	3/114 (2.6%)	**OR 0.30** (0.05 to 1.95)	**18 fewer per 1000** (from 25 fewer to 24 more)	⊕⊕⊝⊝ Low	
**Other adverse effects ‐ sleep disturbance**												
2	randomised trials	serious^u^	not serious	not serious	very serious^d,l^	none	7/164 (4.3%)	4/91 (4.4%)	**RR 1.42** (0.47 to 4.38)	**18 more per 1000** (from 23 fewer to 149 more)	⊕⊝⊝⊝ Very low	

**AAO‐HNS:** American Academy of Otolaryngology – Head and Neck Surgery; **AAOO:** American Academy of Ophthalmology and Otolaryngology; **CI:** confidence interval; **MD:** mean difference; **OR:** odds ratio; **PTA:** pure tone average; **RR:** risk ratio ^a^High risk of bias for five domains in this study, and unclear risk of bias for the remaining two domains. ^b^The criteria used for the diagnosis of Ménière's disease were poorly defined, therefore the population may not be appropriate. ^c^This outcome was reported as an improvement in either the frequency or severity of attacks, not only frequency. ^d^Optimal information size was not reached (taken as < 300 events for dichotomous outcomes or < 400 participants for continuous outcomes, as a rule of thumb). ^e^Confidence interval ranges from a likely trivial effect to potential benefit. ^f^Unclear risk of bias for several domains, and high risk of bias due to differential use of intratympanic steroids in the intervention and control group. ^g^All participants also received intratympanic dexamethasone injections throughout the trial. ^h^Multiple bias domains unclear, and high risk of selective reporting. ^i^Scoring system for vertigo only considers "complete resolution", not substantial improvement. ^j^Sample size extremely small and confidence interval ranges from potential harm to potential benefit. ^k^Multiple domains at unclear risk of bias leading to an overall concern about the risk for this trial. ^l^Confidence interval ranges from potential harm to potential benefit. ^m^Multiple bias domains rated at unclear risk of bias. High risk of selective reporting bias due to incomplete outcome data for this result. ^n^Numeric data used in this analysis were estimated due to incomplete reporting in the article. ^o^High risk of attrition bias, and potential for selective reporting. ^p^Data for four‐tone average estimated from reported data at each of the four frequencies. ^q^High risk of bias for multiple domains in both of the included studies. ^r^The trial with the largest weight in the analysis assessed the "better hearing side", which may not be appropriate (likely to be the ear without Ménière's disease). ^s^Risk of bias rated as either high risk or unclear risk for several domains in the studies. ^t^I^2^ > 40%. ^u^The trial with the largest weight in the analysis has multiple concerns regarding risk of bias.

**2:** GRADE profile: Diuretic versus no treatment/placebo for Ménière's disease

**Certainty assessment**							**Number of participants**		**Effect**	** **	**Certainty**	**Comment**
**№ of studies**	**Study design**	**Risk of bias**	**Inconsistency**	**Indirectness**	**Imprecision**	**Other considerations**	**Diuretic**	**Placebo/no treatment**	**Relative** **(95% CI)**	**Absolute** **(95% CI)**		
**Improvement in vertigo frequency (follow‐up: range 6 months to ≤ 12 months; assessed with: self‐rated improvement in either frequency or severity of vertigo)**												
1	randomised trials	very serious^a^	not serious	serious^b,c^	serious^d^	none	27/35 (77.1%)	16/35 (45.7%)	**RR 1.69** (1.13 to 2.53)	**315 more per 1000** (from 59 more to 699 more)	⊕⊝⊝⊝ Very low	
**Change in vertigo frequency (follow‐up: range 3 months to ≤ 6 months; assessed with: number of episodes during a 4 week‐period)**												
1	randomised trials	very serious^e^	not serious	serious^f^	serious^d,g^	none	110	110	—	MD **2.44 episodes per 4 weeks lower** (4.98 lower to 0.1 higher)	⊕⊝⊝⊝ Very low	
**Change in disease‐specific health‐related quality of life (follow‐up: range 3 months to < 6 months; assessed with: Korean DHI; scale from: 0 to 100)**												
1	randomised trials	very serious^e^	not serious	serious^f^	serious^d^	none	110	110	—	MD **2.94 points higher** (3.86 lower to 9.74 higher)	⊕⊝⊝⊝ Very low	
**Change in hearing: continuous data only (follow‐up: range 3 months to < 6 months; assessed with: PTA change in hearing threshold)**												
1	randomised trials	very serious^e^	not serious	serious^f^	serious^d^	none	110	110	—	MD **0.94 dB HL lower** (3.84 lower to 1.96 higher)	⊕⊝⊝⊝ Very low	
**Change in hearing: dichotomous data only (follow‐up: range 6 months to ≤ 12 months; assessed with: ≥ 10dB improvement with PTA on the "better hearing side")**												
1	randomised trials	very serious^a^	not serious	serious^b,h^	serious^d^	none	23/36 (63.9%)	13/36 (36.1%)	**RR 1.77** (1.07 to 2.91)	**278 more per 1000** (from 25 more to 690 more)	⊕⊝⊝⊝ Very low	
**Change in tinnitus (follow‐up: range 3 months to < 6 months; assessed with: Korean THI; scale from: 0 to 100)**												
1	randomised trials	very serious^e^	not serious	serious^f^	serious^d^	none	110	110	—	MD **1.89 points higher** (4.96 lower to 8.74 higher)	⊕⊝⊝⊝ Very low	

**CI:** confidence interval; **DHI:** Dizziness Handicap Inventory; **MD:** mean difference; **PTA:** pure tone average; **RR:** risk ratio ^a^High risk of bias for five domains in this study, and unclear risk of bias for the remaining two domains. ^b^The criteria used for the diagnosis of Ménière's disease were poorly defined, therefore the population may not be appropriate. ^c^This outcome was reported as an improvement in either the frequency or severity of attacks, not only frequency. ^d^Optimal information size was not reached (taken as < 300 events for dichotomous outcomes or < 400 participants for continuous outcomes, as a rule of thumb). ^e^High risk of performance and detection bias. Unclear risk of bias for multiple domains. ^f^All participants were also taking betahistine for the duration of the trial. ^g^Confidence interval ranges from a trivial effect to potential benefit. ^h^The study assessed the "better hearing side", which may not be appropriate (likely to be the ear without Ménière's disease).

**3:** GRADE profile: Antiviral versus no treatment/placebo for Ménière's disease

**Certainty assessment**							**Number of participants**		**Effect**		**Certainty**	**Comment**
**№ of studies**	**Study design**	**Risk of bias**	**Inconsistency**	**Indirectness**	**Imprecision**	**Other considerations**	**Antiviral**	**Placebo/no treatment**	**Relative** **(95% CI)**	**Absolute** **(95% CI)**		
**Improvement in vertigo frequency (follow‐up: range 3 months to < 6 months; assessed with: reduction in number of vertigo episodes by 20% compared to baseline)**												
1	randomised trials	serious^a^	not serious	not serious	very serious^b,c,d^	none	3/12 (25.0%)	2/11 (18.2%)	**RR 1.38** (0.28 to 6.75)	**69 more per 1000** (from 131 fewer to 1000 more)	⊕⊝⊝⊝ Very low	
**Change in vertigo frequency (follow‐up: range 3 months to < 6 months; assessed with: frequency of dizzy episodes per week)**												
1	randomised trials	serious^a^	not serious	not serious	very serious^b,c,d^	none	12	11	—	MD **0.1 episodes per week higher** (1.03 lower to 1.23 higher)	⊕⊝⊝⊝ Very low	
**Disease‐specific health‐related quality of life (follow‐up: range 3 months to < 6 months; assessed with: DHI; scale from: 0 to 100)**												
1	randomised trials	serious^a^	not serious	not serious	serious^b,d^	none	11	10	—	MD **7.4 points higher** (15.78 lower to 30.58 higher)	⊕⊕⊝⊝ Low	
**Hearing at 3 to < 6 months**												
1	randomised trials	serious^a^	not serious	not serious	very serious^b,c,d^	none	9	7	—	MD **4.3 dB HL higher** (13.94 lower to 22.54 higher)	⊕⊝⊝⊝ Very low	

**CI:** confidence interval; **MD:** mean difference; **RR:** risk ratio ^a^Unclear risk of bias for multiple domains (randomisation, performance and detection bias). Potential for selective reporting bias (outcomes at 6 months not available). ^b^Optimal information size was not reached (taken as < 300 events for dichotomous outcomes or < 400 participants for continuous outcomes, as a rule of thumb). ^c^Confidence interval ranges from potential harm to potential benefit. ^d^Extremely small sample size.

**4:** GRADE profile: Corticosteroids versus no treatment/placebo for Ménière's disease

**Certainty assessment**							**Number of participants**		**Effect**		**Certainty**	**Importance**
**№ of studies**	**Study design**	**Risk of bias**	**Inconsistency**	**Indirectness**	**Imprecision**	**Other considerations**	**Corticosteroids**	**Placebo/no treatment**	**Relative** **(95% CI)**	**Absolute** **(95% CI)**		
**Improvement in vertigo (follow‐up: range > 12 months; assessed with: AAO‐HNS class A, B or C)**												
1	randomised trials	very serious^a^	not serious	serious^b^	serious^c,d^	none	8/8 (100.0%)	8/8 (100.0%)	**RR 1.00** (0.80 to 1.25)	**0 fewer per 1000** (from 200 fewer to 250 more)	⊕⊝⊝⊝ Very low	
**Improvement in vertigo: sensitivity analysis for complete/substantial improvement (follow‐up: range > 12 months; assessed with: AAO‐HNS class A or B)**												
1	randomised trials	very serious^a^	not serious	serious^b^	serious^c^	none	8/8 (100.0%)	1.0%	**Peto OR 42.52** (6.37 to 283.65)	**290 more per 1000** (from 50 more to 731 more)	⊕⊝⊝⊝ Very low	
								10.0%		**725 more per 1000** (from 314 more to 869 more)		
**Change in vertigo frequency (follow‐up: range 3 months to < 6 months; assessed with: number of episodes per day)**												
1	randomised trials	very serious^a^	not serious	serious^b^	serious^c^	none	8	8	—	MD **0.44 episodes per day fewer** (0.7 fewer to 0.18 fewer)	⊕⊝⊝⊝ Very low	
**Disease‐specific health‐related quality of life (follow‐up: range > 12 months; assessed with: number of people in whom the FLS improved)**												
1	randomised trials	very serious^a^	not serious	serious^b^	serious^c^	none	7/8 (87.5%)	1.0%	**Peto OR 28.03** (4.14 to 189.82)	**211 more per 1000** (from 00 more to 647 more)	⊕⊝⊝⊝ Very low	
								10.0%		**657 more per 1000** (from 215 more to 855 more)		
**Other adverse effects ‐ steroid‐related side effects**												
1	randomised trials	very serious^a^	not serious	serious^b^	very serious^c,d^	none	1/8 (12.5%)	1.0%	**Peto OR 7.39** (0.15 to 372.38)	**59 more per 1000** (from 8 fewer to 780 more)	⊕⊝⊝⊝ Very low	
								10.0%		**351 more per 1000** (from 84 fewer to 876 more)		

**CI:** confidence interval; **FLS:** Functional Level Scale; **MD:** mean difference; **OR:** odds ratio; **RR:** risk ratio ^a^High risk of performance and detection bias. Potential for selective reporting. ^b^Criteria for diagnosis of Ménière's disease are not fully described. All participants received background interventions of diphenidol and acetazolamide. ^c^Optimal information size was not reached (taken as < 300 events for dichotomous outcomes or < 400 participants for continuous outcomes, as a rule of thumb). ^d^Confidence interval ranges from potential harm to potential benefit.

The reported event rate was zero for some outcomes. Therefore, we used the Peto odds ratio (OR) to analyse these data, according to the guidance in Xu 2021, as this should produce less biased estimates of the effect size when events are rare (as described in the Handbook 2021). 

For continuous outcomes, we expressed treatment effects as a mean difference (MD) with standard deviation (SD). We did not need to use the standardised mean difference to pool any data. 

Hearing data for Adrion 2016 were reported using the hearing threshold at four different frequencies, rather than an average hearing threshold. We therefore use the reported data to re‐create an estimated summary measure for the four frequencies, as described in Borenstein 2009. Hearing thresholds for each of these frequencies in an individual may be correlated, but we were unable to identify a published correlation coefficient to use for these calculations. We therefore assumed complete correlation between the different frequencies, which should provide a conservative estimate of the variance for the summary effect. 

#### Unit of analysis issues

Ménière's disease is unlikely to be a stable condition, and interventions may not have a temporary effect. Therefore, we only used data from the first phase of cross‐over studies. If these data were not available then the study was excluded from the review. No cluster‐randomised trials were identified as being suitable for inclusion.

We identified two studies with three arms, and ensured that these were included whilst avoiding double‐counting of any participants. One study contributed to separate comparisons in the review (betahistine, diuretics and placebo; Khan 2011), therefore we included the placebo group for each analysis. One study related to the same comparison (low‐dose betahistine, high‐dose betahistine and placebo; Adrion 2016), and we included these data by pooling the relevant intervention arms (according to the methods in the Handbook 2021). 

#### Dealing with missing data

We planned to contact study authors via email whenever the outcome of interest was not reported, if the methods of the study suggest that the outcome had been measured. We did the same if not all data required for meta‐analysis were reported (for example, standard deviations), unless we were able to calculate them from other data reported by the study authors. 

#### Assessment of heterogeneity

We assessed clinical heterogeneity by examining the included studies for potential differences between them in the types of participants recruited, interventions or controls used and the outcomes measured. This is highlighted in the Included studies section, below.

We used the I^2^ statistic to quantify inconsistency amongst the studies in each meta‐analysis. We also considered the P value from the Chi^2^ test. However, we conducted few meta‐analyses in the course of this review, and we did not identify any serious inconsistency. 

#### Assessment of reporting biases

We assessed reporting bias as within‐study outcome reporting bias and between‐study publication bias.

##### Outcome reporting bias (within‐study reporting bias)

We assessed within‐study reporting bias by comparing the outcomes reported in the published report against the study protocol or trial registry, whenever this could be obtained. If the protocol or trial registry entry was not available, we compared the outcomes reported to those listed in the methods section. If results are mentioned but not reported adequately in a way that allows analysis (e.g. the report only mentions whether the results were statistically significant or not), bias in a meta‐analysis is likely to occur. We then sought further information from the study authors. If no further information was found, we noted this as being a 'high' risk of bias with the risk of bias tool. If there was insufficient information to judge the risk of bias we noted this as an 'unclear' risk of bias (Handbook 2011). 

##### Publication bias (between‐study reporting bias)

We did not have sufficient studies to create funnel plots for any analysis. Any studies identified through trial registries and other sources (Searching other resources) that remain unpublished are noted in the Ongoing studies section. 

#### Data synthesis

##### Meta‐analysis of numerical data

Where possible and appropriate (if participants, interventions, comparisons and outcomes were sufficiently similar in the trials identified) we conducted a quantitative synthesis of results. We conducted all meta‐analyses using RevMan 2020. We anticipated that the underlying effect of the intervention may vary between studies, due to differences between participants, settings and the interventions used for each study. We planned to use a random‐effects model for meta‐analysis and explore whether the use of a fixed‐effect model substantially alters the effect estimates (see Sensitivity analysis). However, we were only able to use the Peto OR (a fixed‐effect method) for all meta‐analysis in this review, due to rare or zero events in at least one of the studies included in the analysis.

We did not conduct any meta‐analysis for continuous outcomes in this review. 

Improvement in vertigo symptoms may be assessed using a variety of methods, which consider different aspects of vertigo. These include:

- frequency of vertigo episodes;
- duration of vertigo episodes;
- severity/intensity of vertigo episodes;
- a composite measure of all of these aspects:
  - for example, assessed with a global score ‐ such as "how troublesome are your vertigo symptoms?", rated on an ordinal scale.

For the outcomes "improvement in vertigo" and "change in vertigo", we prioritised outcome measures that used a composite score ‐ encompassing aspects of vertigo frequency, duration and severity/intensity. Examples of this would include a global rating scale of vertigo impact (rated from 0 to 10, where 0 is defined as no symptoms, and 10 is defined as the most troublesome symptoms) or the vertigo/balance subscale of the Vertigo Symptom Scale (Yardley 1992b), or Vertigo Symptom Scale Short Form (Yardley 1998). As data from composite scores were not available from the majority of studies, we also included data on the frequency of vertigo episodes as an alternative measure.

##### Synthesis using other methods

If we were unable to pool numerical data in a meta‐analysis for one or more outcomes we planned to provide a synthesis of the results using alternative methods, following the guidance in chapter 12 of the Handbook 2021. However, this was not necessary, as results were typically provided by a single study. 

#### Subgroup analysis and investigation of heterogeneity

If statistical heterogeneity was identified for any comparison, we planned to assess this considering the following subgroups:

- different types of medication, within a specific class;
- different doses/frequency of administration;
- use of concomitant treatment;
- diagnosis of Ménière's disease

However, due to the paucity of data available, and the few meta‐analyses included in this review, we did not carry out any subgroup analysis. 

#### Sensitivity analysis

We planned to carry out a number of sensitivity analyses for the primary outcomes in this review. However, the paucity of data and the lack of meta‐analyses has meant that this was not possible. 

If few studies are identified for meta‐analysis, the random‐effects model may provide an inaccurate measure of the between‐studies variance. Therefore, we planned to explore the impact of using a fixed‐effect model using a sensitivity analysis. However, few meta‐analyses were conducted, and these analyses were actually carried out using the Peto OR, a fixed‐effect method, due to zero events in at least one arm of a study. For completeness, we have compared the results to a random‐effects method using the Mantel‐Haenzel OR, but the results are very similar (Table 7).

**5:** Sensitivity analysis

**Analysis**	**Main analysis result**	**Method of sensitivity analysis**	**Sensitivity analysis result**
Analysis 1.10	Peto OR 3.14 (95% CI 1.28 to 7.66)	Random‐effects, Mantel Haenszel OR	OR 3.17 (95% CI 1.25 to 7.99)*
Analysis 1.12 (headache)	Peto OR 2.34 (95% CI 0.72 to 7.58)	Random‐effects, Mantel Haenszel OR	OR 2.54 (95% CI 0.19 to 4.50)*
Analysis 1.12 (gastrointestinal disturbance)	Peto OR 1.63 (95% CI 0.39 to 6.84)	Random‐effects, Mantel Haenszel OR	OR 1.60 (95% CI 0.05 to 54.71)*

* Note that the primary analysis uses a Peto OR due to the occurrence of zero events in one arm of one study. Therefore, we have assessed the impact of changing to a random‐effects analysis using a Mantel‐Haenszel OR (as the Peto OR cannot use random‐effects).

If there was uncertainty over the diagnostic criteria used for participants in the studies (for example, if it was not clear whether participants were diagnosed using criteria that are analogous to the AAO‐HNS criteria) then we also planned to explore this by including/excluding those studies from the analysis. However, as noted above we had such sparse data in the review that we were unable to conduct these analyses. 

We used the Cochrane Pregnancy and Childbirth Screening Tool to identify any studies with concerns over the data available. We had intended that any studies identified by the tool would be excluded from the main analyses in the review, but that we would explore the impact of including the data from these studies through a sensitivity analysis. However, as noted above, we had some concerns over the use of this tool, and few studies were included in the review, therefore this sensitivity analysis was not conducted. 

We did conduct one sensitivity analysis that was not pre‐specified in our protocol (Webster 2021b). When drafting the protocol for this review we stated "improvement in vertigo" as our outcome. However, over the course of the review it became apparent that "any improvement" may not represent a meaningful improvement for people with Ménière's disease. For example, an individual who suffered 100 vertigo attacks per year at baseline and then only 99 attacks per year at follow‐up could be stated to have 'improved' ‐ although it is not clear whether the difference would be of any importance. For our main analysis for this outcome we considered 'any improvement' in vertigo, but we also conducted a sensitivity analysis to see if the effect estimates were altered if we considered 'substantial improvement' in vertigo. 

#### Summary of findings and assessment of the certainty of the evidence

Two independent authors (KG, KW) used the GRADE approach to rate the overall certainty of evidence using GRADEpro GDT (https://gradepro.org/) and the guidance in chapter 14 of the *Cochrane Handbook for Systematic Reviews of Interventions* (Handbook 2021). Disagreements were resolved through discussion and consensus. The certainty of evidence reflects the extent to which we are confident that an estimate of effect is correct, and we have applied this in the interpretation of results. There are four possible ratings: high, moderate, low and very low. A rating of high certainty of evidence implies that we are confident in our estimate of effect and that further research is very unlikely to change our confidence in the estimate of effect. A rating of very low certainty implies that any estimate of effect obtained is very uncertain.

The GRADE approach rates evidence from RCTs that do not have serious limitations as high certainty. However, several factors can lead to the downgrading of the evidence to moderate, low or very low. The degree of downgrading is determined by the seriousness of these factors:

- Study limitations (risk of bias):
  - This was assessed using the rating from the Cochrane risk of bias tool for the study or studies included in the analysis. We rated down either one or two levels, depending on the number of domains that had been rated at high or unclear risk of bias. 
- Inconsistency:
  - This was assessed using the I^2^ statistic and the P value for heterogeneity for all meta‐analyses, as well as by visual inspection of the forest plot. For results based on a single study we rated this domain as no serious inconsistency.
- Indirectness of evidence:
  - We took into account whether there were concerns over the population included in these study or studies for each outcome, as well as whether additional treatments were offered that may impact on the efficacy of the intervention under consideration. 
- Imprecision:
  - We took into account the sample size and the width of the confidence interval for each outcome. If the sample size did not meet the optimal information size (i.e. < 400 people for continuous outcomes or < 300 events for dichotomous outcomes), or the confidence interval crossed the small effect threshold, we rated down one level. If the sample size did not meet the optimal information size and the confidence interval included both potential harm and potential benefit we rated down twice. We also rated down twice for very tiny studies (e.g. 10 to 15 participants in each arm), regardless of the estimated confidence interval.
- Publication bias:
  - We considered whether there were likely to be unpublished studies that may impact on our confidence in the results obtained. 

We used a minimally contextualised approach and rated the certainty in the interventions having an important effect (Zeng 2021). Where possible, we used agreed minimally important differences (MIDs) for continuous outcomes as the threshold for an important difference. Where no MID was identified, we provide an assumed MID based on agreement between the authors. For dichotomous outcomes, we looked at the absolute effects when rating imprecision, but also took into consideration the GRADE default approach (rating down when a RR crosses 1.25 or 0.80). We have justified all decisions to downgrade the certainty of the evidence using footnotes, and added comments to aid the interpretation of the findings, where necessary. 

We provide summary of findings tables for the following comparisons:

- betahistine versus placebo/no treatment;
- diuretics versus placebo/no treatment.

We considered these two comparisons to be the most relevant and important to users of this review, therefore we prioritised these for presentation. We have included all primary outcomes in the summary of findings tables. We planned to prioritise outcomes at the time point three to six months for presentation in the tables. However, no data were available at these time points for some outcomes, and therefore we have shown the data for longer periods of follow‐up. We have also included a full GRADE profile for all results and comparisons (see Table 3; Table 4; Table 5 and Table 6).

## Results

### Description of studies

#### Results of the search

The searches in September 2022 retrieved a total of 4434 records. This reduced to 3408 after the removal of duplicates. The Cochrane ENT Information Specialist sent all 3408 records to the Screen4Me workflow. The Screen4Me workflow identified 122 records as having previously been assessed: 83 had been rejected as not RCTs and 39 had been assessed as possible RCTs. The RCT classifier rejected an additional 1427 records as not RCTs (with 99% sensitivity). We did not send any records to the Cochrane Crowd for assessment. Following this process, the Screen4Me workflow had rejected 1510  records and identified 1898 possible RCTs for title and abstract screening. 

**Table:** 

** **	**Possible RCTs**	**Rejected**
Known assessments	39	83
RCT classifier	1859	1427
Total	1898	1510

We identified 89 additional duplicates. We identified an additional eight records (linked to six studies) from handsearching of the reference lists from systematic reviews. We screened the titles and abstracts of these 1817 records. We discarded 1737 records and assessed 80 full‐text records. 

We excluded 64 records (linked to 62 studies) with reasons recorded in the review (see Excluded studies).

We identified two ongoing studies (three records). See Characteristics of ongoing studies for further details of both studies. However, it should be noted that these trials were registered more than 10 years ago and therefore are likely to have either been terminated, or been completed but remain unpublished. 

We included 10 completed studies (13 records) where results were available. A flow chart of study retrieval and selection is provided in Figure 1.

#### Included studies

We included a total of 10 RCTs (Adrion 2016; Albu 2016; Derebery 2004; Duphar B.V. 77.054/M; Khan 2011; Mira 2003; Morales‐Luckie 2005; Park 2016; Ricci 1987; Schmidt 1992). Details of individual studies can be found in the Characteristics of included studies. 

##### Study design

All included studies were described as randomised controlled trials. Most were two‐arm trials, comparing an active intervention to either placebo or no treatment. One study was a three‐armed trial comparing betahistine, diuretics and placebo (Khan 2011). The duration of follow‐up for the trials ranged from a minimum of three months (Duphar B.V. 77.054/M; Mira 2003) to a maximum of 24 months (Albu 2016). The largest trial was Adrion 2016, which randomised 221 participants, and the smallest was Ricci 1987, which randomised 10 participants.  

Most studies assessed outcomes whilst participants were continuing on active treatment. The exception to this was the study Morales‐Luckie 2005, where participants received 18 weeks of active treatment (followed by a short tapering of the steroid dose), and outcomes were predominantly assessed at 12 months' follow‐up (i.e. approximately six months after treatment had ended). The study Khan 2011 does not explicitly state the duration of treatment, but we assume that participants received treatment for 12 months (and outcomes were assessed at 12 months). 

##### Participants

All the included studies recruited adult participants with a diagnosis of Ménière's disease. 

###### Diagnosis of Ménière's disease

For most studies, the diagnosis was made according to the AAO‐HNS 1995 criteria. Three studies did not report the use of these criteria:

- Khan 2011 reported that participants were diagnosed in the ENT department of a military hospital, and required episodic vertigo (at least two definitive episodes of vertigo of at least 20 minutes duration), tinnitus and hearing loss (minimum hearing loss of 30 dB in any of 500 Hz, 1000 Hz and 2000 Hz). 
- Schmidt 1992 reported that the diagnosis was made according to the "Utrecht working definition", which comprised cochlear hearing loss, a history of tinnitus, history of attacks of vertigo and exclusion of other disease that could account for these symptoms. A specific diagnostic protocol was used to assess participants before inclusion in the study (see Characteristics of included studies for further details). 
- Ricci 1987 and Duphar B.V. 77.054/M did not describe their methods for diagnosing Ménière's disease, but as the studies were conducted in the ENT department of a hospital, we considered it likely that participants had other diagnoses excluded. 

Four studies explicitly stated that only participants with definite Ménière's disease were included (Adrion 2016; Albu 2016; Derebery 2004; Park 2016). The remaining studies did not comment on whether participants with probable disease were also included. 

###### Features of Ménière's disease

One study stated that only those with unilateral disease were included (Albu 2016), whilst three studies included participants with either unilateral or bilateral disease (Adrion 2016; Derebery 2004; Schmidt 1992). The remaining studies did not state whether participants had unilateral or bilateral disease. 

The majority of studies gave no information regarding the duration of Ménière's symptoms, and what interventions (if any) had been used before study entry. One study stated that participants had ongoing symptoms despite a six‐month trial of salt, caffeine and nicotine restriction (Albu 2016), and one stated that participants had poor vertigo control despite the use of diphenidol and acetazolamide (Morales‐Luckie 2005). One further study indicated that participants who were newly diagnosed with Ménière's disease were recruited (Khan 2011). One study excluded participants who had received intratympanic injections or surgical treatment in the preceding six months, or who had received either betahistine or isosorbide in the preceding three months (Park 2016). 

Some studies required participants to have a minimum frequency of vertigo attacks. One study required participants to have at least four attacks over the preceding three months (Park 2016). Two studies required participants to have at least two episodes per month at baseline (Adrion 2016; Derebery 2004), one study required participants to have at least four attacks per month (Albu 2016), and one study required participants to have one episode per month (Duphar B.V. 77.054/M). One study did not state a minimum requirement for attack frequency, but did report the attack frequency at baseline: approximately one attack per month (Ricci 1987). Finally, the study Morales‐Luckie 2005 appeared to recruit a group of participants with more severe disease (with reported severe disability from their symptoms, who had been offered but declined surgical intervention and who had an attack frequency of approximately one attack per day at baseline).  

##### Interventions and comparisons

The studies included addressed four of our proposed comparison pairs. No studies were identified that considered antihistamines (other than betahistine). 

Most studies compared an active intervention to a placebo. Some studies also included concomitant treatments in both groups of the trial. In Albu 2016, betahistine was compared to no treatment, but with a background treatment of intratympanic dexamethasone for all participants (i.e. the comparison was betahistine plus intratympanic steroid versus intratympanic steroid alone). Park 2016 compared isosorbide to no treatment, but with a background treatment of betahistine (i.e. the comparison was isosorbide plus betahistine versus betahistine alone). As stated in our protocol, we have included these studies ‐ as the effect they are estimating is that of the intervention of interest ‐ but we acknowledge that there may be some interaction between the intervention of interest and the background treatment. 

###### Comparison 1: Betahistine versus no treatment/placebo

Most of the included studies assessed betahistine, although the dose used in the study varied considerably:

- Ricci 1987 used 8 mg, three times daily (total daily dose 24 mg).
- Duphar B.V. 77.054/M used 12 mg, three times daily (total daily dose 36 mg).
- Mira 2003 used 16 mg twice daily (total daily dose 32 mg).
- Khan 2011 used 16 mg three times daily and Adrion 2016 used 24 mg twice daily (total daily dose 48 mg).
- Schmidt 1992 and Adrion 2016 used 24 mg three times daily (total daily dose 72 mg).
- Albu 2016 used 48 mg three times daily (total daily dose 144 mg).

Six studies compared betahistine to the use of a placebo (Adrion 2016; Albu 2016; Duphar B.V. 77.054/M; Mira 2003; Ricci 1987; Schmidt 1992). Khan 2011 used a multivitamin tablet as the comparator. The contents of the multivitamin were not described in the article. We have assumed that this may be considered a placebo, however it is possible that there would be some unknown therapeutic effect from such an intervention. 

The study Adrion 2016 included a high‐dose and low‐dose betahistine group, compared to placebo. For the purposes of this review, we have pooled data from these different doses. As noted above, the study Albu 2016 used intratympanic dexamethasone injections for all participants in the trial (those receiving betahistine and those in the control group). 

###### Comparison 2: Diuretics versus no treatment/placebo

Two studies assessed the use of diuretics, but different medications were used. Khan 2011 used a combination of 5 mg amiloride hydrochloride and 50 mg hydrochlorothiazide once daily, together with dietary advice on salt restriction. As noted above, this was compared to a multivitamin tablet that was used as a placebo. 

Park 2016 stated that 90 mL of isosorbide was used three times per day, but it is unclear what dose this corresponds to (the concentration is not stated). The authors also state that the dose of isosorbide could be reduced at the investigators' discretion, but it is not clear whether this occurred and, if so, in how many participants. All participants in this study also received 6 mg betahistine three times daily. 

###### Comparison 3: Antiviral versus no treatment/placebo

A single study considered the use of antivirals for Ménière's disease. Derebery 2004 randomised participants to either famciclovir (250 mg three times daily for 10 days, then twice daily for a further 80 days) or placebo. 

###### Comparison 4: Steroids versus no treatment/placebo

Finally, one study assessed the use of oral steroids (Morales‐Luckie 2005). Participants received steroids according to their weight (0.35 mg/kg/day oral prednisolone) or no treatment. All participants in this study received background treatment of diphenidol (25 mg/day), acetazolamide (250 mg every 48 hours) and a recommendation to follow a low‐sodium diet (< 1500 mg/day), as well as advice on reducing stress and consumption of alcohol, caffeine and nicotine. As noted above, it is possible that participants in this study had more frequent and severe symptoms than those in the other studies, as their attack frequency at baseline was approximately one attack per day, and they had self‐rated severe disability from their vertigo symptoms. 

##### Outcomes

###### 1. Improvement in vertigo

For this outcome we included dichotomous data ‐ assessed as the proportion of participants whose vertigo had 'improved' or 'not improved'. 

####### 1.1. Global score

Few studies reported the improvement of vertigo using a global score that considered the frequency, duration and intensity of vertigo attacks. 

Mira 2003 included an assessment of the improvement in "intensity score" for vertigo at three months. However, the scale used did not incorporate either the frequency of vertigo episodes or the duration of episodes, therefore we did not regard it as a true 'global score' of vertigo. 

A global score was not used to assess improvement in vertigo by the remaining studies.

####### 1.2. Frequency

Two studies that assessed improvement in vertigo frequency used the AAO‐HNS 1995 'control of vertigo' scale (Albu 2016; Morales‐Luckie 2005). The number of vertigo attacks in the interval after treatment is divided by the number of vertigo spells prior to treatment and multiplied by 100. The resulting number indicates the extent of ‘control of vertigo’ or CoV. The AAO‐HNS further divides the control of vertigo into classes, where class A (CoV = 0) represents a complete control of vertigo, class B (CoV 1% to 40%) represents a substantial control of vertigo, class C (41% to 80%) limited control, class D (81% to 120%) insignificant control and class E (> 120%) worse control (deterioration). When assessing any improvement in vertigo, we considered participants with a CoV of A, B or C to have experienced improvement, and those with a CoV of D or E to have not improved. For the sensitivity analysis of substantial improvement or complete resolution of vertigo we considered participants with a CoV of A or B to have substantial improvement/complete resolution and those with CoV C, D or E to have not had this degree of improvement. 

One study used an earlier version of this scale, from the AAOO 1972 guidelines (Ricci 1987). This considers both vertigo and hearing loss. In brief, participants are assigned to Class A (absence of dizzy spells and improvement in hearing), Class B (absence of dizzy spells and no change in hearing), Class C (absence of dizzy spells and worsening of hearing) or Class D (failure to control dizzy episodes). An improvement in frequency of vertigo was considered to be any participant with Class A, B or C control. However, it should be noted that this actually represents a complete resolution of vertigo episodes, not simply a reduction in frequency. Therefore, this was only included in our sensitivity analysis for this outcome.

The study Derebery 2004 used a different measure, and assessed the proportion of participants in each treatment arm who showed a 20% reduction in "disabling vertigo episodes" at three months. This should equate to approximately class C control of vertigo on the AAO‐HNS 1995 "control of vertigo" scale (although this scale considers all vertigo episodes, not just disabling episodes).

The study Khan 2011 used a patient questionnaire to assess this outcome, which considered both the number and severity of attacks. Duration of attacks was not included, therefore we did not consider this to be a global score of vertigo. A reduction in either the intensity of attacks or the frequency of attacks over the course of the study was considered to be an improvement. 

Improvement in vertigo frequency was not apparently assessed or reported by five studies (Adrion 2016; Mira 2003; Park 2016; Schmidt 1992).

###### 2. Change in vertigo

This outcome included data on the change in vertigo using a continuous numerical scale. 

####### 2.1. Global score

A single study assessed the change in vertigo using a global score (Schmidt 1992). An "imbalance scale" was used to assess vertigo, which included the intensity or severity of symptoms, the duration of symptoms and the frequency of attacks. 

A global score of vertigo change was not apparently assessed or reported by the remaining studies.

####### 2.2. Frequency

Adrion 2016 reported the mean attack rates per month (30 days) at nine months of follow‐up. The frequency of vertigo attacks at follow‐up was also assessed by Derebery 2004, Duphar B.V. 77.054/M, Mira 2003, Morales‐Luckie 2005 and Park 2016. 

Change in vertigo frequency was not reported by four studies (Albu 2016; Khan 2011; Ricci 1987; Schmidt 1992).

###### 3. Serious adverse events

This outcome included any event that caused death, was life‐threatening, required hospitalisation, resulted in disability or permanent damage, or in congenital abnormality. Serious adverse events were fully reported in only one study (Adrion 2016). Four studies did not appear to systematically collect data on serious adverse events, but did provide some description of other adverse events (which may suggest that no serious adverse events occurred), or stated that 'no adverse events occurred' (Albu 2016; Derebery 2004; Duphar B.V. 77.054/M; Mira 2003; Schmidt 1992). Four studies did not report at all on serious adverse events (Khan 2011; Morales‐Luckie 2005; Park 2016; Ricci 1987). 

###### 4. Disease‐specific health‐related quality of life

The Dizziness Handicap Inventory (DHI) was most commonly used to assess this outcome (Adrion 2016; Derebery 2004; Park 2016). One study reported the DHI score, but did not include a measure of the variance with the results, therefore we were unable to include it in any meta‐analysis (Mira 2003). The Functional Level Scale was also used to assess this outcome by two studies (Albu 2016; Morales‐Luckie 2005).

Three studies did not report this outcome (Khan 2011; Ricci 1987; Schmidt 1992).

###### 5. Hearing

Pure tone audiometry (PTA) was used to assess hearing status in five studies (Adrion 2016; Albu 2016; Derebery 2004; Park 2016; Schmidt 1992). 

- Adrion 2016 and Schmidt 1992 assessed hearing using PTA at four different frequencies (0.25 kHz, 0.5 kHz, 1 kHz and 2 kHz). Schmidt 1992 reported this as a pure tone average, whilst Adrion 2016 reported the hearing loss at each of the individual frequencies.
- Albu 2016, Derebery 2004 and Park 2016 used PTA with a four‐frequency average (0.5 kHz, 1 kHz, 2 kHz and 3 kHz).

Some studies assessed this outcome as "improvement" in hearing, rather than the absolute change in hearing using a continuous measure. Improvement was defined as a change of 10 dB in hearing threshold on the better hearing side by Khan 2011 (no details were provided on the frequencies used for PTA), and a change of ≥ 30 dB by Ricci 1987 (assessed as the pure tone average at 0.5 kHz, 1 kHz and 2 kHz). 

The studies by Morales‐Luckie 2005 and Duphar B.V. 77.054/M also assessed hearing using PTA, but the results were not fully reported, and no numerical data were available for analysis. The study Mira 2003 did not assess hearing.

###### 6. Tinnitus 

The Tinnitus Handicap Inventory was most frequently used to assess this outcome (Albu 2016; Derebery 2004; Park 2016). The mini Tinnitus Questionnaire was also used by one study (Adrion 2016).

Many studies assessed tinnitus using an unvalidated scale, or assessed features of tinnitus that were not selected as priorities for this review (such as the loudness or frequency of tinnitus). This included a visual analogue scale (Khan 2011), an assessment of the frequency of tinnitus (Morales‐Luckie 2005), or a part of a composite outcome (including aural fullness, nausea and vomiting; Mira 2003). Tinnitus was assessed by Ricci 1987, but a validated scale was not used and data are not presented separately for the two groups. Similarly, tinnitus loudness and the minimal masking level was assessed by Schmidt 1992, but there was no assessment of the impact of tinnitus on quality of life. These results have not been included in the review. 

###### 7. Other adverse effects

Adrion 2016 reported that data on adverse effects were systematically collected, and the authors provided us with data for the adverse effects of interest in this review (C. Adrion, personal communication). Duphar B.V. 77.054/M, Mira 2003 and Schmidt 1992 fully reported on a number of adverse effects of interest in the review.  Some studies did not appear to systematically collect data on adverse effects, but did provide some description of adverse events, or stated that no events occurred (Albu 2016; Derebery 2004; Morales‐Luckie 2005; Park 2016). Two studies did not report at all on adverse effects (Khan 2011; Ricci 1987).

#### Excluded studies

After assessing the full text, we excluded 62 articles from this review. The main reason for exclusion for each article is listed below. 

Twenty‐four studies were not randomised controlled trials (Albernaz 1970; Beckmann 1970; Beliakova 1971; Bosch 1970; Brookes 1982; Celestino 1970; Dowdy 1965; Elia 1970; Frew 1976; Greiner 1975; Guay 1970; Hausler 1989; Hommes 1972; Jongkees 1972; Klonowski 1972; Lazeanu 1968; Najwer 1973; Pialoux 1981; Popiel 1975; Przymanowski 1966; Reker 1983; Segers 1972; Wolfson 1967; Wouters 1983).

Eleven studies were excluded due to the inclusion of an inappropriate population (Duphar 108.005 80/M; Duphar H. 108.5009/M; Duphar H 108.906 NL; Duphar H 108 027 86 F/M; Canty 1981; Cohen 1972; Redon 2011), intervention (Albernaz 1968; Guyot 2008), or comparator (Huy 1992; Yamazaki 1988).

Fourteen studies could not be included because they were cross‐over trials from which data from the first phase could not be extracted (Klockhoff 1967; Oosterveld 1984; van Deelen 1986; Watanabe 1967; Wilmot 1976), or because the duration of follow‐up was less than three months, therefore insufficient (Beigh 2017; Burkin 1967; Elia 1965; Elia 1966; Liu 2020; Okamoto 1968; Salami 1984; Solvay  H. 1 08.035.92/F; Yu 2012).

One RCT was terminated after recruitment of only 11 participants and no outcome data were available (NCT01526408). 

Finally, we identified a number of review articles or commentaries that did not provide any primary outcome data. This included four narrative reviews (Celestino 1969; Conde 1965; Godlowski 1965; Richards 1971), and two commentaries on the included study Adrion 2016 (Ernst 2017; Helling 2017). We excluded six systematic reviews (Devantier 2020; Dimitriadis 2017; James 2003; James 2004; Murdin 2016; Van Esch 2022), but first checked their reference lists to ensure that any relevant studies had been included in our review.

Two articles are currently listed as awaiting classification (Beliakova 1971; Lazeanu 1968). These are both non‐English language papers, and we have been unable to obtain a full translation of the articles.

### Risk of bias in included studies

See Figure 3 for the risk of bias graph (our judgements about each risk of bias item presented as percentages across all included studies) and Figure 4 for the risk of bias summary (our judgements about each risk of bias item for each included study). All the studies included had some concerns regarding the risk of bias, with at least two domains being rated at unclear or high risk of bias. 

**3:**
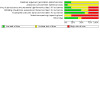
Risk of bias graph (our judgements about each risk of bias item presented as percentages across all included studies).

**4:**
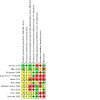
Risk of bias summary (our judgements about each risk of bias item for each included study).

#### Allocation

##### Random sequence generation

Two studies reported the use of a computer‐generated randomisation list and we considered them at low risk of bias (Adrion 2016; Albu 2016). The methods for sequence generation were not stated by the majority of studies, therefore we rated these as at unclear risk of bias (Derebery 2004; Duphar B.V. 77.054/M; Khan 2011; Mira 2003; Park 2016; Ricci 1987; Schmidt 1992). One study indicated that they used minimisation to allocate participants to groups (Morales‐Luckie 2005); however, no information is given on the methods used for this (including prognostic factors that were accounted for, and the statistical software used). We had concerns that this process was carried out by the investigator themselves, and therefore considered that it may not be equivalent to randomisation. We therefore also rated this study at unclear risk of bias. 

##### Allocation concealment

One study used a third party to carry out randomisation and allocation and we rated it at low risk of bias (Adrion 2016). The remaining studies did not provide any information on allocation concealment therefore we rated them at unclear risk of bias.

#### Blinding

##### Blinding of participants and personnel

Only two of the included studies reported blinding of both study participants and personnel (Adrion 2016; Albu 2016). Five studies involved the use of a placebo, therefore presumably blinded participants to their treatment allocation, but did not provide information on whether study personnel were also blinded to the group allocation (Derebery 2004; Duphar B.V. 77.054/M; Mira 2003; Ricci 1987; Schmidt 1992). We therefore rated these at unclear risk of performance bias. Three studies did not use a placebo in the comparator group, therefore it appears that study participants and personnel would have been aware of group allocation (Khan 2011; Morales‐Luckie 2005; Park 2016). Consequently, we rated them at high risk of performance bias. 

##### Blinding of outcome assessors

We considered this from the perspective of the primary outcomes (improvement in vertigo and change in vertigo). For a number of studies these outcomes were reported by the participants themselves, who were blinded to their allocated intervention (Adrion 2016; Derebery 2004; Duphar B.V. 77.054/M; Mira 2003; Schmidt 1992). Therefore, we considered these studies to be at low risk of bias. 

Outcomes were also reported by the blinded participants in Albu 2016. However, we noted that some adverse effects (including headache, nausea and diarrhoea) were only reported for those participants who received the intervention. As these symptoms were likely to have occurred in the control group as well (over the two‐year follow‐up) we had concerns that outcome assessors may have been made aware of the group allocation during the study. However, this may also simply be due to selective reporting, therefore we rated this domain as at unclear risk of bias. 

The description of the methods in Ricci 1987 was very brief. Although a placebo was stated to be used, the authors did not describe blinding at all in the article. Therefore, we were uncertain whether participants were truly blinded to their group allocation. It was also unclear whether the class of vertigo control would have been rated by participants themselves or study personnel, so we rated this domain as at unclear risk of bias. 

In three studies, outcomes were reported by participants who were aware of their group allocation, therefore we considered these to be at high risk of bias (Khan 2011; Morales‐Luckie 2005; Park 2016).

#### Incomplete outcome data

Some studies had full follow‐up or few dropouts, or the number of dropouts was fairly balanced across the intervention groups or were considered unlikely to impact the overall trial results. We rated these studies as at low risk of attrition bias (Albu 2016; Derebery 2004; Morales‐Luckie 2005; Park 2016; Ricci 1987; Schmidt 1992).

In three studies, a large number of participants dropped out over the course of the trial ‐ sufficient to impact on the overall results. We considered these to be at high risk of attrition bias (Adrion 2016; Duphar B.V. 77.054/M; Khan 2011). We note that Adrion 2016 accounted for missing outcome data using imputation during their analysis, which may reduce the impact of missing data to some extent. However, we considered that the quantity of missing data (almost 20% of study participants) would still be sufficient to introduce the potential for bias in the results.

One study did not clearly report the number of participants with Ménière's disease who dropped out of the trial (this study including a mixed population of people with Ménière's disease and benign paroxysmal positional vertigo (BPPV)) therefore we rated it at unclear risk of bias (Mira 2003).

#### Selective reporting

We rated all the included studies as being at either high or unclear risk of selective reporting. 

Where the protocol for the trial was unavailable, or had been retrospectively registered, we rated this domain as at unclear risk of bias, as we were unable to ascertain whether the trial had been full reported as pre‐specified (Albu 2016; Park 2016). 

Seven studies had no protocol available, but also had additional concerns over the potential for selective reporting. We rated these at high risk of bias. One study stated in the methods that follow‐up would occur at three months and six months, yet data were only reported at the three‐month time point (Derebery 2004). This may be due to the failure of efficacy at the three‐month time point, but we considered that there was a potential risk of reporting bias. Duphar B.V. 77.054/M assessed hearing with pure tone audiometry, but did not report these results fully, therefore they could not be included in a meta‐analysis. One further study had no registered protocol, and we also had concerns about the lack of description of adverse effects, as well as potential selective reporting of vertigo outcomes (Khan 2011). The study Mira 2003 fully reported vertigo outcome data for the intervention group, but did not report full details for the control group, therefore we were unable to include the results in the analysis. The study Morales‐Luckie 2005 reported different vertigo outcomes at different time points, therefore we considered this a risk for selective reporting bias. The study Ricci 1987 stated that adverse effects and biochemical tests would be assessed during the trial but did not report on these outcomes. The study Schmidt 1992 indicated in the methods that some outcomes would be assessed, but then failed to report the results. In addition, the analysis methods had to be changed during the course of the trial with post hoc decisions regarding data inputting. 

Adrion 2016 reported that their pre‐specified analysis methods were changed because of the significant amount of missing outcome data. In addition, the trial protocol stated that results would be recorded at nine months and 12 months, but data were only reported at the nine‐month follow‐up. Therefore, we considered that there was a potential risk of reporting bias. 

#### Other potential sources of bias

No additional concerns were noted for four studies, which we therefore rated as low risk of bias for the final domain of the risk of bias tool, which considers other potential sources of bias (Adrion 2016; Derebery 2004; Mira 2003; Park 2016).

We rated the study Morales‐Luckie 2005 at unclear risk of bias, because of concerns of differential follow‐up times between the two groups. 

We rated five studies at high risk of bias, predominantly because of concerns over the methods used to assess the primary outcomes. We had multiple concerns regarding the methods of outcome assessment used by Khan 2011. We also had multiple concerns regarding the trial Ricci 1987, due to a lack of detail on the inclusion criteria and diagnostic criteria used in the study, and concerns over the rating scales used for vertigo. We also had concerns over the validity of the rating scale used for vertigo in Duphar B.V. 77.054/M and Schmidt 1992. The study Albu 2016 used background treatments of intratympanic steroids in both groups of participants. Additional injections should have been offered to participants if their vertigo symptoms had not resolved. However, this does not appear to have been rigorously used in participants in the control group, as a number of participants did not have complete/substantial vertigo control at the end of the trial, but had not had the maximum number of intratympanic steroid injections. Therefore, there may be a risk of deviation from the trial protocol in this study, leading to performance bias. 

### Effects of interventions

See: Table 1; Table 2

#### 1. Betahistine versus no treatment/placebo

Seven studies considered this comparison (Adrion 2016; Albu 2016; Duphar B.V. 77.054/M; Khan 2011; Mira 2003; Ricci 1987; Schmidt 1992). As described above, the dose of betahistine varied considerably across the studies. One included study was a three‐arm trial comparing high‐dose and low‐dose betahistine with placebo (Adrion 2016). For the purposes of this review we have pooled these different doses for analysis. 

##### 1.1. Improvement in vertigo 

For this outcome we included any data that were reported as a dichotomous (binary) outcome, i.e. classifying participants as having improved or not improved. 

###### 1.1.1. Global score

No studies considered improvement in vertigo using a global score, which included frequency, duration and severity of vertigo. 

###### 1.1.2. Vertigo frequency

####### 1.1.2.1. 3 to < 6 months

No study reported at this time point. 

####### 1.1.2.2. 6 to ≤ 12 months

A single study reported at this time point (Khan 2011), but the evidence was of very low certainty. The risk ratio for any improvement in vertigo was 1.50 (95% confidence interval (CI) 0.98 to 2.29; 1 study; 70 participants; Analysis 1.1; very low‐certainty evidence).

As described above, we also considered whether changing our outcome to "complete or substantial improvement in vertigo" would impact the effect size. However, we also had a single study for this analysis and, although the effect size was larger, the certainty of the evidence was still very low (Peto odds ratio (OR) 13.08, 95% CI 1.01 to 170.31; 1 study; 10 participants; Analysis 1.2; very low‐certainty evidence). 

####### 1.1.2.3. > 12 months

The evidence was also of very low certainty at a longer duration of follow‐up (RR 1.11, 95% CI 0.93 to 1.32; 1 study; 62 participants; Analysis 1.1; very low‐certainty evidence).

Again, we considered whether changing the outcome to "complete or substantial improvement" would make a difference to the estimated effect. As above, we had a single study for this analysis and the evidence was still very low‐certainty (RR 1.37, 95% CI 1.04 to 1.81; 1 study; 62 participants; Analysis 1.3; very low‐certainty evidence).

##### 1.2. Change in vertigo

For this outcome we included any continuous data ‐ where the change in vertigo was measured on a continuous scale (such as with a numerical scoring system, or the actual number of vertigo episodes experienced in a given time period). 

###### 1.2.1. Global score

One study considered change in vertigo using a global score, which included frequency, duration and severity of vertigo (Schmidt 1992). The authors used a mean monthly imbalance score. The potential range of scores was from zero (no vertigo attacks per month) to approximately 63 (a severe vertigo attack every day of the week), with higher scores representing worse vertigo. The results are reported as the geometric mean score at 16 weeks of follow‐up. 

####### 1.2.1.1. 3 to < 6 months

The difference in the geometric mean score for those receiving betahistine was 0.7 points higher (mean difference (MD) 0.70, 95% CI ‐6.67 to 8.07; scale 0 to 63; 1 study; 34 participants; Analysis 1.4; very low‐certainty evidence). We considered this unlikely to be a meaningful difference to people with Ménière's disease. 

####### 1.2.1.2. > 6 to ≤ 12 months and > 12 months

No data were reported for these time points. 

###### 1.2.2. Vertigo frequency

####### 1.2.2.1. 3 to < 6 months

Two studies reported at this time point (Duphar B.V. 77.054/M; Mira 2003). Data used in this analysis required some estimation. One study did not report a standard deviation (which was therefore estimated using the methods of Wan 2014), and the data required re‐scaling from the number of episodes in six weeks, to the number in one month (taken as four weeks; Duphar B.V. 77.054/M). The second study reported the change from baseline in the control group as a percentage, therefore we estimated this using the baseline score and percentage change. We also estimated the standard deviation, using a conservative estimate from the baseline standard deviation of the control group (Mira 2003). There is consequently great uncertainty in this analysis. Those receiving betahistine had a reduction in the frequency of vertigo attacks of 1.90 attacks per month, but the evidence was very uncertain (95% CI ‐3.05 to ‐0.74; 2 studies; 117 participants; I^2^ = 0%; Analysis 1.5; very low‐certainty evidence). 

####### 1.2.2.2. 6 to ≤ 12 months

One study reported at this time point (Adrion 2016). The mean difference in the frequency of vertigo for those receiving betahistine was an increase of 0.63 attacks per 30 days, but this evidence was also very uncertain (95% CI ‐4.07 to 5.33; 1 study; 214 participants; Analysis 1.5; very low‐certainty evidence).

####### 1.2.2.3. > 12 months

No data were reported for this time point.

##### 1.3. Serious adverse events

A single study reported on serious adverse events and the evidence was very uncertain (RR 1.20, 95% CI 0.63 to 2.29; 1 study; 220 participants; Analysis 1.6; very low‐certainty evidence).  

##### 1.4. Disease‐specific health‐related quality of life

Three studies considered this outcome in some way.

###### 1.4.1. 3 to < 6 months

The study Mira 2003 did assess the Dizziness Handicap Inventory (DHI) at three months of follow‐up, but data were only reported as the percentage change in score for each group, without an estimate of variance, and were reported for the entire cohort (those with benign paroxysmal positional vertigo (BPPV) and Ménière's disease) therefore we were unable to use these results in this review. 

###### 1.4.2. 6 to ≤ 12 months

One study reported this outcome (Adrion 2016). The authors used the DHI but assessed this as the mean score per question (to account for missing outcome data). Therefore, the range of results is from 0 to 4, with higher scores representing worse quality of life. The mean difference for those receiving betahistine was 0.06 points higher than those receiving placebo (95% CI ‐0.17 to 0.29; 1 study; 170 participants; low‐certainty evidence; Analysis 1.7). This would equate to a change of approximately 1.5 points on the full DHI score (range 0 to 100, higher scores represent worse quality of life, minimally important difference (MID) = in the range of 11 to 18 points; Jacobsen 1990; Tamber 2009), with a 95% confidence interval from ‐4.25 to 7.25.   

###### 1.4.3. > 12 months

One study described assessing quality of life using the Functional Level Scale of the AAO‐HNS 1995 guideline. For the purposes of this review we have included those who rated their Functional Level Score as 1 or 2 at follow‐up. The RR for an Functional Level Score score of 1 or 2 was 1.34 for the betahistine group (95% CI 1.07 to 1.69; 1 study; 62 participants; very low‐certainty evidence; Analysis 1.8). 

##### 1.5. Change in hearing

Some studies reported hearing data as continuous (i.e. the change in pure tone average), whilst others reported this as a dichotomous score (the number of participants in whom hearing improved by a certain amount). We report these data separately in the review. 

###### 1.5.1. 3 to < 6 months

The study Schmidt 1992 reported the average air conduction threshold at 0.25 kHz, 0.5 kHz, 1 kHz and 2 kHz at four months. The mean difference in hearing threshold was 10.10 dB HL higher (i.e. worse) for those who received betahistine, as compared to those receiving placebo (95% CI ‐1.13 to 21.33; 1 study; 35 participants; low‐certainty evidence; Analysis 1.9). This may be an important deterioration in hearing, but it should be noted that the evidence is of low certainty and the confidence interval also includes the possibility of a trivial change. 

The study Duphar B.V. 77.054/M included an assessment of hearing using pure tone audiometry, but results were only reported narratively. The authors stated: "The pure tone audiogram changed in 4 out of 46 patients. In one patient pure tone audiogram worsened after betahistine treatment and in two patients after placebo treatment. One patient had a better pure tone audiogram after placebo treatment". We were not able to incorporate these data in a meta‐analysis, and the authors did not provide any description of the thresholds used to determine a 'better' or 'worse' audiogram. 

###### 1.5.2. 6 to ≤ 12 months

Adrion 2016 also used pure tone audiometry at four frequencies to assess hearing (0.25 kHz, 0.5 kHz, 1 kHz and 2 kHz). The data were reported separately for each frequency in the article, therefore we have pooled these to create a summary measure for analysis. Overall, the mean difference in hearing threshold for those receiving betahistine was a deterioration in hearing of 2.64 dB HL (95% CI ‐1.66 to 6.94; 1 study; 113 participants; very low‐certainty evidence; Analysis 1.9). This analysis should be interpreted with caution as the methods that have been used to estimate the four‐tone average may not perfectly recreate the original data.

Two studies reported hearing as dichotomous data, describing the number of participants in whom an improvement of ≥ 10 dB on the "better side" was achieved (Khan 2011), or an improvement of ≥ 30 dB was achieved (Ricci 1987). We are uncertain about these results. It is unclear why Khan 2011 used the "better hearing side" for their analysis. The authors do not state that participants with bilateral disease were exclusively recruited to the study, therefore it is possible that participants had unilateral disease, and therefore the "better hearing side" is likely to be the ear that is not affected by Ménière's disease. Similarly, the threshold for improvement in Ricci 1987 seems very high, and may therefore underestimate the number of people in whom some improvement in hearing was seen. Nonetheless, the Peto odds ratio for improvement was 3.14 in those receiving betahistine (95% CI 1.28 to 7.66; 2 studies; 82 participants; I^2^ = 0%; very low‐certainty evidence; Analysis 1.10).

###### 1.5.3. > 12 months

The study Albu 2016 assessed the hearing threshold using the pure tone average of four frequencies (0.5 kHz, 1 kHz, 2 kHz and 3 kHz) and found a mean difference of 1.40 dB HL in those receiving betahistine (95% CI ‐7.10 to 9.90; 1 study; 62 participants; very low‐certainty evidence; Analysis 1.9). 

##### 1.6. Change in tinnitus

Only two studies reported change in tinnitus using a validated scale that assessed the impact of tinnitus on quality of life. 

###### 1.6.1. 3 to < 6 months

No studies reported at this time point. 

###### 1.6.2. 6 to ≤ 12 months

Adrion 2016 assessed tinnitus using the mini Tinnitus Questionnaire. The data were originally reported with the mean score per question (using a scale of 0 to 2), however we have transformed these data back to the original scale (0 to 24) for analysis and presentation. The mean difference was ‐0.06 for those receiving betahistine (95% CI ‐1.52 to 1.39; scale 0 to 24; 1 study; 168 participants; low‐certainty evidence; Analysis 1.11). 

###### 1.6.3. > 12 months

Albu 2016 used the Tinnitus Handicap Inventory. Again, the mean difference between the groups was probably trivial (MD 0.9 points higher, 95% CI ‐5.55 to 7.35; scale 0 to 100; 1 study; 62 participants; very low‐certainty evidence; Analysis 1.11). 

##### 1.7. Other adverse effects

Only four studies provide numeric data on the number of participants affected by the adverse effects of interest in this review (Adrion 2016; Duphar B.V. 77.054/M; Mira 2003; Schmidt 1992). 

The study Albu 2016 only reported other adverse effects in those receiving betahistine, and did not provide any data for the placebo group. They reported five cases of headache and eight cases of diarrhoea in the 30 participants who received betahistine. However, these results may be subject to selective reporting bias (it seems unlikely that no participants receiving placebo suffered a headache during the two‐year follow‐up period). 

Ricci 1987 indicated that adverse effects would be assessed in the methods of their study, but did not report on this outcome. It is not clear whether this is because no events occurred, or because they were not reported. Khan 2011 did not provide any information on adverse effects.

###### 1.7.1. Headache

Four studies considered the risk of headache with betahistine (Adrion 2016; Duphar B.V. 77.054/M; Mira 2003; Schmidt 1992). The Peto OR for those receiving betahistine was 1.16. However, there was considerable inconsistency in this analysis, with two studies showing a trivial difference between the groups, one study showing an increased risk for those receiving betahistine, and one showing a reduction in risk. Therefore, we are very uncertain about the result (Peto OR 1.16, 95% CI 0.69 to 1.95; 4 studies; 374 participants; I^2^ = 50%; very low‐certainty evidence; Analysis 1.12). 

###### 1.7.2. Gastrointestinal disturbance

Again, Adrion 2016, Duphar B.V. 77.054/M, Mira 2003 and Schmidt 1992 reported on the occurrence of gastrointestinal disturbance. There was also a great deal of inconsistency in this analysis, therefore we are very uncertain about the results (Peto OR 1.08, 95% CI 0.65 to 1.78; 4 studies; 372 participants; I^2^ = 42%; very low‐certainty evidence; Analysis 1.12). 

###### 1.7.3. Sleep disturbance

Adrion 2016 and Schmidt 1992 reported on the occurrence of sleep disturbance, and found a RR of 1.43 for those using betahistine, but the confidence intervals were very wide (95% CI 0.47 to 4.38; 2 studies; 255 participants; very low‐certainty evidence; Analysis 1.13). 

###### 1.7.4. Dry mouth

Adrion 2016 and Mira 2003 reported on this outcome. The Peto OR for those receiving betahistine was 0.30 (95% CI 0.05 to 1.95; 2 studies; 301 participants; low‐certainty evidence; Analysis 1.12). 

###### 1.7.5. Steroid‐related side effects

This outcome was not reported by any of the studies, but is only of relevance to the comparison of corticosteroids with no treatment/placebo (see below). 

#### 2. Diuretics versus no treatment/placebo

Two studies considered this comparison (Khan 2011; Park 2016). We considered both to be at high risk of performance and detection bias due to a lack of blinding of participants, study personnel and outcome assessors. Khan 2011 used a combination of amiloride hydrochloride (5 mg) and hydrochlorothiazide (50 mg) once a day, plus advice on salt restriction. Park 2016 used 90 mL of isosorbide per day, but the concentration (and total dose) was not stated. Due to the concerns over the risk of bias in these studies, together with the relatively small sample sizes, we rated all the evidence for this comparison as very low‐certainty.

##### 2.1. Improvement in vertigo

Park 2016 did not report on this outcome. 

###### 2.1.1. Global score

Neither study considered improvement in vertigo using a global score, which included frequency, duration and severity of vertigo. 

###### 2.1.2. Vertigo frequency

####### 2.1.2.1. 3 to < 6 months

Neither study reported at this time point. 

####### 2.1.2.2. 6 to ≤ 12 months

Khan 2011 reported an increase in the number of participants who self‐reported an improvement in the frequency or severity of their vertigo at 12 months follow‐up when using diuretics (77% of those receiving diuretics improved, 46% of those receiving placebo improved, RR 1.69, 95% CI 1.13 to 2.53; 1 study; 70 participants; very low‐certainty evidence; Analysis 2.1). However, we assessed the evidence as very low‐certainty, due to concerns over the risk of bias with this study, the small sample size and because the outcome considered both frequency and severity of vertigo (i.e. some participants may have experienced a reduction in severity of vertigo, but the frequency may be unchanged). 

####### 2.1.2.3. > 12 months

Neither study reported at this time point. 

##### 2.2. Change in vertigo 

Khan 2011 did not report on this outcome. 

###### 2.2.1. Global score

Neither study considered improvement in vertigo using a global score, which included frequency, duration and severity of vertigo. 

###### 2.2.2. Vertigo frequency

####### 2.2.2.1. 3 to < 6 months

Park 2016 reported on the number of vertigo episodes during a four‐week period, after three months of follow‐up. The number of episodes was reduced in those who received isosorbide, with a mean difference of ‐2.44 episodes every four weeks, but the confidence interval includes the possibility of no effect, or a trivial effect, and the evidence is very uncertain (MD ‐2.44, 95% CI ‐4.98 to 0.10; 1 study; 220 participants; very low‐certainty evidence; Analysis 2.2). 

####### 2.2.2.2. 6 to ≤ 12 months

Neither study reported at this time point. 

####### 2.2.2.3. >12 months

Neither study reported at this time point. 

##### 2.3. Serious adverse events

Neither study assessed or reported this outcome. 

##### 2.4. Disease‐specific health‐related quality of life

Khan 2011 did not report on this outcome. 

###### 2.4.1. 3 to < 6 months

Park 2016 reported on quality of life using the Korean version of the DHI at three months of follow‐up. The mean difference was 2.94 points higher (worse) in those receiving isosorbide (95% CI ‐3.86 to 9.74; 1 study; 220 participants; very low‐certainty evidence; Analysis 2.3). However, this may be insignificant when the DHI has a minimally important difference in the range of 11 to 18 points (Jacobsen 1990; Tamber 2009).

###### 2.4.2. 6 to ≤ 12 months

Neither study reported at this time point. 

###### 2.4.3. > 12 months

Neither study reported at this time point. 

##### 2.5. Change in hearing

Both studies assessed hearing in some way. Park 2016 assessed the change in hearing threshold using pure tone audiometry at four frequencies. As described above, Khan 2011 considered the number of participants who achieved an improvement in hearing of at least 10 dB on the "better hearing side", but it is unclear why the "better" side was assessed. 

###### 2.5.1. 3 to < 6 months

Park 2016 reported a mean difference of ‐1.43 dB HL for those who received diuretics, which is probably a trivial difference between the groups (95% CI ‐3.88 to 1.02; 1 study; 220 participants; very low‐certainty evidence; Analysis 2.4). 

###### 2.5.2. 6 to ≤ 12 months

Khan 2011 reported an increase in the number of participants who achieved an improvement in hearing of ≥ 10 dB when taking diuretics, but the evidence was very uncertain (RR 1.77, 95% CI 1.07 to 2.91; 1 study; 72 participants; very low‐certainty evidence; Analysis 2.5). 

###### 2.5.3. > 12 months

Neither study reported at this time point. 

##### 2.6. Change in tinnitus

Khan 2011 did not report on this outcome. 

###### 2.6.1. 3 to < 6 months

Park 2016 reported on tinnitus using the Korean version of the THI at three months of follow‐up. The mean difference was 1.89 points higher (worse) in those receiving isosorbide, but this is insignificant as the THI has a minimally important difference of 6 to 7 points (95% CI ‐4.96 to 8.74; 1 study; 220 participants; very low‐certainty evidence; Analysis 2.6). 

###### 2.6.2. 6 to ≤ 12 months

Neither study reported at this time point. 

###### 2.6.3. > 12 months

Neither study reported at this time point. 

##### 2.7. Other adverse events

Khan 2011 did not assess or report any adverse effects. Park 2016 provided a narrative summary, which stated that eight participants (out of 110) in the diuretic group and seven participants (out of 110) in the control group experienced "mild to moderate drug‐related adverse reactions such as headache, indigestion, diarrhoea, nausea, sweating, insomnia, etc." (quote translated from the original Korean). No details were provided on the specific number of each adverse effect in the two groups. 

#### 3. Antiviral versus no treatment/placebo

A single study addressed this comparison, using 250 mg famciclovir three times daily for 10 days, followed by 250 mg twice daily for a further 80 days (Derebery 2004). Follow‐up was conducted at three months (90 days). This study only included 23 participants, therefore the results obtained were extremely imprecise with wide confidence intervals, and all the evidence for this comparison is of very low‐certainty. 

##### 3.1. Improvement in vertigo

###### 3.1.1. Global score

Derebery 2004 did not consider improvement in vertigo using a global score.

###### 3.1.2. Vertigo frequency

####### 3.1.2.1. 3 to < 6 months

At three months, Derebery 2004 reported an increase in the number of participants who achieved an improvement in vertigo frequency (defined as a reduction in the number of episodes by at least 20% as compared to their baseline measurement). However, the confidence intervals were very wide, and the very small study size led to great imprecision in the results, therefore we are very uncertain of the evidence (RR 1.38, 95% CI 0.28 to 6.75; 1 study; 23 participants; very low‐certainty evidence; Analysis 3.1). 

####### 3.1.2.2. 6 to ≤ 12 months and > 12 months

The study did not report at these time points. 

##### 3.2. Change in vertigo 

###### 3.2.1. Global score

Derebery 2004 did not consider change in vertigo using a global score.

###### 3.2.2. Vertigo frequency

####### 3.2.2.1. 3 to < 6 months

At three months, the frequency of dizzy episodes per week was slightly higher in those receiving antivirals, although it was likely to be a trivial difference, and the confidence intervals were very wide (MD 0.1 episodes per week higher, 95% CI ‐1.03 to 1.23; 1 study; 23 participants; very low‐certainty evidence; Analysis 3.2). 

####### 3.2.2.2. 6 to ≤ 12 months and > 12 months

The study did not report at these time points. 

##### 3.3. Serious adverse events

These were not reported by Derebery 2004. 

##### 3.4. Disease‐specific health‐related quality of life

###### 3.4.1. 3 to < 6 months

The DHI was used to assess quality of life, and the score was found to be slightly higher in those receiving antivirals at three months of follow‐up, but again the confidence intervals were too wide to draw any conclusions (MD 7.4 points, 95% CI ‐15.78 to 30.58; 1 study; 23 participants; very low‐certainty evidence; Analysis 3.3). 

###### 3.4.2. 6 to ≤ 12 months and > 12 months

The study did not report at these time points. 

##### 3.5. Change in hearing

Hearing was assessed using the average of four frequencies with pure tone audiometry. 

###### 3.5.1. 3 to < 6 months

The mean difference in hearing level was 4.3 dB higher (worse) for those receiving antivirals, although this would probably be a trivial difference, and the confidence intervals were very wide (95% CI ‐13.94 to 22.54; 1 study; 16 participants; very low‐certainty evidence; Analysis 3.4). 

###### 3.5.2. 6 to ≤ 12 months and > 12 months

The study did not report at these time points. 

##### 3.6. Change in tinnitus

Derebery 2004 did not report on the change in tinnitus.

##### 3.7. Other adverse events

These were not reported by Derebery 2004. 

#### 4. Corticosteroids versus no treatment/placebo

A single study addressed this comparison. Morales‐Luckie 2005 was a very small study of 16 participants, which compared the use of oral prednisolone (according to the participant's weight: 0.35 mg/kg/day) for 18 weeks to no intervention. All participants in this study also received maintenance treatment of diphenidol, acetazolamide and a low‐sodium diet. Although the intervention was given for only 18 weeks (followed by a tapering dose to stop the steroids), follow‐up was conducted off‐treatment, at just over 12 months of treatment. Most of the results available are at this time point. Given the small size of the study, the results are very imprecise with wide confidence intervals, therefore all the evidence for this comparison is very uncertain. 

##### 4.1. Improvement in vertigo

###### 4.1.1. Global score

Morales‐Luckie 2005 did not consider improvement in vertigo using a global score.

###### 4.1.2. Vertigo frequency

####### 4.1.2.1. 3 to < 6 months and 6 to ≤ 12 months

The study did not report at these time points. 

####### 4.1.2.2. > 12 months

Morales‐Luckie 2005 used the AAO‐HNS 1995 control of vertigo scale to assess improvement in vertigo frequency. There was no difference in the number of participants who achieved any improvement in vertigo when taking steroids (AAO‐HNS 1995 Class A, B or C, RR 1.00, 95% CI 0.80 to 1.25; 1 study; 16 participants; very low‐certainty evidence; Analysis 4.1). The evidence was also very uncertain when we considered those who experienced a substantial improvement or complete resolution in their vertigo symptoms (AAO‐HNS 1995 Class A or B) although the odds ratio did favour corticosteroids (Peto OR 42.52, 95% CI 6.37 to 283.65; 1 study; 16 participants; very low‐certainty evidence; Analysis 4.2).  

##### 4.2. Change in vertigo 

###### 4.2.1. Global score

Morales‐Luckie 2005 did not consider change in vertigo using a global score.

###### 4.2.2. Vertigo frequency

####### 4.2.2.1. 3 to < 6 months

This was reported as the number of vertigo episodes per day, and was reported at 18 weeks of follow‐up (whilst participants were still receiving corticosteroids). It should be noted that participants in this study appeared to have a very high frequency of vertigo attacks, with an average of approximately one attack per day at baseline. The mean difference in vertigo episodes at follow‐up was 0.44 fewer per day in those who received corticosteroids (95% CI ‐0.7 to ‐0.18; 1 study; 16 participants; very low‐certainty evidence; Analysis 4.3). 

####### 4.2.2.2. 6 to ≤ 12 months and > 12 months

The study did not report this outcome at these time points. 

##### 4.3. Serious adverse events

These were not reported by Morales‐Luckie 2005. 

##### 4.4. Disease‐specific health‐related quality of life

###### 4.4.1. 3 to < 6 months and 6 to ≤ 12 months

The study did not report at these time points. 

###### 4.4.2. > 12 months

The AAO‐HNS 1995 Functional Level Scale was used to assess quality of life. The authors reported on the number of participants in whom the Functional Level Scale score improved over the course of the study. This was found to be greater for those receiving corticosteroids than those who received no intervention, but the evidence is very uncertain due to the small sample size, wide confidence interval and risk of bias associated with this study (Peto OR 28.03, 95% CI 4.14 to 189.82; 1 study; 16 participants; very low‐certainty evidence; Analysis 4.4). 

##### 4.5. Change in hearing

Hearing was assessed but not fully reported. The authors only state that "the statistical analysis did not reveal significant differences between the groups in any frequency category".  

##### 4.6. Change in tinnitus

Tinnitus was not assessed using a validated questionnaire that considered the impact of tinnitus on quality of life. Instead, the authors reported only on the frequency of tinnitus, which was not an outcome of interest in this review. 

##### 4.7. Other adverse events

Adverse events were not fully reported in the article. No data were available on the occurrence of headache, gastrointestinal disturbance, sleep disturbance or dry mouth. The authors did report the occurrence of one "steroid‐related side effect", which was the development of ankle oedema in one participant who received corticosteroids (Peto OR 7.39; 95% CI 0.15 to 372.38; 1 study; 16 participants; very low‐certainty evidence; Analysis 4.5). 

## Discussion

### Summary of main results

#### Betahistine versus placebo/no treatment

Seven studies provided some data for this comparison (Adrion 2016; Albu 2016; Duphar B.V. 77.054/M; Khan 2011; Mira 2003; Ricci 1987; Schmidt 1992). All the data identified regarding improvement in vertigo and change in vertigo was of very low certainty, therefore we cannot be sure whether betahistine has an important effect on vertigo. Few studies reported either serious adverse events or other adverse effects in full, and again the evidence was of low or very low certainty, so we cannot draw any firm conclusions about the risk of side effects with betahistine. The evidence for hearing was rather mixed, with some low‐certainty evidence for short‐term follow‐up (3 to < 6 months) suggesting a slight worsening of hearing in those who received betahistine (Schmidt 1992), although the confidence interval was wide, and included the possibility of a trivial difference between the groups. Data from later follow‐up was of very low certainty, but tended to show a trivial difference between the groups, or slightly favour betahistine. Similarly, the two studies that assessed tinnitus resulted in low‐ or very low‐certainty evidence, and there appeared to be a trivial difference between those who did or did not receive betahistine (Adrion 2016; Albu 2016). Finally, two studies reported data on disease‐related quality of life. One provided low‐certainty evidence indicating that betahistine may result in a trivial difference in quality of life at 6 to ≤ 12 months follow‐up (Adrion 2016). The second provided very low‐certainty evidence, but did suggest that betahistine may improve quality of life (Albu 2016). 

#### Diuretics versus placebo/no treatment

We identified some evidence on the improvement in vertigo, the change in vertigo and disease‐specific health‐related quality of life from two studies that assessed diuretics (Khan 2011; Park 2016), but we considered this all to be very low‐certainty evidence. We also considered evidence on the change in hearing and tinnitus to be very low‐certainty. Neither study considered serious adverse events, so we have no information for this outcome. One study provided a brief summary of mild or moderate adverse effects, but did not provide information on the specific number and type of complications in each group, so we are also very uncertain about the potential for other adverse effects. 

#### Antivirals versus placebo/no treatment

We only identified one small study of 24 participants that used antivirals (Derebery 2004). However, the evidence for all the outcomes assessed by this study was very low‐certainty. This included improvement in vertigo frequency, change in vertigo frequency, disease‐specific health‐related quality of life and change in hearing at three months of follow‐up. This study did not consider serious adverse events, or other potential adverse effects of treatment; nor did it assess the change in tinnitus, so we have no data for these outcomes. 

#### Corticosteroids versus placebo/no treatment

Finally, we identified one small study of 16 participants that considered the use of corticosteroids for Ménière's disease (Morales‐Luckie 2005). Again, the evidence for this comparison was all of very low certainty, and the outcomes considered were improvement in vertigo frequency, change in vertigo frequency and disease‐specific health‐related quality of life. No data were available on serious adverse events, change in hearing or tinnitus, or most of our pre‐specified adverse effects of interest. The authors did report a single occurrence of a steroid‐related side effect (ankle oedema) but, again, the evidence for this outcome was of very low certainty.

### Overall completeness and applicability of evidence

There is a paucity of evidence about all of these interventions, despite some of them being in common use for Ménière’s disease. All the evidence we found was of very low or low certainty, showing that we are unsure of the effects of the interventions, and future research may change the effect estimates a great deal. Evidence for any benefit is lacking, and evidence on potential harms of the interventions is also sparse. 

We were unable to carry out many meta‐analyses for this review. This was due to a number of issues. Firstly, we identified few studies for inclusion in each of the comparisons of interest. The maximum number of included studies for any comparison was six (betahistine), but the remaining comparisons had only one or two studies included. In addition, where studies did address the same comparison, there were often differences in the actual outcomes assessed in the study, or the time points for follow‐up. Therefore, we were unable to pool the data to achieve a more precise estimate of any effect. Finally, study authors often used different ways of measuring the same outcome, which prevented data from being combined. For example, vertigo was assessed with either a global score, or a frequency score, which could not be combined, or hearing was assessed using a continuous scale or as an improvement above a certain threshold. 

Certain outcomes were only assessed by some included studies. Many studies did not assess the impact of the disease on quality of life or tinnitus at all. Potential adverse effects of the interventions were also often poorly reported or simply not assessed. Although we did not identify robust evidence on adverse effects in this review, many of these interventions have well‐recognised ‐ and potentially serious ‐ side effects. These include the risk of acute kidney injury and electrolyte imbalance with diuretics (Sica 2007; Tominey 2021), and widespread systemic effects from oral corticosteroids (Buchman 2001). The studies included in this review recruited only a relatively small number of participants, and most followed up participants for a short time. The ability to detect adverse effects in this review is therefore very limited. More information is needed on the actual risk of complications when these interventions are used in the treatment of Ménière’s disease.

We noted that unvalidated rating scales were commonly used in the studies included, particularly when looking at the global impact of treatments for vertigo. When such scales are used, it is difficult to know if they are accurately assessing the outcome, and also what size of change on this scale represents a meaningful difference in the outcome (the minimally important difference). 

Finally, studies often failed to report clearly what treatments participants received before joining the study, what maintenance treatment they continued on during the study, and whether they received any additional treatments over the course of the study. The impact of these additional treatments may be considerable, particularly for those studies with longer‐term follow‐up. Without knowing the background details of study participants (for example, the duration of their Ménière's disease, or what treatments they have tried in the past) it is difficult to identify the groups of people who may benefit from these treatments. 

In accordance with the protocol for this review, we specifically included studies that compared one intervention with placebo or no treatment. Therefore, studies that compared different types of active intervention (for example, betahistine compared to diuretics) were excluded from the review. We considered that this was important ‐ until it is clear that one intervention has a specific, beneficial effect on symptoms of Ménière's disease, there is no 'gold standard' treatment with which to compare other interventions. 

### Quality of the evidence

We used the GRADE approach to assess the certainty of the evidence in this review. The evidence identified was all low‐ or very low‐certainty, meaning that we are uncertain about the actual effect of these interventions for all of our outcomes. This is perhaps surprising, given that some interventions included in this review are in widespread use as first‐line treatments for Ménière's disease (for example, betahistine and diuretics). 

The main issues that affected the certainty of the evidence were the domains of study limitations and imprecision. The different domains addressed by GRADE are considered in more detail below.

#### Study limitations/risk of bias

All the studies included in this review had at least some concerns regarding the potential for bias in the study design, conduct or reporting. Several studies did not mask participants, study personnel or outcome assessors to the interventions used in each group, which led to a high risk of performance bias or detection bias (Khan 2011; Morales‐Luckie 2005; Park 2016). There were concerns regarding the appropriate use of methods for randomisation and allocation concealment for the majority of included studies, although we acknowledge that this may be in part due to poor reporting, rather than the actual conduct of the studies. There was the potential for attrition bias in some studies, due to considerable loss to follow‐up over the course of the study, or missing outcome data, which has the potential to bias the effect estimates (even if efforts are made to account for this in the analysis). Finally, we had concerns over selective reporting for many of the studies included in this review. Several studies planned to assess outcomes at multiple time points, but only provided follow‐up data for a single time point in their publications. Others pre‐specified how outcomes would be assessed and reported, but then deviated from this in the final publication. 

#### Inconsistency

Few meta‐analyses were conducted in the course of this review, therefore inconsistency did not usually impact on the certainty of the evidence. For the majority of outcomes, a single study was included in the analysis. Consequently, inconsistency between studies was not of relevance, and the certainty of the evidence was unaffected by this domain.

#### Indirectness

This was not a major concern for most of the outcomes. We did rate down the certainty of the evidence if included studies had used concomitant medication in both arms of the study. An example would be the study Albu 2016, where participants all received intratympanic corticosteroids, and then were randomised to either betahistine or placebo. We considered that the background therapy may have an impact on the effect estimates. The efficacy of betahistine in the study may be greater than usual (if the two treatments work together, synergistically) or the betahistine effect may be minimised (if the intratympanic therapy also caused an improvement in symptoms for the control group). We also rated down for indirectness if the majority of evidence for an outcome had come from studies where the population was not clearly defined (for example, Ricci 1987).

#### Imprecision

Almost all the included studies are very small and, as discussed above, we were unable to carry out very much meta‐analysis. Therefore, the total sample size for each of our outcomes of interest was small, and reduced the certainty of the evidence. For some outcomes the resulting confidence intervals for the effect size were also extremely wide ‐ meaning that there was uncertainty over whether the intervention was beneficial or harmful. This further impacted on the certainty of the evidence. 

The GRADE approach involves rating down the certainty of the evidence if the threshold for an *important* difference is crossed. 

For each analysis result, the width of the confidence interval is compared to the threshold for an important difference (details of how these thresholds were selected are described in the Methods section). If the confidence interval crosses this threshold ‐ and includes both the potential for an important benefit and the potential for a trivial effect, then the certainty of the evidence would be reduced by one level. If the confidence interval includes the possibility of *both* an important benefit and an important harm then the certainty would be reduced further. Therefore, it is important to agree on thresholds for this rating, i.e. where is the threshold, or cut‐point, between a trivial difference and a small, but important benefit or harm for each outcome? This question is difficult to answer, and requires input from people with balance disorders. As part of this review process, one of the author team (KW) joined some discussion groups for people with balance disorders, to try and obtain their views on quantifying an important and meaningful difference in treatment outcomes. However, the main theme that emerged from these discussions was that people were unable to give a specific threshold for each outcome. Instead, individuals tended to weigh up a variety of different factors when determining this threshold. The invasiveness and burden of taking the treatment would be taken into account, as well as potential side effects and the severity of their symptoms at that time. The GRADE working group would likely refer to this as a "fully contextualised approach", accounting for all aspects of the specific intervention in order to set thresholds for benefit (Zeng 2021). For this review we adopted a "minimally contextualised approach" and rated imprecision for each outcome according to specific, defined thresholds (as described in Methods). However, if the thresholds used are inappropriate then this may affect the certainty of the evidence (by a maximum of one level). 

#### Other considerations

We did not rate down the certainty of the evidence for other reasons. Publication bias is usually assessed as part of this domain. Although we are aware that this is an issue with many systematic reviews, we did not find strong indications of publication bias with this review. We only identified two ongoing trials, both of which considered antivirals. We are uncertain whether these trials were conducted and remain unpublished, or whether the trials were never completed. However, the evidence for antivirals is already of very low certainty, so the inclusion of these studies as a potential risk of publication bias would not affect the conclusions of this review. 

### Potential biases in the review process

We made some small changes to the review process following the publication of our protocol (Webster 2021b). 

Firstly, we planned to use the Cochrane Pregnancy and Childbirth Trustworthiness Tool to assess the included studies. We had planned to exclude any study where there were concerns (as identified with this tool) from the main analyses. However, as described above, we were unable to determine whether most of the included studies would pass the screening tool, either due to a lack of reporting in the original articles, or because we were unable to contact the authors to resolve any issues. If these studies were subsequently found to have genuine concerns over research integrity then this would further undermine our confidence in the findings of the review. However, as the evidence for these interventions is almost all very low‐certainty, we considered that this would not greatly impact the findings of the review. 

We also identified that our outcome "improvement in vertigo" may not capture an important change in vertigo. Therefore, we added a sensitivity analysis for this outcome. For our main analysis we considered any improvement in vertigo, as pre‐planned. However, we also looked at whether considering "complete resolution of vertigo, or a substantial improvement in vertigo", would impact on the effect estimates. We did note that the point estimate and confidence intervals were typically shifted when using this analysis (in favour of betahistine), but the evidence remained very low‐certainty, therefore we cannot draw any firm conclusions from this exploratory approach. 

### Agreements and disagreements with other studies or reviews

A number of other reviews have considered systemic pharmacological interventions for Ménière's disease. The majority of these have considered either betahistine or diuretics. We did not identify any reviews that looked specifically at antivirals or corticosteroids. 

#### Betahistine

Many of the existing reviews that consider betahistine used slightly different inclusion criteria from those applied in this review, therefore the selection of included studies differs slightly. In particular, a number of reviews included data from cross‐over trials, and many included studies with less than three months of follow‐up. We considered that data from cross‐over trials may be unreliable, due to the fluctuation in symptoms over time. We also considered that follow‐up times of less than three months were likely to be insufficient to assess the efficacy of any treatment, as participants may not experience many vertigo attacks in such a short period of time. Despite this, many of the reviews we identified also conclude that the evidence base for the use of betahistine is lacking, and that there is uncertainty over the efficacy of treatment (Ahmadzai 2020; Devantier 2020; James 2001; James 2007; Rosenbaum 2017; van Esch 2021). One review only considered studies where combinations of drug treatment had been administered, therefore included different studies to this review (Wright 2015). 

We did identify four reviews that concluded that betahistine was efficacious. Two of these reviews were conducted by the same authors and are now over 20 years old (Claes 1997; Claes 2000), therefore do not include the newer studies identified in this review (Adrion 2016; Albu 2016; Khan 2011; Mira 2003). One review was written by an employee of the pharmaceutical company Abbott, and includes unpublished data on the efficacy of betahistine from trials conducted by a pharmaceutical company (Nauta 2014). Only one of these trials met the inclusion criteria for this review (Duphar B.V. 77.054/M) ‐ most of the studies included a mixed population of participants with vertigo, many of whom did not have a diagnosis of Ménière's disease. The last of these four reviews was conducted in 2015, and is a narrative review (Ramos Alocer 2015). The authors state that the "efficacy and safety of betahistine has been demonstrated in numerous clinical trials", although they appear to have simply reported the summary findings of the original study authors, and included cross‐over trials, as well as trials comparing betahistine to other active treatments. 

#### Diuretics

There were also differences in the types of studies included for some existing reviews on diuretics. Three reviews included cross‐over trials (Claes 1997; Claes 2000; Rosenbaum 2018); another included observational studies, as well as RCTs that compared diuretics to other active treatments, cross‐over trials and those with a short duration of follow‐up (Crowson 2016). As with this review, Rosenbaum 2018 concludes that the evidence for the efficacy of diuretics is very low‐certainty. Crowson 2016, however, suggests that the evidence supports the use of diuretics, but acknowledges that this is "low‐level evidence" (predominantly from observational studies). The two reviews by Claes and Van de Heyning conclude that diuretics have proven efficacy in the long‐term control of vertigo, although this conclusion is only based on the inclusion of two cross‐over trials of diuretics (Claes 1997; Claes 2000). 

Two previous reviews did not identify any studies for inclusion, as they were published before the two included studies in this review (Burgess 2006; James 2007). 

## Authors' conclusions

**Implications for practice**

At present, there is scarce information on the efficacy (and harms) of systemic pharmacological interventions for Ménière's disease. Few randomised controlled trials (RCTs) have been conducted in this area, and those that have typically had methodological issues that lead to the potential for bias in the results. Although some of these interventions are widely used across the world for Ménière's disease, high‐certainty evidence to underpin their use is lacking. 

**Implications for research**

Clearly the lack of high‐certainty RCT evidence for these interventions suggests that well‐conducted studies with larger numbers of participants are required to appropriately assess the efficacy (and potential harms) of these interventions. However, there also needs to be more clarity on which outcomes studies should assess, and when and how to assess them. Vertigo is a notoriously difficult symptom to assess, and there is great variety in the methods used to record and report this symptom in the studies we have identified. 

There is a clear need for consensus on which outcomes are important to people with Ménière’s disease, so that future studies can be designed with this in mind. Development of a core outcome set would be preferable as a guide for future trials. We understand that development of a core outcome set for Ménière's disease was underway, with a project registered on the COMET website (https://www.comet-initiative.org/Studies/Details/818), but we have been unable to identify any results of this project, or ascertain whether it is ongoing. If a core outcome set is developed, this should include details on the recommended methods used to measure outcomes, ensuring that these are validated, reliable tools. Monitoring and reporting of adverse effects should be considered a routine part of any study, and should always occur ‐ this is inconsistent at present. Agreement is also needed on the appropriate times at which outcomes should be measured to adequately assess the different interventions.

Any decisions about which outcomes to measure, how to measure them and when to measure them must be made with input from people with Ménière’s disease, to ensure that the outcomes reported by trialists (and future systematic reviews) are relevant to those with the disease. 

Finally, trialists should be clear about the treatments that participants received before entry to the trial, throughout the trial, and the need for additional treatment during the course of the trial. People with Ménière's disease need to be able to understand whether interventions work in all people with the disease, or whether they might work best during certain phases of the disease ‐ perhaps as a first‐line therapy, or for people in whom other treatments have failed. 

## History

Protocol first published: Issue 12, 2021

## Appendices

### Appendix 1. AAO‐HNS definition of Ménière's disease

Definite Ménière's disease:

- Two or more spontaneous episodes of vertigo, each lasting 20 minutes to 12 hours.
- Audiometrically documented low‐ to medium‐frequency sensorineural hearing loss in one ear, defining the affected ear on at least one occasion before, during or after one of the episodes of vertigo.
- Fluctuating aural symptoms (hearing, tinnitus or fullness) in the affected ear.
- Not better accounted for by another vestibular diagnosis.

Probable Ménière's disease: 

- Two or more spontaneous episodes of vertigo, each lasting 20 minutes to 24 hours.
- Fluctuating aural symptoms (hearing, tinnitus or fullness) in the affected ear.
- Not better accounted for by another vestibular diagnosis.

Taken from Lopez‐Escamez 2015.

### Appendix 2. Search strategy

 This search strategy has been designed to identify all relevant studies for a suite of reviews on various interventions for Ménière's disease. 

**Table:**

**CENTRAL (CRS) **	**Cochrane ENT Register (CRS) **	**MEDLINE (Ovid) **
1 MESH DESCRIPTOR Endolymphatic Hydrops EXPLODE ALL AND CENTRAL:TARGET 2 meniere*):AB,EH,KW,KY,MC,MH,TI,TO AND CENTRAL:TARGET 3 (endolymphatic near hydrops):AB,EH,KW,KY,MC,MH,TI,TO AND CENTRAL:TARGET 4 (labyrinth* near hydrops):AB,EH,KW,KY,MC,MH,TI,TO AND CENTRAL:TARGET 5 (labyrinth* near syndrome):AB,EH,KW,KY,MC,MH,TI,TO AND CENTRAL:TARGET 6 (aural near vertigo):AB,EH,KW,KY,MC,MH,TI,TO AND CENTRAL:TARGET 7 (labyrinth* near vertigo):AB,EH,KW,KY,MC,MH,TI,TO AND CENTRAL:TARGET 8 (cochlea near hydrops):AB,EH,KW,KY,MC,MH,TI,TO AND CENTRAL:TARGET 9 (vestibular near hydrops):AB,EH,KW,KY,MC,MH,TI,TO AND CENTRAL:TARGET 10 #1 OR #2 OR #3 OR #4 OR #5 OR #6 OR #7 OR #8 OR #9 AND CENTRAL:TARGET	1 MESH DESCRIPTOR Endolymphatic Hydrops EXPLODE ALL AND INREGISTER 2 (meniere*):AB,EH,KW,KY,MC,MH,TI,TO AND INREGISTER 3 (endolymphatic near hydrops):AB,EH,KW,KY,MC,MH,TI,TO AND INREGISTER 4 (labyrinth* near hydrops):AB,EH,KW,KY,MC,MH,TI,TO AND INREGISTER 5 (labyrinth* near syndrome):AB,EH,KW,KY,MC,MH,TI,TO AND INREGISTER 6 (aural near vertigo):AB,EH,KW,KY,MC,MH,TI,TO AND INREGISTER 7 (labyrinth* near vertigo):AB,EH,KW,KY,MC,MH,TI,TO AND INREGISTER 8 (cochlea near hydrops):AB,EH,KW,KY,MC,MH,TI,TO AND INREGISTER 9 (vestibular near hydrops):AB,EH,KW,KY,MC,MH,TI,TO AND INREGISTER 10 #1 OR #2 OR #3 OR #4 OR #5 OR #6 OR #7 OR #8 OR #9 AND INREGISTER 11 INREGISTER 12 * AND CENTRAL:TARGET 13 #11 NOT #12 14 #10 AND #13	1 exp Endolymphatic Hydrops/ 2 meniere*.ab,ti. 3 (endolymphatic adj3 hydrops).ab,ti. 4 (labyrinth* adj3 hydrops).ab,ti. 5 (labyrinth* adj3 syndrome).ab,ti. 6 (aural adj3 vertigo).ab,ti. 7 (labyrinth* adj3 vertigo).ab,ti. 8 (cochlea adj3 hydrops).ab,ti. 9 (vestibular adj3 hydrops).ab,ti. 10 1 or 2 or 3 or 4 or 5 or 6 or 7 or 8 or 9 11 randomized controlled trial.pt. 12 controlled clinical trial.pt. 13 randomized.ab. 14 placebo.ab. 15 drug therapy.fs. 16 randomly.ab. 17 trial.ab. 18 groups.ab. 19 11 or 12 or 13 or 14 or 15 or 16 or 17 or 18 20 exp animals/ not humans.sh. 21 19 not 20 22 10 and 21
**Embase (Ovid) **	**Web of Science Core Collection (Web of Knowledge) **	**Trial Registries **
1 exp Meniere disease/ 2 meniere*.ab,ti. 3 (endolymphatic adj3 hydrops).ab,ti. 4 (labyrinth* adj3 hydrops).ab,ti. 5 (labyrinth* adj3 syndrome).ab,ti. 6 (aural adj3 vertigo).ab,ti. 7 (labyrinth* adj3 vertigo).ab,ti. 8 (cochlea adj3 hydrops).ab,ti. 9 (vestibular adj3 hydrops).ab,ti. 10 1 or 2 or 3 or 4 or 5 or 6 or 7 or 8 or 9 11 Randomized controlled trial/ 12 Controlled clinical study/ 13 Random$.ti,ab. 14 randomization/ 15 intermethod comparison/ 16 placebo.ti,ab. 17 (compare or compared or comparison).ti. 18 ((evaluated or evaluate or evaluating or assessed or assess) and (compare or compared or comparing or comparison)).ab. 19 (open adj label).ti,ab. 20 ((double or single or doubly or singly) adj (blind or blinded or blindly)).ti,ab. 21 double blind procedure/ 22 parallel group$1.ti,ab. 23 (crossover or cross over).ti,ab. 24 ((assign$ or match or matched or allocation) adj5 (alternate or group$1 or intervention$1 or patient$1 or subject$1 or participant$1)).ti,ab. 25 (assigned or allocated).ti,ab. 26 (controlled adj7 (study or design or trial)).ti,ab. 27 (volunteer or volunteers).ti,ab. 28 human experiment/ 29 trial.ti. 30 11 or 12 or 13 or 14 or 15 or 16 or 17 or 18 or 19 or 20 or 21 or 22 or 23 or 24 or 25 or 26 or 27 or 28 or 29 31 (random$ adj sampl$ adj7 ("cross section$" or questionnaire$1 or survey$ or database$1)).ti,ab. 32 comparative study/ or controlled study/ 33 randomi?ed controlled.ti,ab. 34 randomly assigned.ti,ab. 35 32 or 33 or 34 36 31 not 35 37 Cross‐sectional study/ 38 randomized controlled trial/ or controlled clinical study/ or controlled study/ 39 (randomi?ed controlled or control group$1).ti,ab. 40 38 or 39 41 37 not 40 42 (((case adj control$) and random$) not randomi?ed controlled).ti,ab. 43 (Systematic review not (trial or study)).ti. 44 (nonrandom$ not random$).ti,ab. 45 Random field$.ti,ab. 46 (random cluster adj3 sampl$).ti,ab. 47 (review.ab. and review.pt.) not trial.ti. 48 we searched.ab. 49 review.ti. or review.pt. 50 48 and 49 51 update review.ab. 52 (databases adj4 searched).ab. 53 (rat or rats or mouse or mice or swine or porcine or murine or sheep or lambs or pigs or piglets or rabbit or rabbits or cat or cats or dog or dogs or cattle or bovine or monkey or monkeys or trout or marmoset$1).ti. and animal experiment/ 54 36 or 41 or 42 or 43 or 44 or 45 or 46 or 47 or 50 or 51 or 52 55 30 not 54 56 10 and 55	# 12 #11 AND #10 Indexes=SCI‐EXPANDED, SSCI, A&HCI, CPCI‐S, CPCI‐SSH, BKCI‐S, BKCI‐SSH, ESCI, CCR‐EXPANDED, IC Timespan=All years # 11 TOPIC: ((randomised OR randomized OR randomisation OR randomisation OR placebo* OR (random* AND (allocat* OR assign*) ) OR (blind* AND (single OR double OR treble OR triple) ))) Indexes=SCI‐EXPANDED, SSCI, A&HCI, CPCI‐S, CPCI‐SSH, BKCI‐S, BKCI‐SSH, ESCI, CCR‐EXPANDED, IC Timespan=All years # 10 #9 OR #8 OR #7 OR #6 OR #5 OR #4 OR #3 OR #2 OR #1 Indexes=SCI‐EXPANDED, SSCI, A&HCI, CPCI‐S, CPCI‐SSH, BKCI‐S, BKCI‐SSH, ESCI, CCR‐EXPANDED, IC Timespan=All years # 9 TOPIC: (vestibular NEAR/3 hydrops) Indexes=SCI‐EXPANDED, SSCI, A&HCI, CPCI‐S, CPCI‐SSH, BKCI‐S, BKCI‐SSH, ESCI, CCR‐EXPANDED, IC Timespan=All years # 8 TOPIC: (cochlea NEAR/3 hydrops) Indexes=SCI‐EXPANDED, SSCI, A&HCI, CPCI‐S, CPCI‐SSH, BKCI‐S, BKCI‐SSH, ESCI, CCR‐EXPANDED, IC Timespan=All years # 7 TOPIC: (labyrinth* NEAR/3 vertigo) Indexes=SCI‐EXPANDED, SSCI, A&HCI, CPCI‐S, CPCI‐SSH, BKCI‐S, BKCI‐SSH, ESCI, CCR‐EXPANDED, IC Timespan=All years # 6 TOPIC: (labyrinth* adj3 vertigo) Indexes=SCI‐EXPANDED, SSCI, A&HCI, CPCI‐S, CPCI‐SSH, BKCI‐S, BKCI‐SSH, ESCI, CCR‐EXPANDED, IC Timespan=All years # 5 TOPIC: (aural NEAR/3 vertigo) Indexes=SCI‐EXPANDED, SSCI, A&HCI, CPCI‐S, CPCI‐SSH, BKCI‐S, BKCI‐SSH, ESCI, CCR‐EXPANDED, IC Timespan=All years # 4 TOPIC: (labyrinth* NEAR/3 syndrome) Indexes=SCI‐EXPANDED, SSCI, A&HCI, CPCI‐S, CPCI‐SSH, BKCI‐S, BKCI‐SSH, ESCI, CCR‐EXPANDED, IC Timespan=All years # 3 TOPIC: (labyrinth* NEAR/3 hydrops) Indexes=SCI‐EXPANDED, SSCI, A&HCI, CPCI‐S, CPCI‐SSH, BKCI‐S, BKCI‐SSH, ESCI, CCR‐EXPANDED, IC Timespan=All years # 2 TOPIC: (endolymphatic NEAR/3 hydrops) Indexes=SCI‐EXPANDED, SSCI, A&HCI, CPCI‐S, CPCI‐SSH, BKCI‐S, BKCI‐SSH, ESCI, CCR‐EXPANDED, IC Timespan=All years # 1 TOPIC: (meniere*) Indexes=SCI‐EXPANDED, SSCI, A&HCI, CPCI‐S, CPCI‐SSH, BKCI‐S, BKCI‐SSH, ESCI, CCR‐EXPANDED, IC Timespan=All years	Clinicaltrials.gov menieres or meniere or meniere's | Interventional Studies ICTRP Meniere*

The date restrictions applied to the September 2022 update searches were as follows:

**Table:**

** **	**September 2022 update**
**CENTRAL**	15/09/2021_TO_14/09/2022:CRSINCENTRAL AND CENTRAL:TARGET
**ENT register**	No new records added to register since search was run
**MEDLINE**	23 limit 22 to ed=20210915‐20220914 24 limit 22 to dt=20210915‐20220914 25 23 or 24
**Embase**	57 limit 56 to dd=20210915‐20220914
**Web of Science**	Timespan: 2021‐09‐15 to 2022‐09‐14 (Index Date)
**ClinicalTrials.gov**	First posted from 09/15/2021 to 09/14/2022
**ICTRP**	Date of registration after 15/09/2021
**Google Scholar**	Year: 2021 or 2022

### Appendix 3. Trustworthiness Screening Tool

This screening tool has been developed by Cochrane Pregnancy and Childbirth. It includes a set of predefined criteria to select studies that, based on available information, are deemed to be sufficiently trustworthy to be included in the analysis. These criteria are:

**Research governance**

- Are there any retraction notices or expressions of concern listed on the Retraction Watch Database relating to this study?
- Was the study prospectively registered (for those studies published after 2010)? If not, was there a plausible reason?
- When requested, did the trial authors provide/share the protocol and/or ethics approval letter?
- Did the trial authors engage in communication with the Cochrane Review authors within the agreed timelines?
- Did the trial authors provide IPD data upon request? If not, was there a plausible reason?

**Baseline characteristics**

- Is the study free from characteristics of the study participants that appear too similar (e.g. distribution of the mean (SD) excessively narrow or excessively wide, as noted by Carlisle 2017)?

**Feasibility**

- Is the study free from characteristics that could be implausible? (e.g. large numbers of women with a rare condition (such as severe cholestasis in pregnancy) recruited within 12 months);
- In cases with (close to) zero losses to follow‐up, is there a plausible explanation?

**Results**

- Is the study free from results that could be implausible? (e.g. massive risk reduction for main outcomes with small sample size)?
- Do the numbers randomised to each group suggest that adequate randomisation methods were used (e.g. is the study free from issues such as unexpectedly even numbers of women ‘randomised’ including a mismatch between the numbers and the methods, if the authors say ‘no blocking was used’ but still end up with equal numbers, or if the authors say they used ‘blocks of 4’ but the final numbers differ by 6)?

Studies assessed as being potentially ‘high risk’ will be not be included in the review. Where a study is classified as ‘high risk’ for one or more of the above criteria we will attempt to contact the study authors to address any possible lack of information/concerns. If adequate information remains unavailable, the study will remain in ‘awaiting classification’ and the reasons and communications with the author (or lack of) described in detail.

The process is described in full in Figure 2. 

## Data and analyses

**Comparison 1:** Betahistine versus placebo/no treatment

Outcome or subgroup title	No. of studies	No. of participants	Statistical method	Effect size
1.1 Improvement in vertigo frequency	2		Risk Ratio (M‐H, Random, 95% CI)	Subtotals only
1.1.1 6 to ≤ 12 months	1	70	Risk Ratio (M‐H, Random, 95% CI)	1.50 [0.98, 2.29]
1.1.2 ≥ 12 months	1	62	Risk Ratio (M‐H, Random, 95% CI)	1.11 [0.93, 1.32]
1.2 Improvement in vertigo frequency: sensitivity analysis for complete/substantial improvement	1		Peto Odds Ratio (Peto, Fixed, 95% CI)	Subtotals only
1.2.1 6 to ≤ 12 months	1	10	Peto Odds Ratio (Peto, Fixed, 95% CI)	13.08 [1.01, 170.31]
1.3 Improvement in vertigo frequency: sensitivity analysis for complete/substantial improvement	1		Risk Ratio (M‐H, Random, 95% CI)	Subtotals only
1.3.1 ≥ 12 months	1	62	Risk Ratio (M‐H, Random, 95% CI)	1.37 [1.04, 1.81]
1.4 Change in vertigo global score at 3 to 6 months	1	34	Mean Difference (IV, Random, 95% CI)	0.70 [‐6.67, 8.07]
1.5 Change in vertigo frequency	3		Mean Difference (IV, Random, 95% CI)	Subtotals only
1.5.1 3 to 6 months	2	117	Mean Difference (IV, Random, 95% CI)	‐1.90 [‐3.05, ‐0.74]
1.5.2 6 to ≤ 12 months	1	214	Mean Difference (IV, Random, 95% CI)	0.63 [‐4.07, 5.33]
1.6 Serious adverse events	1	220	Risk Ratio (M‐H, Random, 95% CI)	1.20 [0.63, 2.29]
1.7 Disease‐specific health‐related quality of life at 6 to ≤ 12 months	1	170	Mean Difference (IV, Random, 95% CI)	0.06 [‐0.17, 0.29]
1.8 Disease‐specific health‐related quality of life at > 12 months	1	62	Risk Ratio (M‐H, Random, 95% CI)	1.34 [1.07, 1.69]
1.9 Change in hearing: continuous data only	3		Mean Difference (IV, Random, 95% CI)	Subtotals only
1.9.1 3 to < 6 months	1	35	Mean Difference (IV, Random, 95% CI)	10.10 [‐1.13, 21.33]
1.9.2 6 to ≤ 12 months	1	113	Mean Difference (IV, Random, 95% CI)	2.64 [‐1.66, 6.94]
1.9.3 > 12 months	1	62	Mean Difference (IV, Random, 95% CI)	1.40 [‐7.10, 9.90]
1.10 Change in hearing: dichotomous data only	2		Peto Odds Ratio (Peto, Fixed, 95% CI)	Subtotals only
1.10.1 6 to ≤ 12 months	2	82	Peto Odds Ratio (Peto, Fixed, 95% CI)	3.14 [1.28, 7.66]
1.11 Change in tinnitus	2		Mean Difference (IV, Random, 95% CI)	Subtotals only
1.11.1 6 to ≤ 12 months	1	168	Mean Difference (IV, Random, 95% CI)	‐0.06 [‐1.52, 1.39]
1.11.2 ≥ 12 months	1	62	Mean Difference (IV, Random, 95% CI)	0.90 [‐5.55, 7.35]
1.12 Other adverse effects	4		Peto Odds Ratio (Peto, Fixed, 95% CI)	Subtotals only
1.12.1 Headache	4	374	Peto Odds Ratio (Peto, Fixed, 95% CI)	1.16 [0.69, 1.95]
1.12.2 Gastrointestinal disturbance	4	372	Peto Odds Ratio (Peto, Fixed, 95% CI)	1.08 [0.65, 1.78]
1.12.3 Dry mouth	2	301	Peto Odds Ratio (Peto, Fixed, 95% CI)	0.30 [0.05, 1.95]
1.13 Other adverse effects	2		Risk Ratio (M‐H, Random, 95% CI)	Subtotals only
1.13.1 Sleep disturbance	2	255	Risk Ratio (M‐H, Random, 95% CI)	1.43 [0.47, 4.38]

**Comparison 2:** Diuretic versus placebo/no treatment

Outcome or subgroup title	No. of studies	No. of participants	Statistical method	Effect size
2.1 Improvement in vertigo frequency	1		Risk Ratio (M‐H, Random, 95% CI)	Subtotals only
2.1.1 6 to ≤ 12 months	1	70	Risk Ratio (M‐H, Random, 95% CI)	1.69 [1.13, 2.53]
2.2 Change in vertigo frequency	1		Mean Difference (IV, Random, 95% CI)	Subtotals only
2.2.1 3 to < 6 months	1	220	Mean Difference (IV, Random, 95% CI)	‐2.44 [‐4.98, 0.10]
2.3 Change in disease‐specific health‐related quality of life	1		Mean Difference (IV, Random, 95% CI)	Subtotals only
2.3.1 3 to < 6 months	1	220	Mean Difference (IV, Random, 95% CI)	2.94 [‐3.86, 9.74]
2.4 Change in hearing: continuous data only	1		Mean Difference (IV, Random, 95% CI)	Subtotals only
2.4.1 3 to < 6 months	1	220	Mean Difference (IV, Random, 95% CI)	‐1.43 [‐3.88, 1.02]
2.5 Change in hearing: dichotomous data only	1		Risk Ratio (M‐H, Random, 95% CI)	Subtotals only
2.5.1 6 to ≤ 12 months	1	72	Risk Ratio (M‐H, Random, 95% CI)	1.77 [1.07, 2.91]
2.6 Change in tinnitus	1		Mean Difference (IV, Random, 95% CI)	Subtotals only
2.6.1 3 to < 6 months	1	220	Mean Difference (IV, Random, 95% CI)	1.89 [‐4.96, 8.74]

**Comparison 3:** Antiviral versus placebo/no treatment

Outcome or subgroup title	No. of studies	No. of participants	Statistical method	Effect size
3.1 Improvement in vertigo frequency at 3 to < 6 months	1	23	Risk Ratio (M‐H, Random, 95% CI)	1.38 [0.28, 6.75]
3.2 Change in vertigo frequency at 3 to < 6 months	1		Mean Difference (IV, Random, 95% CI)	Subtotals only
3.3 Disease‐specific health‐related quality of life at 3 to < 6 months	1		Mean Difference (IV, Random, 95% CI)	Subtotals only
3.4 Change in hearing at 3 to < 6 months	1	16	Mean Difference (IV, Random, 95% CI)	4.30 [‐13.94, 22.54]

**Comparison 4:** Corticosteroids versus placebo/no treatment

Outcome or subgroup title	No. of studies	No. of participants	Statistical method	Effect size
4.1 Improvement in vertigo at > 12 months	1		Risk Ratio (M‐H, Random, 95% CI)	Subtotals only
4.2 Improvement in vertigo at > 12 months: sensitivity analysis for complete/substantial improvement	1		Peto Odds Ratio (Peto, Fixed, 95% CI)	Subtotals only
4.3 Change in vertigo frequency at 3 to < 6 months	1		Mean Difference (IV, Random, 95% CI)	Subtotals only
4.4 Disease‐specific health‐related quality of life at > 12 months	1		Peto Odds Ratio (Peto, Fixed, 95% CI)	Subtotals only
4.5 Other adverse effects	1		Peto Odds Ratio (Peto, Fixed, 95% CI)	Subtotals only
4.5.1 Steroid‐related side effects	1	16	Peto Odds Ratio (Peto, Fixed, 95% CI)	7.39 [0.15, 372.38]

## Characteristics of studies

### Characteristics of included studies [ordered by study ID]

**Table:** Adrion 2016

***Study characteristics***		
Methods	Multicentre, randomised, double‐blind, placebo‐controlled, 3‐arm, parallel‐group phase III trial. Duration of treatment was 9 months, with further follow‐up for an additional 3 months. For the purposes of this review we have pooled data from the low‐dose and high‐dose betahistine groups.	
Participants	**Setting: ** Recruited from outpatient ENT or neurology departments of 14 German university hospitals. Treated as outpatients. Participants were enrolled from March 2008 until November 2013. **Sample size:** **Number randomised: **221 participants **Number completed: **181 participants **Participant baseline characteristics** **Age: ** Low‐dose betahistine group: mean 56.1 years (SD 11.1) High‐dose betahistine group: mean 56.1 years (SD 12.6) Placebo group: mean 54.5 years (SD 12.8) **Gender: ** Low‐dose betahistine group: 39 males (53%); 34 females (47%) High‐dose betahistine group: 35 males (47%); 39 females (53%) Placebo group: 35 males (47%); 39 females (53%) **Probable/definite Ménière's disease: ** All participants had definite disease **Duration of disease: ** Not reported **Attack frequency at baseline:** Not reported. Inclusion criterion was at least 2 attacks per month for 3 consecutive months preceding enrolment **Hearing loss at baseline:** Low‐dose betahistine group: At 250 Hz: 32.8 dB hearing threshold (SD 16.0) At 500 Hz: 36.5 dB hearing threshold (SD 19.2) At 1000 Hz: 37.6 dB hearing threshold (SD 19.7) At 2000 Hz: 38.7 dB hearing threshold (SD 19.3) High‐dose betahistine group: At 250 Hz: 29.6 dB hearing threshold (SD 16.0) At 500 Hz: 35.4 dB hearing threshold (SD 19.9) At 1000 Hz: 34.4 dB hearing threshold (SD 21.3) At 2000 Hz: 37.9 dB hearing threshold (SD 18.5) Placebo group: At 250 Hz: 29.4 dB hearing threshold (SD 18.2) At 500 Hz: 33.6 dB hearing threshold (SD 20.0) At 1000 Hz: 35.3 dB hearing threshold (SD 20.7) At 2000 Hz: 35.8 dB hearing threshold (SD 19.9) **Measure of tinnitus at baseline: ** Reported using the mean score for each item on the MiniTF; range 0 to 2, higher scores represent worse quality of life Low‐dose betahistine group: mean score on MiniTF 0.807 (SD 0.531) High‐dose betahistine group: mean score on MiniTF 0.733 (SD 0.482) Placebo group: mean score on MiniTF 0.765 (SD 0.564) **Number of participants with bilateral disease:** Not reported **Inclusion criteria:** Aged 18 to 80 years. Fulfilling AAO‐HNS 1995 criteria for definite unilateral or bilateral Ménière's disease. In an active phase of the disease with at least 2 episodes of vertigo attacks per month in at least 3 consecutive months before enrolment. **Exclusion criteria:** Other central or peripheral vestibular disorders (e.g. vestibular migraine, BPPV, paroxysmal brainstem attacks, phobic postural vertigo). Known contraindications or sensitivity to betahistine, e.g. bronchial asthma, phaeochromocytoma, treatment with other antihistamines, gastric/duodenal ulcers or severe liver/kidney dysfunction. Severe coronary heart disease/heart failure. Persistent uncontrolled hypertension (systolic BP > 180 mmHg or diastolic > 100 mmHg). Life expectancy less than 12 months, other serious illness or complex diseases that might confound treatment assessment. Participation in another trial with an investigational product/device within the preceding 30 days. Previous participation in the study. Planned participation in another trial. Pregnant/breastfeeding women or women contemplating pregnancy. **Diagnosis of M**é**ni**è**re's disease:** AAO‐HNS 1995 criteria for definite disease	
Interventions	**Intervention A (n = 73 randomised, n = 63 completed)** Low‐dose betahistine; 48 mg was administered per day (2 x 24 mg tablets plus one placebo tablet) **Intervention B (n = 74 randomised, n = 59 completed)** High‐dose betahistine; 72 mg was administered per day (3 x 24 mg tablets) **Comparator (n = 74 randomised, n = 59 completed)** Placebo; 3 placebo tablets were administered per day Note: the number of participants who completed full follow‐up (to 9 months) may be lower. The number of 'completers' stated above includes those who contributed some data at the 7‐ to 9‐month time point, but there may have been further dropout before the full 9 months follow‐up. **Background interventions administered to all participants** None reported. Concomitant medication was permitted, except antihistamines.	
Outcomes	**Primary outcomes relevant to this review:** **Improvement in vertigo** Not reported **Change in vertigo** Reported as marginal mean attack rates per 30 days at 9 months, from a negative binomial mixed‐effects model (accounting for dropouts during the trial) **Serious adverse events** Assessed and reported (serious adverse events and treatment‐emergent serious adverse events) **Secondary outcomes relevant to this review:** **Disease‐specific health‐related quality of life** Assessed with the DHI. Results are reported as the mean score for each item (rather than a total score) to account for missing data. Range is therefore 0 to 4 points in original publication (rather than full score which has a range of 0 to 100). **Hearing** Assessed as change from baseline in pure tone audiometry at 4 different frequencies **Tinnitus ** Assessed with the MiniTF. Results are reported as the mean score for each item (rather than a total score) to account for missing data. Range is therefore 0 to 2 points (rather than full score which has a range of 0 to 24). **Other adverse effects** No numeric data are reported; a narrative summary of adverse events was provided **Other outcomes reported in the study:** Attack rate ratio Proportion of participants with longer attack duration Proportion of participants with greater attack severity Vestibular disorders activities of daily living Peak slow phase velocity for cool water irrigation and warm water irrigation Number of participants who prematurely terminated the study because of a treatment‐emergent adverse event Acoustic evoked potential	
Funding sources	Quote: "This study was not industry supported. The study was supported by grants from the German Federal Ministry of Education and Research (Bundesministerium für Bildung und Forschung (BMBF), support code 01KG0708; sponsor’s protocol code no 04T‐617). This work was supported by the German Centre for Vertigo and Balance Disorders (DSGZ), University Hospital Munich, Campus Grosshadern, Munich, Germany. The funder had no role in the design, management, data collection, analyses, or interpretation of the data or in the writing of the manuscript or the decision to submit for publication."	
Declarations of interest	Quote: "All authors [...] declare: support from the German Federal Ministry of Education and Research and the German Centre for Vertigo and Balance Disorders for the submitted work; MS is joint chief editor of the Journal of Neurology, editor‐in‐chief of Frontiers of Neuro‐otology, and section editor of F1000; MS has received speaker honorariums from Abbott, Actelion, UCB SA Belgium, GlaxoSmithKline, TEVA GmbH, Biogen, Pierre Fabre, Eisai GmbH, MSD Sharp & Dohme, and Hennig Pharma; MS has worked as a consultant for Abbott; CSF has received travel grants from Abbott and Pierre Fabre; RG reports grants from the German Federal Ministry of Education and Research and the Volkswagen Foundation, and non‐financial support from Interacoustics, outside the submitted work; the other authors declare no interests. According to a contract approved by the German Federal Ministry of Education and Research (BMBF/DLR), University of Munich, and University Hospital of Munich, Abbott had access to the data after the study and statistical analyses were completed in order to use the data for approval of betahistine for the treatment of Meniere’s disease. Appropriate financial compensation was paid for this service, which was approved by the DLR, University of Munich, and University Hospital of Munich. Abbott did not have any influence on the analyses or interpretation of the data or on the content or form of the manuscript."	
Notes	**Research integrity checklist:** No retractions or expressions of concern The trial was prospectively registered No concerns over baseline characteristics of participants Plausible loss to follow‐up was reported No implausible results are reported Blocked randomisation was used, which accounts for similar numbers allocated to each group	
***Risk of bias***		
**Bias**	**Authors' judgement**	**Support for judgement**
Random sequence generation (selection bias)	Low risk	Quote: "The concealed allocation was performed by an internet based randomisation schedule (https://wwwapp.ibe.med.uni‐muenchen.de/randoulette), stratified by study site. The fixed block size was three (starting with six), which was not disclosed during the trial. The random number list was generated by an investigator with no clinical involvement in the trial." Comment: computer‐generated randomisation list.
Allocation concealment (selection bias)	Low risk	Comment: central allocation by a third party using computerised randomisation software as described above.
Blinding of participants and personnel (performance bias) All outcomes	Low risk	Quote: "Patients, clinicians, core laboratories, and trial staff (data analysts, statisticians) were blind to treatment allocation". "Betahistine dihydrochloride tablets were over‐encapsulated with mannitol and aerosil as filling material. Capsules containing the active ingredient were refilled from original pharmacy packaging into vials under sterile conditions and relabelled by the pharmacy of the university hospital of the University of Heidelberg. In the control group, an identically appearing capsule filled with mannitol and aerosil but not containing any active ingredient was administered as placebo." Comment: placebo and active treatments were identical in appearance. Participants and study personnel are explicitly stated to be blinded.
Blinding of outcome assessment (detection bias) All outcomes	Low risk	Quote: "Patients, clinicians, core laboratories, and trial staff (data analysts, statisticians) were blind to treatment allocation." "all raw patient ratings (this is, the patient’s opinion of the occurrence of vertigo episodes) were evaluated in a blinded manner by trained professionals (CSF; CA) at the site of the principal investigator." Comment: participants self‐rated their vertigo symptoms. Data were also analysed by blinded trial personnel.
Incomplete outcome data (attrition bias) All outcomes	High risk	Comment: unavailability of vertigo diary information was 15/74 (20%) for placebo, 10/73 (14%) for low‐dose betahistine and 15/74 (20%) for high‐dose betahistine. This represents a substantial loss of data. Given that the difference in effect size for the primary outcome was small ("compared with placebo, attack rate ratios were 1.036 (95% confidence interval 0.942 to 1.140) and 1.012 (0.919 to 1.114) for low dose and high dose betahistine, respectively") this loss of data introduces a high risk of bias. Analyses are presented in the article with imputation and adjustment for missing data, but there is still the potential for bias in the estimates due to the sizeable dropout.
Selective reporting (reporting bias)	High risk	Comment: the analysis plan was modified from the protocol (although this was likely to be appropriate) to account for missing data. Some outcomes are not fully reported in the article, nor in the supplementary material, for example adverse effects for individual symptoms, which are specifically listed in the protocol: flush[ing] novel/severe vertigo or dizziness tachycardia severe persisting headache hypotonia (systolic blood pressure < 100 mmHg) increase of alanine aminotransferase level > 2 times the upper limit of the normal range or higher bronchospasm Quincke’s oedema (oedema of the upper respiratory tract or the mucosa) No data are reported at the 12‐month follow‐up, although this was specified in the protocol. However, the authors have supplied us with some data for vertigo outcomes at the 10‐ to 12‐month time point, which indicates no difference in the attack rate between groups at this point (C. Adrion, personal communication).
Other bias	Low risk	Comment: no other concerns were noted.

**Table:** Albu 2016

***Study characteristics***		
Methods	Parallel‐group, triple‐blinded RCT with 24 months duration of treatment and follow‐up	
Participants	**Setting: ** Multicentre trial conducted at 4 sites in Romania and 1 site in Italy. Participants recruited from ENT or otology clinics and managed as outpatients. Trial conducted from January 2009 until June 2013. **Sample size:** **Number randomised:** 66 participants **Number completed:** 62 participants **Participant baseline characteristics** **Age: ** Not reported **Gender: ** High‐dose betahistine group: 15 males (45.5%); 18 females (54.5%) Control group: 12 males (36.4%); 21 females (63.6%) **Probable/definite Ménière's disease: ** All participants had definite disease **Duration of disease: ** Not reported **Attack frequency at baseline: ** Not reported. All participants were required to have an attack frequency of at least 4 episodes per month during the 3 preceding months. **Hearing loss at baseline:** Betahistine group: mean hearing loss 54.6 dB HL (SD 15.2) Control group: mean hearing loss 51.4 (SD 13.6) **Measure of tinnitus at baseline: ** Betahistine group: mean THI 28.3 (SD 14.8) Control group: mean THI 27.7 (SD 16.7) **Number of participants with bilateral disease:** All participants had unilateral disease **Inclusion criteria:** Adult participants with unilateral, definite Ménière's disease according to the AAO‐HNS 1995 criteria. Episodes of spontaneous vertigo lasting between 20 minutes and 12 hours. Mean of 4 or more vertigo spells per month during the 3 months before trial entry. Failure to control symptoms with a 6‐month trial of low‐salt diet, caffeine and nicotine avoidance. **Exclusion criteria:** Bilateral disease. Other peripheral or central vestibular syndromes. Middle ear pathology. Noise‐induced hearing loss. Previous IT gentamicin or corticosteroid use. Previous ablative ear surgery. Allergy to betahistine. **Diagnosis of M**é**ni**è**re's disease:** AAO‐HNS 1995 criteria for definite Ménière's disease	
Interventions	**Intervention (n = 33 randomised, n = 30 completed)** High‐dose betahistine; participants received 48 mg betahistine 3 times daily (total 144 mg) **Comparator (n = 33 randomised, n = 32 completed)** Placebo; participants received identical appearing placebo pills Duration of treatment was not provided but was assumed to be for the duration of the trial (2 years) **Background interventions administered to all participants** All participants received intratympanic dexamethasone: "Dexamethasone was injected under the microscope [...]: in the supine position, the patient turned the head 45° toward the unaffected ear. Local anesthesia of the tympanic membrane was achieved and dexamethasone (4 mg/mL [*total quantity not stated*]) was injected through a 22‐gauge spinal needle and 1‐mL syringe to fill the middle ear. Patients were instructed to keep the supine position with the treated ear facing upward for 30 min avoiding swallowing or talking. The ITD protocol consisted of three consecutive daily injections."	
Outcomes	**Primary outcomes relevant to this review:** **Improvement in vertigo** Assessed using the AAO‐HNS 1995 class of vertigo **Change in vertigo** Not reported **Serious adverse events** Not apparently systematically assessed or reported. Narrative summary of adverse events only. **Secondary outcomes relevant to this review:** **Disease‐specific health‐related quality of life** Assessed with the AAO‐HNS 1995 FLS **Hearing** Assessed with pure tone audiometry at 24 months **Tinnitus ** Assessed with the THI at 24 months **Other adverse effects** Not apparently systematically assessed or reported. Narrative summary of adverse events only. **Other outcomes reported in the study:** Speech discrimination score Survival curves for attainment of complete and substantial vertigo control Hearing assessed as improvement/unchanged/worsened	
Funding sources	Quote: "No funding was received for this study"	
Declarations of interest	There is no declaration of interest	
Notes	**Research integrity checklist:** No retractions or expressions of concern were identified No prospective trial registration was identified; the authors are unable to supply us with a copy of the original trial protocol Baseline characteristics of the groups are not excessively similar There is little loss to follow‐up, despite the length of the trial (2 years); the authors state that this is because participants were receiving free treatment during the trial The study is free from implausible results Identical numbers of participants were randomised to each group, but block randomisation is not described	
***Risk of bias***		
**Bias**	**Authors' judgement**	**Support for judgement**
Random sequence generation (selection bias)	Low risk	Quote: "For random assignment of patients, a computer‐generated list of random numbers was employed. Randomization was performed by an only investigator (LM) one day before the injection." Comment: computer‐generated randomisation list.
Allocation concealment (selection bias)	Unclear risk	Comment: no methods to conceal allocation were reported.
Blinding of participants and personnel (performance bias) All outcomes	Low risk	Quote: "Group A received a combination of ITD and identical‐appearing placebo pills". "Both the surgeons and the patients were blinded to the treatment". Comment: likely that blinding of participants and personnel was adequate if placebo pills were identical.
Blinding of outcome assessment (detection bias) All outcomes	Unclear risk	Quote: "Audiometric testing and completion of questionnaires were performed by researchers blinded to the surgeons. Throughout the study, all patients kept a diary recording monthly the occurrence of every vertiginous attack" Comment: outcomes were assessed by (blinded) participants and blinded researchers. However, we note that side effects that should have been likely to occur in both groups over the 2‐year follow‐up (nausea, headache, diarrhoea) were only reported for the betahistine group. This may be due to selective reporting, but there is a concern that investigators recorded these symptoms because they were aware of the group allocation. There is no description of how these adverse events were collected and whether this was identical in both groups.
Incomplete outcome data (attrition bias) All outcomes	Low risk	Quote: "Three (sic) patients (1 in Group A and 3 in Group B) were lost to follow‐up." Comment: there was only a moderate difference in the numbers lost to follow‐up across groups (9% in the betahistine group, and 3% in the placebo group). Given the large difference in effect size for the primary outcome (complete vertigo control achieved in 14 patients (44%) from Group A and in 22 patients (73.3%) from Group B, statistically significant (P = 0.01)), it is unlikely that bias would arise from incomplete outcome data.
Selective reporting (reporting bias)	Unclear risk	Comment: no published protocol was identified and the authors are unable to supply a copy of the original trial protocol.
Other bias	High risk	Comment: repeated intratympanic steroid injections were permitted if vertigo was not substantially/completely controlled. Additional treatments were also permitted through the study. It is unclear whether these treatments were offered equally and consistently to the 2 groups of participants. For example, the study states that "If complete or substantial vertigo control was not accomplished, another sequence of [IT dexamethasone] was offered. In patients with persistent vertigo, despite repeated ITD injections, IT gentamicin injection or ablative surgery was offered". 5 participants in the placebo arm received 1 repeat injection, and 6 participants received 2 repeated injections. According to the methods it appears that those who did not achieve complete/substantial control (11 patients, at the end of the study) should have gone on to receive further IT injections, but this does not appear to have been performed. However, this may be due to follow‐up being insufficient.

**Table:** Derebery 2004

***Study characteristics***		
Methods	Parallel‐group, double‐blind randomised controlled trial with 10 days duration of treatment and a total of 6 months of follow‐up	
Participants	**Setting: ** Single‐centre trial, conducted in the USA. Study dates are not reported. **Sample size:** **Number randomised:** 24 participants **Number completed:** 23 participants **Participant baseline characteristics** **Age: ** Not reported separately for the groups; the mean age for all participants was 48.3 years (SD 12.0) **Gender: ** Not reported separately for the groups; 6 participants were male and 17 were female **Probable/definite Ménière's disease: ** All definite disease (inclusion criterion) **Duration of disease: ** The average duration of symptoms prior to study inclusion was 3.4 years **Attack frequency at baseline: ** Famciclovir group: 5.3 attacks per week (SD 11.1) Control group: 5.3 attacks per week (SD 7.6) All participants reported at least 2 disabling vertigo spells per month at enrolment **Hearing loss at baseline:** Reported for those with unilateral disease only Famciclovir group: mean hearing threshold 52.2 dB (SD 20.8) Control group: mean hearing threshold 53.4 dB (SD 7.2) **Measure of tinnitus at baseline: ** Famciclovir group: mean 57.0 (SD 14.1) Control group: mean 68.7 (SD 21.4) **Number of participants with bilateral disease:** Famciclovir group: 3/12 Control group: 2/11 **Inclusion criteria:** A diagnosis of definite Ménière's disease according to the AAO‐HNS 1995 criteria. Unilateral or bilateral disease. Active disease, experiencing disabling vertigo spells at least 2 times per month (the need to cease an activity and lie down until the dizzy spell is over, lasting at least 20 minutes). "If the initial diagnosis of Ménière’s disease had been made in the past 3 months, the subject had to wait 3 more months to begin the trial." **Exclusion criteria:** Renal disease. Immunocompromise. Hypersensitivity to famciclovir or penciclovir cream. MR evidence of vestibular schwannoma or other retrocochlear disease. Positive fluorescent treponemal antibody absorption test. Concurrent use of probenecid. **Diagnosis of M**é**ni**è**re's disease:** Definite Ménière's disease according to the AAO‐HNS 1995 criteria	
Interventions	**Intervention (n = 13 randomised, n = 12 completed)** Famciclovir 250 mg 3 times per day for 10 days, followed by 250 mg twice daily for 80 days (total 3‐month course) **Comparator (n = 11 randomised, n = 10 completed)** Placebo; "250mg placebo pills" contents not specified. To be taken 3 times daily for 10 days, then twice daily for 80 days, as above. **Background interventions administered to all participants** None reported	
Outcomes	**Primary outcomes relevant to this review:** **Improvement in vertigo** Assessed as the proportion of participants in each treatment arm who demonstrated a 20% reduction in the number of disabling vertigo episodes at 3 months (as compared with baseline measures) **Change in vertigo** Assessed with the number of dizzy spells **Serious adverse events** Adverse events appear to have been systematically recorded, although a description of serious adverse events is not present **Secondary outcomes relevant to this review:** **Disease‐specific health‐related quality of life** Assessed with the DHI at 3 months **Hearing** Assessed with pure tone audiometry at 0.5 kHz, 1 kHz, 2 kHz and 3 kHz **Tinnitus ** Assessed with the THI. An obvious typographical error in the data means that we are unable to include these results in the analysis. **Other adverse effects** Authors state that "The clinical coordinator also recorded any physiological reactions to the drug, assessing for headaches, gastro‐intestinal problems, fever, increased dizziness, rash, decreased or increased appetite, and cardiovascular changes" **Other outcomes reported in the study:** Global quality of life (SF‐36) AAO‐HNS FLS median score at baseline and 3 months Mean duration of vertiginous episodes Speech discrimination scores	
Funding sources	Quote: "Dr. Derebery received the Percy Award from the American Academy of Otolaryngology–Head and Neck Surgery to support this study. Novartis graciously supplied both active drug and placebo for this trial."	
Declarations of interest	No declarations are made	
Notes	**Research integrity checklist:** No retractions/expressions of concern were identified Trial registration was not applicable (published in 2004) Baseline characteristics of the groups do not appear to be excessively similar Reasons are given for the small loss to follow‐up The study was free from implausible results Similar numbers were allocated to each group, but blocked randomisation was reported	
***Risk of bias***		
**Bias**	**Authors' judgement**	**Support for judgement**
Random sequence generation (selection bias)	Unclear risk	Quote: "Subjects were randomized within blocks of 10 subjects, such that for every 10 subjects, 5 subjects would be randomized to active treatment and 5 subjects would be randomized to placebo". Comment: this is likely to have been achieved with computerised randomisation, but this is not clear from the text.
Allocation concealment (selection bias)	Unclear risk	Comment: no information is reported regarding concealment of allocation to groups.
Blinding of participants and personnel (performance bias) All outcomes	Unclear risk	Comment: described as double‐blind. No further details were given to establish the efficacy of blinding, and study personnel are not specifically stated to be blinded.
Blinding of outcome assessment (detection bias) All outcomes	Low risk	Comment: the majority of outcome data are from participant‐reported outcomes (e.g. frequency of vertigo episodes, according to a diary, DHI, THI etc.). As participants were blinded, this can be considered low risk.
Incomplete outcome data (attrition bias) All outcomes	Low risk	Comment: few dropouts, and reasons balanced across groups ‐ 1 participant in each group required additional treatment. One further dropout was not fully reported (after taking only 1 day of medication) and was excluded from outcome measures. However, inclusion of this participant is unlikely to have impacted the results of this trial.
Selective reporting (reporting bias)	High risk	Comment: no protocol is available to compare. Methods state that follow‐up at 6 months (3 months after discontinuation of treatment) would take place, but no results are reported at this time. This may be because of the failure of efficacy at the primary outcome point.
Other bias	Low risk	Comment: no other concerns identified.

**Table:** Duphar B.V. 77.054/M

***Study characteristics***		
Methods	Single‐centre, randomised, double‐blind, placebo‐controlled, 2‐arm, parallel‐group trial. Duration of treatment and follow‐up was 12 weeks.	
Participants	**Setting:** recruited from the National Ear, Nose and Throat Hospital, based in the UK from 1979 to 1982 **Sample size:** **Number randomised:** 50 participants **Number completed:** 36 participants **Participant baseline characteristics** Reported for those who completed the study **Age: ** Betahistine group: mean 42.9 years (SD not reported) Placebo group: mean 48.8 years (SD not reported) **Gender: ** Betahistine group: 12 males (63%); 7 females (37%) Placebo group: 10 males (59%); 7 females (41%) **Probable/definite Ménière's disease: ** Not reported **Duration of disease: ** Not reported **Attack frequency at baseline:** Betahistine group: mean 5.3 episodes in a 3‐week period Placebo group: mean 3 episodes in a 3‐week period **Hearing loss at baseline:** Not reported **Measure of tinnitus at baseline: ** Not reported **Number of participants with bilateral disease:** Not reported **Inclusion criteria:** Vertigo, likely to be of peripheral origin, either: Ménière’s disease Labyrinthine ischaemia Other forms of peripheral vertigo with unclear pathology Fairly stable symptoms for at least 2 months prior to study entry. Frequency of attacks of at least 1 per month during the 2 months before the study. 33/36 participants were recruited due to a diagnosis of Ménière’s disease. As the majority of participants had Ménière's disease, we included this study in the review. **Exclusion criteria:** Vertigo related to infection of middle ear or sinuses. Vertigo of ocular origin. Spontaneous vertigo with focal neurological symptoms (TIAs). Vertigo of central origin as observed in intracranial tumours, brain stem ischaemia, multiple sclerosis, trauma capitis, epilepsy. Vertigo due to central disorders. Serious cardiac disease, hypertension, orthostatic hypotension, anaemia, diabetes, thyroid disorders, intoxication, syphilis, vertigo of psychological origin, peptic ulcer, phaeochromocytoma or asthma, stroke or MI in the preceding 2 months and pregnant women. **Diagnosis of M**é**ni**è**re's disease:** no diagnostic criteria are stated	
Interventions	**Intervention (number randomised not reported, n = 19 completed):** betahistine 12 mg tablet 3 times daily for 12 weeks (total 36 mg daily) **Comparator (number randomised not reported, n = 17 completed):** placebo; 3 placebo tablets were administered per day for 12 weeks	
Outcomes	**Primary outcomes relevant to this review:** **Improvement in vertigo** Not reported **Change in vertigo** Reported as the number of 'real attacks of vertigo' over a 6‐week period **Serious adverse events** Adverse events appear to have been systematically recorded, although a description of serious adverse events is not present **Secondary outcomes relevant to this review:** **Disease‐specific health‐related quality of life** Not reported **Hearing** Assessed with pure‐tone audiometry, but only reported narratively. No data are presented that are suitable for meta‐analysis. **Tinnitus ** Not reported using a validated instrument **Other adverse effects** A table was presented that included adverse effects experienced by the participants. This included a description of gastrointestinal disturbance and headache. **Other outcomes reported in the study:** Impact of dizziness on quality of life, as assessed by the investigator Investigator's opinion of treatment Vertigo, severity at week 12 Patient/investigator evaluation of treatment Deafness severity rating	
Funding sources	Funded by Duphar Laboratories	
Declarations of interest	No declaration is made	
Notes	**Research integrity checklist:** No retractions/expressions of concern were identified but this study remains unpublished Trial registration was not applicable (conducted in 1983) Baseline characteristics of the groups do not appear to be excessively similar, but there are very limited data to assess Plausible dropout is reported The study was free from implausible results The number randomised to each group is not reported, therefore we are uncertain whether the same number were recruited to each group	
***Risk of bias***		
**Bias**	**Authors' judgement**	**Support for judgement**
Random sequence generation (selection bias)	Unclear risk	Quote: “patients were randomly allocated to treatment with either betahistine or placebo” Comment: no further details are provided regarding the methods used for randomisation.
Allocation concealment (selection bias)	Unclear risk	Comment: no information was provided on any methods used to conceal allocation.
Blinding of participants and personnel (performance bias) All outcomes	Unclear risk	Comment: placebo was used to blind participants. It is unclear whether study personnel were also blinded to group allocations.
Blinding of outcome assessment (detection bias) All outcomes	Low risk	Comment: outcomes are reported by (blinded) participants, therefore we consider these to be at low risk of detection bias.
Incomplete outcome data (attrition bias) All outcomes	High risk	Comment: substantial dropout of 14/50 (28%) participants, and reasons may be related to treatment. Per protocol analysis is suggested to have occurred due to the exclusion of one participant who failed to take their tablets.
Selective reporting (reporting bias)	High risk	Comment: no protocol is available to assess against pre‐specified outcomes. Audiometry was reported in a way that precludes meta‐analysis.
Other bias	High risk	Comment: we had concerns over the measures used to assess vertigo – an unvalidated scoring system was used, and it was unclear how “real attacks of vertigo” were defined.

**Table:** Khan 2011

***Study characteristics***		
Methods	Randomised, placebo‐controlled, parallel‐group trial with 1 year of follow‐up. The duration of treatment was not reported, but could be assumed to be for 1 year.	
Participants	**Setting: ** Conducted in the ENT Department of a military hospital in Lahore, Pakistan. Trial dates from March 2007 until December 2009. **Sample size:** **Number randomised:** 120 participants **Number completed:** 106 participants **Participant baseline characteristics** **Age: ** Diuretic group: mean 49.3 years (SD 6.7) Betahistine group: mean 51.3 years (SD 8.6) Placebo group: mean 50.2 years (SD 5.6) **Gender: ** Diuretic group: 17 males (46%); 20 females (54%) Betahistine group: 14 males (45%); 17 females (55%) Placebo group: 18 males (47%); 20 females (53%) **Probable/definite Ménière's disease: ** Not reported **Duration of disease: ** Not reported **Attack frequency at baseline: ** Not reported **Hearing loss at baseline:** Not reported **Measure of tinnitus at baseline: ** Not reported **Number of participants with bilateral disease:** Not reported **Inclusion criteria:** All new patients from the ENT Department of the Combined Military Hospital, Lahore with clinically confirmed diagnosis of Ménière's disease **Exclusion criteria:** Patients presenting with atypical features. Patients with tinnitus as a major symptom and showing retro‐cochlear deafness on audiometric testing. Patients with acute or chronic middle ear disease presenting with Ménière's like syndrome. Patients allergic to either of the study drugs. Patients with positive VDRL. **Diagnosis of M**é**ni**è**re's disease:** A typical triad of symptoms, i.e. episodic vertigo (at least 2 definitive episodes of vertigo of at least 20 minutes duration), tinnitus and hearing loss (minimum hearing loss of 30 dB in any of 500 Hz, 1000 Hz and 2000 Hz). As the trial was conducted in an ENT hospital department, we assume that diagnosis was made by an ENT specialist.	
Interventions	**Intervention A (n = 40 randomised, n = 37 completed)** Diuretics; amiloride hydrochloride 5 mg, hydrochlorothiazide 50 mg once daily, plus advice for salt restriction (no further information) **Intervention B (n = 40 randomised, n = 31 completed)** Betahistine group; betahistine dihydrochloride 16 mg 3 times daily **Comparator (n = 40 randomised, n = 38 completed)** Multivitamin (placebo) group; multivitamin tablet once daily **Background interventions administered to all participants** None reported	
Outcomes	**Primary outcomes relevant to this review:** **Improvement in vertigo** Assessed using a patient questionnaire which considered the number of attacks and the severity of attacks. Severity was rated as mild (vertigo with no nausea, could perform routine work), moderate (vertigo with nausea, had to suspend work during the attack) and severe (vertigo and nausea and vomiting and had to stay in bed). Improvement was assessed as a decrease in the number or severity of vertigo attacks, or both. It is not clear over what time frame this outcome was assessed, but it is reported at 12 months. **Change in vertigo** Not reported **Serious adverse events** Not reported **Secondary outcomes relevant to this review:** **Disease‐specific health‐related quality of life** Not reported **Hearing** Assessed with subjective feeling of improvement in hearing, which was confirmed with pure tone audiometry (as a change of ≥ 10 dB hearing threshold on the better side). It is unclear why the "better side" was used, unless all participants had bilateral disease. **Tinnitus ** Not assessed with a validated score (rated by participants using a visual analogue scale). Improvement was defined as a change of 2 or more points on this scale. **Other adverse effects** Not reported **Other outcomes reported in the study:** None reported	
Funding sources	Not reported	
Declarations of interest	No declarations are made	
Notes	**Research integrity checklist:** No retractions/expressions of concern were identified No trial registration was identified Baseline characteristics are only reported for age and gender. These variables are not excessively similar across groups, but there is limited information on which to base this judgement. Some loss to follow‐up is reported Very large effect sizes are noted for the active interventions (risk ratios range from 1.5 to 1.8) Equal numbers of participants were allocated to each group without mention of block randomisation	
***Risk of bias***		
**Bias**	**Authors' judgement**	**Support for judgement**
Random sequence generation (selection bias)	Unclear risk	Quote: "The patients were randomly placed in three equal groups" Comment: insufficient information is provided regarding the generation of the randomisation sequence.
Allocation concealment (selection bias)	Unclear risk	Comment: no information is reported regarding concealment of allocation.
Blinding of participants and personnel (performance bias) All outcomes	High risk	Comment: no blinding was reported, and frequency of administration was different for the individual groups (e.g. 3 tablets for betahistine, 1 tablet for diuretics and multivitamin groups), therefore participants may have been aware of their group allocation. Trial personnel administering treatment may not have been blind to treatment allocation.
Blinding of outcome assessment (detection bias) All outcomes	High risk	Comment: no blinding was reported, and frequency of administration was different for the individual groups, therefore participants may have been aware of their group allocation. Outcomes are reported by the participants themselves.
Incomplete outcome data (attrition bias) All outcomes	High risk	Comment: the proportion of missing outcomes compared with observed event risk was enough to induce clinically relevant bias in intervention effect estimate. The reasons for losses to follow‐up were not reported. Discrepancies are present in the number of dropouts through the text and figures.
Selective reporting (reporting bias)	High risk	Comment: no protocol was available. No adverse event assessment was reported, which is unexpected in a trial of medications. Methods state that vertigo frequency and vertigo severity will be assessed separately by participants. However, "improvement" is reported as an improvement in either of these measures. It is unclear why these aspects were assessed separately and then analysed together. It is also unclear how participants would have been judged to have "improved" if there had been improvement in one measure but not the other (e.g. if the frequency had reduced, but the severity increased).
Other bias	High risk	Comment: concerns are present regarding the methods of outcome assessment. Frequency of vertigo was assessed, but the time frame is not reported, and no vertigo diaries were used. PTA was apparently used in the assessment of hearing, but not clear how this was conducted. Unclear why a change in the better side was used as the outcome measure for hearing. Unclear why so many people had an improvement in hearing over the course of the trial, when the disease course was likely to have caused deterioration in hearing.

**Table:** Mira 2003

***Study characteristics***		
Methods	Multicentre, parallel‐group, double‐blind RCT with 3 months total duration of treatment and follow‐up Note: this trial included participants with both BPPV and Ménière's disease. The trial was stratified by condition. Only data that were reported separately for Ménière's disease have been extracted for the review.	
Participants	**Setting: ** Recruited from 11 hospital and university ENT centres across Italy. Both inpatients and outpatients were recruited. Trial conducted from January 1999 to June 2001. **Sample size:** **Number randomised:** 81 participants with Ménière's disease (n = 144 total) **Number completed:** 81 participants Some participants are listed as not attending interim follow‐up, but the authors state that an ITT analysis was conducted, and do not state that any participants were missing from final follow‐up. We therefore assume that complete follow‐up was achieved. **Participant baseline characteristics** Reported for participants with both Ménière's disease and BPPV Number in betahistine group = 75, placebo group = 69 **Age: ** Betahistine group: mean 46.9 years (SD 13.1) Placebo group: mean 48.8 years (SD 14.3) **Gender: ** Betahistine group: 33 males (44%): 42 females (56%) Placebo group: 27 males (39%): 42 females (61%) **Probable/definite Ménière's disease: ** Not reported **Duration of disease:** Betahistine group: mean 31.6 months (SD 55.0)** ** Placebo group: mean 32.5 months (SD 67.3) **Attack frequency at baseline: ** Not reported **Hearing loss at baseline:** Not reported **Measure of tinnitus at baseline: ** Not reported **Number of participants with bilateral disease:** Not reported **Inclusion criteria:** Males and females aged 18 to 65 years old. Signed, informed, written consent. Interfering concomitant therapies withdrawn at least 7 days before the start of the study treatment. Normal laboratory‐documented renal and hepatic function. Adhering to the scheduled procedure. **Exclusion criteria:** Concomitant infectious or cerebrovascular diseases. Diseases that were not compatible with or contraindicated by betahistine. Concomitant therapy with anti‐vertigo drugs. Use of drugs that act on the cerebral circulation, antihistamines, calcium antagonists, anti‐aggregants, thiazide diuretics, corticosteroids and benzodiazepines. Having any major medical or surgical condition or having a terminal disease. **Diagnosis of M**é**ni**è**re's disease:** Stated to be according to the AAO‐HNS 1995 criteria	
Interventions	**Intervention (n = 41 randomised, n = 41 completed)** Betahistine; 16 mg (2 tablets) twice a day, administered at 8 a.m. and 8 p.m. after meals for 3 months **Comparator (n = 40 randomised, n = 40 completed)** Placebo; 2 tablets twice a day, administered at 8 a.m. and 8 p.m. after meals for 3 months. The tablets were indistinguishable by colour, weight and flavour from betahistine tablets, but their composition was not reported. **Background interventions administered to all participants** None reported	
Outcomes	**Primary outcomes relevant to this review:** **Improvement in vertigo** Article reports on the number of people who report an improvement in "intensity score" of vertigo at 1, 2 and 3 months. However, this scale does not apparently assess frequency of vertigo, and clearly does not capture duration of episodes therefore cannot be regarded as a global score. Therefore, no data are available for analysis. **Change in vertigo** The frequency of attacks at 3 months was reported fully for the betahistine group, and partially for the placebo group (with no SD) **Serious adverse events** Adverse events are fully reported, but it is not possible to clearly distinguish which of these were "serious" **Secondary outcomes relevant to this review:** **Disease‐specific health‐related quality of life** The percentage change in DHI scores from baseline was reported, but without any estimate of variance, therefore these data cannot be included in the review **Hearing** Not reported **Tinnitus ** Not reported; tinnitus was only assessed as part of a composite score including aural fullness, nausea and vomiting **Other adverse effects** Assessed and reported **Other outcomes reported in the study:** Number of participants with improvement in associated symptoms Overall quality of life Overall "GISFaV" score ‐ including intensity, duration and associated symptoms of vertigo Duration of attacks Dizziness Assessment Rating Scale Investigator assessment of overall treatment efficacy and acceptance "Doctor's preparedness to treat the patient again with the same treatment" Participant assessment of overall treatment efficacy and acceptance	
Funding sources	Quote: "This study was supported by a grant from Grunenthal‐Formenti, Milan, Italy" (Pharmaceutical company funding)	
Declarations of interest	No declaration is provided	
Notes	**Research integrity checklist:** No retractions or expressions of concern were identified The study was published prior to 2010 and no trial registration was required Baseline characteristics for participants with Ménière's disease cannot be established. However, no concerns according to the full cohort of participants with Ménière's disease and BPPV. Plausible loss to follow‐up during the trial is reported, although all participants appear to have been assessed at the final 3‐month assessment point No implausible results were noted No concerns over randomisation	
***Risk of bias***		
**Bias**	**Authors' judgement**	**Support for judgement**
Random sequence generation (selection bias)	Unclear risk	Quote: "The trial was [...] randomised for a series of four patients; it was balanced among the centres" "[...] 11 hospital and university ENT centres were involved in the study. Each of these was given a series of four patients to recruit. There was a total of at least 140 patients, who were stratified into groups having recurrent vertigo related to Meniere’s disease (MD) and positional paroxysmal vertigo (PPV) of probable vascular origin." Comment: indicative of block randomisation, stratified by condition (MD or PPV). However there is insufficient information about sequence generation.
Allocation concealment (selection bias)	Unclear risk	Quote: "The treatment was assigned to the patient according to the random list of the relevant diagnosis (MD or PPV)." Comment: there was insufficient detail about allocation concealment.
Blinding of participants and personnel (performance bias) All outcomes	Unclear risk	Quote: "The tablets were indistinguishable by colour, weight and flavour. They were supplied in identical packages with a fantasy name to keep the blindness" Comment: the study is described as "double‐blind" suggesting the patients and either the personnel managing the trial, or those administering treatments, or those assessing outcomes were unaware of group assignment. It is clear that patients were blind to whether they received active drug or placebo (as above). No further details were reported regarding study personnel, therefore it is unclear whether blinding was ensured.
Blinding of outcome assessment (detection bias) All outcomes	Low risk	Comment: trial does state that it is "double‐blind" but no further details are reported. However, most outcomes of interest are reported by (blinded) participants, therefore considered low‐risk.
Incomplete outcome data (attrition bias) All outcomes	Unclear risk	Quote: "Fifteen patients (ten in the BE group and five in the PL group) were excluded from the PP analysis because of protocol violations such as interfering concomitant therapies (four in the BE group and three in the PL), not coming to the 2‐month follow‐up visit (three in the BE group and two 2 in the PL) and adverse events (three in the BE group)." "In the intention‐to‐treat analysis, all the randomised patients were considered who had taken at least one dose of the drug being studied, who had carried out at least one complete examination involving all the measurements specified in the protocol and who had not violated the protocol". Comment: the number of patients lost to follow‐up is not clearly reported. No separate details are given for those with Ménière's disease. It is not clear how the ITT analysis and PP analysis differ if participants with protocol violations were excluded from the ITT analysis.
Selective reporting (reporting bias)	High risk	Comment: primary outcome data (number of vertigo attacks per month) is reported incompletely for the placebo arm of this trial. Data are reported as a percentage change from baseline in attack frequency, which seems an unusual choice. No protocol is available to compare pre‐specified outcomes therefore we consider this high‐risk.
Other bias	Low risk	Comment: no other concerns identified.

**Table:** Morales‐Luckie 2005

***Study characteristics***		
Methods	Parallel‐group RCT with 18 weeks duration of treatment and tapering of dose (timing not specified). Further follow‐up for 1 year after discontinuation of steroids.	
Participants	**Setting: ** Single‐centre trial from Mexico. Participants were recruited from a tertiary care hospital in Mexico. Managed as outpatients. Study recruitment dates were not reported. **Sample size:** **Number randomised:** 16 participants **Number completed:** 16 participants **Participant baseline characteristics** **Age: ** Prednisolone group: mean 40.8 years (range 33 to 47 years) Control group: mean 38.8 years (range 32 to 49 years) **Gender:** Not reported ** ** **Probable/definite Ménière's disease: ** Not reported **Duration of disease: ** Prednisolone group: mean 3.6 years Control group: mean 3.3 years **Attack frequency at baseline: ** Prednisolone group: mean 1.27 attacks per day at baseline (95% CI 0.73 to 1.81 attacks) Control group: mean 1.01 attacks per day at baseline (95% CI 0.71 to 1.31 attacks) **Hearing loss at baseline:** Not reported **Measure of tinnitus at baseline: ** Not reported **Number of participants with bilateral disease:** Not reported **Inclusion criteria:** Ménière's disease, apparently according to the AAO‐HNS criteria, although not explicit: "According to Pearson‐Brackmann criteria [Pearson 1985] and the amended 1995 version [AAO‐HNS 1995 criteria], all patients included in this study had severe disability (Scale 3) and limited vertigo control (Class C), and rejected any surgical management." Vertigo under poor control by maintenance treatment (diphenidol 25 mg/day, acetazolamide 250 mg/48 hours orally and a low‐sodium diet (< 1500 mg/day), with advised reductions in consumption of alcohol, caffeine, nicotine and stress). **Exclusion criteria:** Peptic acid disease; diabetes mellitus; hypertension; glaucoma. Other clinical conditions for which glucocorticoid therapy is contraindicated. **Diagnosis of M**é**ni**è**re's disease:** Not stated. Only AAO‐HNS 1995 criteria for grading disability and vertigo control in response to treatment were reported.	
Interventions	**Intervention (n = 8 randomised, n = 8 completed)** Oral prednisolone 0.35 mg/kg/day was administered for 18 weeks, in addition to maintenance treatment. "The [prednisolone] administered was gradually reduced by a 50% dose reduction every 5 days. The drug was definitively withdrawn when a total dose of 2.5 mg was reached." Duration of intervention will therefore vary depending on body weight. **Comparator (n = 8 randomised, n = 8 completed)** No intervention; maintenance treatment only **Background interventions administered to all participants** All patients received maintenance treatment: diphenidol 25 mg/day, acetazolamide 250 mg/48 hours orally and a low‐sodium diet (< 1500 mg/day), with advised reductions in consumption of alcohol, caffeine, nicotine and stress	
Outcomes	**Primary outcomes relevant to this review:** **Improvement in vertigo** Improvement was assessed according to the AAO‐HNS 1995 control of vertigo (frequency) scale as complete, substantial, limited or insignificant improvement or worsening of vertigo **Change in vertigo** Assessed with daily frequency of vertigo episodes **Serious adverse events** There is no report on how adverse events were assessed. Some other (non‐serious) adverse events are specifically reported. **Secondary outcomes relevant to this review:** **Disease‐specific health‐related quality of life** Reported as a change in disability status as assessed with a variant of the AAO‐HNS 1995 Functional Level Scale. Scale used in this article ranges from 0 to 3: 0 = No disability 1 = Mild disability: mild unsteadiness or dizziness that precludes working in a hazardous environment 2 = Moderate disability: moderate unsteadiness or dizziness that results in necessity for a sedentary occupation 3 = Severe disability: symptoms exclude gainful employment **Hearing** Assessed with pure tone audiometry and speech discrimination scores. Not fully reported ‐ no numeric data suitable for meta‐analysis. **Tinnitus ** No data from a validated questionnaire. Only assessed as frequency of tinnitus. **Other adverse effects** Narrative statement regarding a variety of adverse effects. It is not clear whether these were systematically assessed and reported. **Other outcomes reported in the study:** Aural fullness Duration of vertigo attacks Relapse in symptoms from week 18 to end of follow‐up Change in frequency of tinnitus Number of participants with severe disability and limited vertigo control	
Funding sources	None reported	
Declarations of interest	No declaration is made	
Notes	**Research integrity checklist:** No retractions/expression of concern were identified Trial registration is not applicable (published prior to 2010) Baseline characteristics of the 2 groups are not excessively similar No loss to follow‐up occurred and no reason is given for this The treatment appears to have a strong effect on vertigo, despite the small sample size Equal numbers were recruited to each group. Minimisation was used to maintain balance across group of known potential confounding characteristics.	
***Risk of bias***		
**Bias**	**Authors' judgement**	**Support for judgement**
Random sequence generation (selection bias)	Unclear risk	Quote: "Two groups (n = 8 per group) were assembled by the method of minimization to accomplish the balance between groups". Comment: although minimisation is a valid alternative to randomisation, there are no further details provided regarding the prognostic factors used for minimisation, any statistical software that was employed for the process etc. This process may have been conducted by the investigators themselves, and could therefore not be viewed as equivalent to randomisation.
Allocation concealment (selection bias)	Unclear risk	Comment: as above, no information is provided. If investigators were involved in the process of minimisation then group allocation may have been foreseeable.
Blinding of participants and personnel (performance bias) All outcomes	High risk	Comment: no placebo was used. Although this is described as a "double‐blind trial" no description of blinding is present, and no placebo was used.
Blinding of outcome assessment (detection bias) All outcomes	High risk	Comment: as above, participants would have been aware of their group allocation. Most outcomes are reported by (unblinded) participants, therefore detection bias is a risk.
Incomplete outcome data (attrition bias) All outcomes	Low risk	Comment: no patient withdrawals from the trial were reported.
Selective reporting (reporting bias)	High risk	Comment: no protocol is reported. Different vertigo outcomes are reported at different time points (attacks per day at 18 weeks, control of vertigo at > 12 months). Number of vertigo attacks per day is not reported at complete follow‐up. Hearing outcomes are not reported in a way that enables meta‐analysis.
Other bias	Unclear risk	Comment: the timing of follow‐up for those in the control arm is not clear, which may lead to a difference between the groups.

**Table:** Park 2016

***Study characteristics***		
Methods	Parallel‐group, open‐label RCT with 3 months duration of treatment and follow‐up	
Participants	**Setting:** Multicentre trial conducted at 9 sites in Korea. Study dates were not reported. **Sample size:** **Number randomised:** 220 participants **Number completed:** 187 participants **Participant baseline characteristics** **Age:** Reported for the entire study population: mean 52.39 years (SD 10.99) **Gender:** Reported for the entire study population: 91 (45%) male: 111 (55%) female **Probable/definite Ménière's disease:** All participants had definite disease (inclusion criterion) **Duration of disease:** Not reported **Attack frequency at baseline:** Isosorbide group: mean 5.14 attacks per 4 weeks (no SD reported) Control group: mean 4.88 attacks per 4 weeks (no SD reported) **Hearing loss at baseline:** Not reported **Measure of tinnitus at baseline:** Not reported **Number of participants with bilateral disease:** Not reported **Inclusion criteria:** Definite Ménière's disease, according to the** **AAO‐HNS 1995 criteria. A history of vertigo that persists for more than 20 minutes, with 2 or more episodes during the last 3 months. A medical record of hearing loss from a hearing test and tinnitus or aural fullness. Aged between 20 and < 80 years. **Exclusion criteria:** A history of intratympanic injection or surgical treatment within the last 6 months. Use of the study medication (betahistine or isosorbide) within the last 3 months as a treatment for Ménière's disease. **Diagnosis of Ménière's disease:** AAO‐HNS 1995 criteria for definite Ménière's disease	
Interventions	**Intervention (n = 110 randomised, n = 90 completed)** 90 mL isosorbide 3 times daily, after meals (total dose and concentration not stated). The dose could be reduced at the investigator's discretion. It is not reported whether this was necessary, and in how many participants. **Comparator (n = 110 randomised, n = 97 completed)** No intervention **Background interventions administered to all participants** All participants received betahistine tablets 6 mg 3 times daily (total daily dose 18 mg)	
Outcomes	**Primary outcomes relevant to this review:** **Improvement in vertigo** Not reported **Change in vertigo** Change in frequency of vertigo was reported as the number of episodes in a 4‐week period **Serious adverse events** Not apparently systematically assessed or reported; narrative summary of adverse events only **Secondary outcomes relevant to this review:** **Disease‐specific health‐related quality of life** Assessed with the Korean version of the DHI **Hearing** Assessed with pure tone audiometry at 4 frequencies **Tinnitus ** Assessed with the Korean THI **Other adverse effects** Not apparently systematically assessed or reported; narrative summary of adverse events only **Other outcomes reported in the study:** Speech discrimination score Electrocochleography Korean Functional Level Scale Change in frequency of vertigo at 4 weeks	
Funding sources	Not reported	
Declarations of interest	The authors declare that there are no potential conflicts of interest	
Notes	**Research integrity checklist:** No retractions of expressions of concern were identified No prospective trial registration was identified Limited baseline characteristics were available to compare the 2 groups Some loss to follow‐up was noted The study is free from implausible results Identical numbers of participants were randomised to each group, with no description of blocked randomisation	
***Risk of bias***		
**Bias**	**Authors' judgement**	**Support for judgement**
Random sequence generation (selection bias)	Unclear risk	Comment: no information on random sequence generation was provided.
Allocation concealment (selection bias)	Unclear risk	Comment: no information on allocation concealment was provided.
Blinding of participants and personnel (performance bias) All outcomes	High risk	Quote (translated): "We couldn’t conduct a double‐blind method using a placebo due to ethical issues and difficulties in making a placebo". Comment: open‐label study, no blinding.
Blinding of outcome assessment (detection bias) All outcomes	High risk	Quote (translated): "We couldn’t conduct a double‐blind method using a placebo due to ethical issues and difficulties in making a placebo". Comment: open‐label study, no blinding of outcome assessors.
Incomplete outcome data (attrition bias) All outcomes	Low risk	Comment: some differential loss to follow‐up (19% in intervention group, compared to 12% in control group) but unlikely to have made a considerable impact on the (continuous) outcome data.
Selective reporting (reporting bias)	Unclear risk	Comment: study protocol was registered with ClinicalTrials.gov, but we note that the registration took place retrospectively.
Other bias	Low risk	Comment: no other concerns identified.

**Table:** Ricci 1987

***Study characteristics***		
Methods	Randomised, placebo‐controlled, parallel‐group trial. The duration of the trial varied for each patient: each was treated for a period equivalent to 10 times the average interval between vertigo attacks before treatment (approximately 12 months of treatment and follow‐up).	
Participants	**Setting: ** Trial conducted in Italy. No further information provided. **Sample size:** **Number randomised:** 10 participants **Number completed:** 10 participants **Participant baseline characteristics** **Age: ** Betahistine group: mean 36.4 years (SD 2.2) Control group: mean 37.0 years (SD 5.4) **Gender: ** Betahistine group: 2 males: 3 females Control group: 4 males: 1 females **Probable/definite Ménière's disease: ** Not reported **Duration of disease:** Betahistine group: mean 39.2 months (SD 7.3)** ** Control group: mean 32.8 months (SD 10.0) **Attack frequency at baseline: ** Betahistine group: approximately once per month (mean interval between dizziness episodes was 26 days (SD 6)) Control group: approximately once per month (mean interval between dizziness episodes was 29 days (SD 6)) **Hearing loss at baseline:** Not reported **Measure of tinnitus at baseline: ** Not reported **Number of participants with bilateral disease:** Not reported **Inclusion criteria:** Study only states patients with Ménière's disease **Exclusion criteria:** Known sensitivity to the drug, peptic ulcer, gastroduodenitis, arterial hypertension, bronchial asthma, pheochromocytoma, severe asthenia and renal or hepatic insufficiency **Diagnosis of M**é**ni**è**re's disease:** Not reported	
Interventions	**Intervention (n = 5 randomised, n = 5 completed)** Betahistine; 16 drops (equal to 8 mg), 3 times per day after a meal. Taken for a period equivalent to 10 times the mean duration of the interval between attacks of vertigo reported prior to treatment. Mean duration of treatment 10.4 months (SD 1.2). **Comparator (n = 5 randomised, n = 5 completed)** Placebo; presumed to be identical to intervention group, but using placebo, although this is not clearly reported. Taken for a period equivalent to 10 times the mean duration of the interval between attacks of vertigo reported prior to treatment. Mean duration of treatment 7 months (SD 1.3). **Background interventions administered to all participants** None reported. Quote: "During the study, concomitant therapies with antivertigenic drugs acting on the cerebral circulation, antihistamines and histaminomimetics were avoided."	
Outcomes	**Primary outcomes relevant to this review:** **Improvement in vertigo** Reported with the AAOO 1972 class of vertigo and hearing control Class A 1 ‐ absence of dizzy episodes for a period of time equal to 10 times the average duration of the interval between the dizziness attacks declared before treatment 2 ‐ improvement of hearing function (≥ 30 dB at an average of 0.5 kHz, 1 kHz, 2 kHz) Class B 1 – like A1 2 – no changes in hearing function (≤ 15 dB at an average of 0.5 kHz, 1 kHz, 2 kHz) Class C 1 – like A1 2 – worsening of hearing function (≥ 15 dB at an average of 0.5 kHz, 1 kHz, 2 kHz) Class D Failure to control dizzy episodes Improvement = class A, B or C. However, note that this is equivalent to absence of dizzy episodes, and those who have improved (but still experience dizziness) will not be included. **Change in vertigo** Not reported **Serious adverse events** The methods state that adverse events will be assessed, but these are not reported **Secondary outcomes relevant to this review:** **Disease‐specific health‐related quality of life** Not reported **Hearing** Continuous data are not reported; hearing is only reported as improved (≥ 30 dB better than baseline), unchanged or worsened **Tinnitus ** Not assessed with a validated scale; separate data for both groups are not reported **Other adverse effects** The methods state that adverse events will be assessed, but these are not reported **Other outcomes reported in the study:** Aural fullness	
Funding sources	Not reported	
Declarations of interest	Not reported	
Notes	**Research integrity checklist:** No retractions or expressions of concern were identified No trial registration was identified, but this study was published in 1987 No loss to follow‐up was reported, but the sample size is very small (10 participants) No implausible results were reported Equal numbers of participants were allocated to each group, without mention of blocked randomisation	
***Risk of bias***		
**Bias**	**Authors' judgement**	**Support for judgement**
Random sequence generation (selection bias)	Unclear risk	Quote: "Patients were assigned to treatment on the basis of a randomization list with placebo and with betahistine hydrochloride..." Comment: there was reference to a 'randomisation list' but this does not describe the process in sufficient detail to permit judgement.
Allocation concealment (selection bias)	Unclear risk	Comment: no information regarding concealment of allocation.
Blinding of participants and personnel (performance bias) All outcomes	Unclear risk	Comment: there was no report of blinding. We can only assume that patients were blind to treatment allocation as a placebo was used. It is unclear whether study personnel would have been blinded to treatment allocation. The outcomes could be influenced by performance bias.
Blinding of outcome assessment (detection bias) All outcomes	Unclear risk	Comment: there was no report of blinding of outcome assessors. The outcomes could be influenced by detection bias.
Incomplete outcome data (attrition bias) All outcomes	Low risk	Comment: full follow‐up was reported.
Selective reporting (reporting bias)	High risk	Comment: no study protocol was available. Pre‐specified outcomes included biochemical test results and adverse events to assess drug tolerance. These were not reported.
Other bias	High risk	Comment: inclusion criteria were not reported, and no details were provided on diagnostic confirmation, which raises the possibility of selection bias. The small number of participants may have been insufficient to achieve a balance of confounding factors across the groups. Scoring system for vertigo may not be sufficient to capture changes appropriately. Any vertigo episodes were regarded as a failure of treatment (class D), therefore improvement is only rated as "cured" or "not cured", rather than "improved" or "not improved".

**Table:** Schmidt 1992

***Study characteristics***		
Methods	Placebo‐controlled, randomised, cross‐over trial. Full trial included 16 weeks of placebo and 16 weeks of betahistine treatment. For the purposes of this review we have included the first phase of the trial only.	
Participants	**Setting: ** Single‐centre hospital outpatient clinic in the Netherlands. Recruitment took place between 1984 and 1989. **Sample size:** **Number randomised:** 40 participants **Number completed:** 35 participants **Participant baseline characteristics** **Age: ** Betahistine group: 49.5 years (SD 10.1) Control group: 49.1 years (SD 7.5) **Gender: ** Betahistine group: 10 males (55.6%): 8 females (44.4%) Control group: 14 males (82.4%): 3 females (17.3%) **Probable/definite Ménière's disease: ** Not reported **Duration of disease: ** Betahistine group: 5.4 years (SD 5.6) Control group: 2.6 years (SD 1.7) **Attack frequency at baseline: ** Not reported **Hearing loss at baseline:** Average air conduction threshold at 0.25 kHz, 0.5 kHz, 1 kHz and 2 kHz Betahistine group: 58.8 dB HL (SD 20.4) Control group: 55.3 dB HL (SD 15.5) **Measure of tinnitus at baseline: ** Minimum masking level Betahistine group: 55 dB HL (SD 25) Control group: 55 dB HL (SD 26) **Number of participants with bilateral disease:** Betahistine group: 5/18 Control group: 3/17 **Inclusion criteria:** Patients who had experienced an exacerbation of Ménière's disease within the previous month, for which patients sought medical help. Unilateral or bilateral disease. **Exclusion criteria:** Infection of the middle or the inner ear. Peptic ulcer, bronchial asthma or pheochromocytoma. Pregnant. Liver or kidney insufficiency, brain tumour, recent head trauma, Parkinson's disease, epilepsy, multiple sclerosis or any other generalised disease. Operated upon because of Ménière's disease (destruction of labyrinth, saccus drainage, transection of vestibular nerve, etc.). Using antihistamines, anti‐vertiginous drugs, vasodilators, psychotropic drugs or tranquillisers, [if the] use of these drugs could not be stopped. Using betahistine dihydrochloride 3 times 16 mg daily or more for at least the previous 3 months. Experienced side effects during previous use of betahistine dihydrochloride. **Diagnosis of M**é**ni**è**re's disease:** Complete Ménière's disease was diagnosed according to the Utrecht working definition: Cochlear hearing loss History of tinnitus History of attacks of vertigo Exclusion of other diseases that could account for these symptoms To confirm the diagnosis and exclude other disease, patients were examined according to a fixed diagnostic protocol: Complete otological and vestibular history, as well as a general otorhinolaryngological, neurological, ophthalmological history ENT examination Hearing tests Measurement of tinnitus Vestibular tests Blood analysis CT of the temporal bones Neurological examination	
Interventions	**Intervention (n = presumed 20 randomised, n = 18 completed)** Betahistine; capsules containing 24 mg of betahistine dihydrochloride in a sustained‐release form, 1 capsule was taken every 8 hours for 16 weeks **Comparator (n = presumed 20 randomised, n = 17 completed)** Placebo; identical placebo was taken (composition not reported), 1 capsule every 8 hours for 16 weeks **Background interventions administered to all participants** None reported. The first week of the trial was used as a wash‐out period. From this week on, all other previously prescribed medication for Ménière's disease was stopped. A wash‐out period of 3 months was used in patients taking flunarizine, due to the long half‐life.	
Outcomes	**Primary outcomes relevant to this review:** **Improvement in vertigo** Not assessed **Change in vertigo** Assessed using an "imbalance scale" 'no attack': no turning sensation, no feelings of insecurity and no lightheadedness (0 points) 'mild attack': lasting a maximum of one minute, jerks, briefly dizzy and no nausea (1 point) 'moderate attack': lasting a maximum of five minutes, being dizzy, nauseous and having to lie down (4 points) 'severe attack': lasting hours, being nauseous with vomiting and having to go to bed (9 points) "The score for each type of attack was multiplied by the corresponding number of attacks. These values were added up to get the final score for 'imbalance'. In this way both frequency and intensity of vertigo attacks were amalgamated into a single score". A clinically relevant change was considered to be a change of 3 points (the difference between a mild and a moderate attack). Patients were provided with questionnaires to fill out at the end of each week to score their imbalance symptoms. **Serious adverse events** Article states "patients were asked to report all side effects" but no further information is reported which specifically assesses serious adverse events **Secondary outcomes relevant to this review:** **Disease‐specific health‐related quality of life** Not reported **Hearing** Hearing was measured every 4 weeks by pure tone audiometry (average air conduction threshold for the frequencies 0.25 kHz, 0.5 kHz, 1 kHz and 2 kHz). A change of more than 10 dB was considered clinically relevant. **Tinnitus ** Not assessed with a validated impact scale. Assessed with tinnitus loudness and minimal masking level, and self‐reported as none, mild, moderate or severe. **Other adverse effects** A number of specific adverse effects are reported, including headache, gastrointestinal disturbance and sleep disturbance **Other outcomes reported in the study:** Subjective hearing loss and change in hearing Subjective effect on pressure sensation Patient treatment preference Subjective impression of the effect of the trial Speech audiometry Tinnitus loudness, minimum masking level Caloric excitability, caloric directional preponderance and rotational preponderance	
Funding sources	Quote: "Publication of this thesis was supported by Entermed BV, Duphar Nederland BV, Astra Pharmaceutica BV, ARTU Biologicals NV, Glaxo BV, SmithKline Beecham Farma, Schering‐Plough BV, UCB Farma Nederland BV, Roussel BV, Bayer Nederland BV, the ORLU Foundation." Pharmaceutical company funding.	
Declarations of interest	No declarations are stated	
Notes	**Research integrity checklist:** No retractions or expressions of concern were identified The trial was not pre‐registered, but was conducted in the 1980s Limited information was available on baseline characteristics, but no concerns are present based on the data seen Dropouts were fully reported No implausible results are reported The number randomised to each group is not fully reported, but may have been equal (presumed 20 participants were initially randomised to each group)	
***Risk of bias***		
**Bias**	**Authors' judgement**	**Support for judgement**
Random sequence generation (selection bias)	Unclear risk	Quote: "The patients were assigned randomly to one of the two groups." Comment: no detail was provided regarding sequence generation.
Allocation concealment (selection bias)	Unclear risk	Quote: "The patients were assigned randomly to one of the two groups." Comment: no detail was provided regarding concealment of allocation.
Blinding of participants and personnel (performance bias) All outcomes	Unclear risk	Quote: "In a randomized, double‐blind cross‐over trial lasting 33 weeks, the effects of sustained‐release betahistine dihydrochloride (betahistine SR) on the subjective experience and the objective findings in patients with Menière's disease were compared with the effects of a placebo." Comment: the only mention of blinding in the Methods section is in the general description of the study design (as above). The use of a placebo can be taken as evidence that the patients were blind to the treatment received. However, it is unclear whether study personnel were also blinded.
Blinding of outcome assessment (detection bias) All outcomes	Low risk	Comment: the majority of outcomes are reported by (blinded) participants.
Incomplete outcome data (attrition bias) All outcomes	Low risk	Comment: small number of dropouts, balanced across groups. Inclusion of these data would be unlikely to substantially alter the conclusions of the trial.
Selective reporting (reporting bias)	High risk	Comment: no protocol was identified. Methods do suggest that some outcomes were recorded but not reported, e.g. "Frequency and quality of the tinnitus were not used to study the effect of the treatment in this trial". It is unclear why these outcomes were assessed if they were not to be used for analysis. Many results are presented as mean changes only, without a SD, precluding their inclusion in a meta‐analysis. It also appears that analysis methods had to be adapted during the trial due to problems with calculation of outcome measures: "In calculating the patients' scores several problems were encountered. They were handled in the following ways: If more than one compartment of the same item was filled in, the score in between the score of those two compartments was used. Partially filled in compartments were considered as filled in completely. 'Daily insecure feelings' was scored as seven points. If a patient was seen after five instead of four weeks, the questionnaires of the first, second, fourth and fifth week of that month were used, and the questionnaire of the third week was left out of the statistical analysis. In case of a three‐week period the questionnaire of the second week was used twice. When a patient with bilateral Meniere's disease filled in different compartments for each ear, the highest score corresponding with the worst ear was used for statistical analysis. In case one value was missing it was replaced by the average score of the preceding and the following value in calculating the average monthly score. lf two values were missing in one month, this month was regarded as missing."
Other bias	High risk	Comment: lack of a validated scoring system for assessing change in vertigo. Potential risk of detection bias.

AAO‐HNS: American Academy of Otolaryngology – Head and Neck Surgery BP: blood pressure BPPV: benign paroxysmal positional vertigo CI: confidence interval CT: computerised tomography DHI: Dizziness Handicap Inventory FLS: Functional Level Scale IT: intratympanic ITT: intention‐to‐treat MD: Ménière's disease MI: myocardial infarction MR: magnetic resonance PP: per protocol PPV: paroxysmal positional vertigo PTA: pure tone average RCT: randomised controlled trial SD: standard deviation THI: Tinnitus Handicap Inventory

### Characteristics of excluded studies [ordered by study ID]

**Table:**

Study	Reason for exclusion
Albernaz 1968	Intervention (dorperidol) is not relevant for this review
Albernaz 1970	This is not an RCT and only 12 of the participants included had Ménière's disease
Beckmann 1970	This is not an RCT: the study is a retrospective comparison of 2 treatment regimes
Beigh 2017	Duration of follow‐up too short (5 days only)
Beliakova 1971	This is not an RCT and the intervention is not appropriate for this review (sodium bicarbonate)
Bosch 1970	This is not an RCT
Brookes 1982	This is not an RCT
Burkin 1967	The duration of follow‐up for this trial was insufficient (< 3 months)
Canty 1981	Participants with Ménière's disease were excluded from this study
Celestino 1969	This is a review article, not an RCT
Celestino 1970	This is not an RCT, it is an observational study
Cohen 1972	This study included the wrong population: only 4 participants had Ménière's disease, the remainder had other causes of vertigo
Conde 1965	This is a review article on the treatment of vertigo, not an RCT
Devantier 2020	This is a meta‐analysis and no new data are included. The reference list of this article has been checked to ensure any relevant studies are included in this review.
Dimitriadis 2017	This is a meta‐analysis and no new data are included. The reference list for this article has been checked to ensure any relevant studies are included in this review.
Dowdy 1965	This is not an RCT
Duphar 108.005 80/M	Participants had vertigo, but no details to suggest that Ménière's disease was the cause in any/all of the participants
Duphar H. 108.5009/M	Participants in this study were required to suffer from vertigo, but the cause of their symptoms was not described. It is unclear whether all participants would have been diagnosed with Ménière's disease.
Duphar H 108.906 NL	Participants were diagnosed with a variety of causes of vertigo. Only 1 participant (out of 190) was diagnosed with Ménière's disease.
Duphar H 108 027 86 F/M	Insufficient details on the participants included ‐ no diagnostic criteria for Ménière's disease were used, and no information on how many participants had been diagnosed with Ménière's disease
Elia 1965	This trial had an insufficient duration of follow‐up (< 3 months)
Elia 1966	This study had an insufficient duration of follow‐up (< 3 months)
Elia 1970	This is not an RCT
Ernst 2017	This is not an RCT
Frew 1976	This is not an RCT
Godlowski 1965	This is a narrative review article and not an RCT
Greiner 1975	This is not an RCT ‐ all participants received betahistine
Guay 1970	This is not an RCT
Guyot 2008	The intervention in this study is not appropriate, as it involves intratympanic application of an antiviral agent, and not systemic administration
Hausler 1989	This is not an RCT
Helling 2017	This is a comment on Adrion 2016 by a different author: it does not contain any additional, relevant information
Hommes 1972	This is not an RCT
Huy 1992	The comparator is not appropriate in this study: participants were all allocated to betahistine either twice or thrice daily
James 2003	This is not an RCT, it is a systematic review. The reference list has been checked to ensure all relevant studies are included in this review.
James 2004	This is not an RCT, it is a systematic review. The reference list has been checked to ensure all relevant studies are included in this review.
Jongkees 1972	This is not an RCT
Klockhoff 1967	This is a cross‐over trial in which data cannot be extracted for the first phase of the study
Klonowski 1972	This is not an RCT
Lazeanu 1968	This is not an RCT
Liu 2020	This RCT had an insufficient duration of follow‐up (< 3 months)
Murdin 2016	This is a systematic review that considers the use of betahistine for symptoms of vertigo (not just Ménière's disease). The reference list has been checked to ensure that all relevant studies have been included in this review.
Najwer 1973	This is not an RCT
NCT01526408	Study terminated: this trial was terminated after enrollment of only 11 participants and no outcome data were assessed for any of the participants enrolled
Okamoto 1968	This study had an insufficient duration of follow‐up (< 3 months)
Oosterveld 1984	This is a cross‐over trial; data from the end of the first phase are not available for analysis
Pialoux 1981	This is not an RCT
Popiel 1975	This is not an RCT
Przymanowski 1966	This is not an RCT
Redon 2011	This study only included participants who had already undergone vestibular neurotomy for Ménière's disease
Reker 1983	This is a prospective case series and not an RCT
Richards 1971	This is a narrative review article and not an RCT
Salami 1984	This study had an insufficient duration of follow‐up (< 3 months)
Segers 1972	This is a case series and not an RCT
Solvay H. 1 08.035.92/F	This study included a mixed population of participants suffering from Ménière's disease and other causes of vertigo. Subgroup data are presented for those with Ménière's disease, but the duration of follow‐up was only 30 days, therefore was insufficient for this review.
van Deelen 1986	This is a cross‐over study and data from the first phase of the study cannot be extracted
Van Esch 2022	This is a systematic review with no new relevant data. The reference list has been checked to ensure that any relevant studies have been included in this review.
Watanabe 1967	This is a cross‐over trial and data from the end of the first phase are not available. The duration of follow‐up was also insufficient (< 3 months).
Wilmot 1976	This is a cross‐over trial; data are not available for analysis at the end of the first phase of the trial
Wolfson 1967	This is not an RCT
Wouters 1983	This is not an RCT
Yamazaki 1988	The comparator for this study is not appropriate: the diuretic isosorbide is compared to betahistine, with not group receiving either placebo or no treatment
Yu 2012	This RCT had an insufficient duration of follow‐up (< 3 months)

RCT: randomised controlled trial

### Characteristics of ongoing studies [ordered by study ID]

**Table:** ACTRN12612000053820

Study name	A phase II, double‐blind, placebo‐controlled clinical trial of long‐term (up to 9 months) daily treatment with two 500 mg Valtrex (valaciclovir) tablets for the chronic suppression of recurrences of Ménière's disease symptoms in participants with a positive herpes serology
Methods	Parallel‐group randomised controlled trial with 9 months duration of treatment and an additional 3 months duration of follow‐up
Participants	Participants diagnosed with Ménière's disease (according to the AAO‐HNS 1995 criteria) with a positive herpes serology Additional inclusion criteria: Positive transtympanic electrocochleography Clinical history of labial herpes infection Clinical history of cluster of attacks with periods of remission Aged 18 to 60 years Target sample size of 80 participants
Interventions	1 g valaciclovir daily for 9 months, compared to placebo
Outcomes	Frequency of vertigo attacks (assessed with a daily diary with a rating scale of 0 to 5 reflecting the severity of an attack) Subjective hearing function (assessed with a daily diary) Subjective level of tinnitus (assessed with a daily diary) Subjective level of wellbeing (assessed with a daily diary, using a rating scale of 0 to 5) Time to first vertigo attack
Starting date	Anticipated start date was 1 April 2012, however the trial registry site still states "not yet recruiting"
Contact information	No details are provided
Notes	We have been unable to find any details regarding whether this trial is ongoing or was prematurely terminated

**Table:** NCT01729767

Study name	Efficacy of antiviral medications in controlling vertigo attacks of patients with Meniere's disease
Methods	Parallel‐group randomised controlled trial with approximately 50 days of treatment and further 40 days of follow‐up
Participants	Participants with Ménière's disease Aged 18 to 85 years With at least 2 episodes of vertigo per month, severely interfering with function and lasting for at least 20 minutes Not on any medication for the preceding 3 months Target sample size of 80 participants
Interventions	Aciclovir 400 mg 5 times daily for 1 month, then 3 times daily for 10 days, then twice daily for 10 days, compared to placebo tablets All participants will be encouraged to take a low‐salt diet
Outcomes	Vertigo Hearing loss Aural fullness Tinnitus No further details on the methods of outcome assessment
Starting date	August 2011. Study was due to have completed by December 2012.
Contact information	Masoud Motesadi motesadi@sina.tums.ac.ir Parisa Mazaheri mazaheri.parisa@gmail.com
Notes	We have attempted to contact the trial contacts to ascertain whether this study is ongoing, or was ever published, as we have been unable to locate any publications that relate to it. We are awaiting a response.

## Differences between protocol and review

Due to the paucity of data for most outcomes, we were unable to carry out a number of pre‐planned sensitivity analyses and subgroup analyses. However, we added one unplanned sensitivity analysis in this review. As described in Sensitivity analysis, we assessed whether changing the planned outcome "improvement in vertigo" to "complete resolution or substantial improvement in vertigo" would alter the effect estimates. As this was a post hoc change, the results should be interpreted with caution. However, we considered that people with Ménière's disease may want to know if an intervention had a marked effect on their vertigo symptoms, rather than a more modest change. 

We planned to use the Trustworthiness Screening Tool from Cochrane Pregnancy and Childbirth to identify studies for inclusion in the main analysis. However, as described in Selection of studies, we did not exclude studies from the main analysis on the basis of concerns whilst using this tool. We considered that the overall certainty of the review findings (all very low‐ or low‐certainty) would not be impacted by this decision. 

As planned in our protocol, if the AAO‐HNS 1995 criteria had not been used to diagnose Ménière's disease, we considered whether to include the study. We took the post hoc decision that if a study had been conducted in an ENT department then it was reasonably likely that efforts had been made to exclude other possible diagnoses. This decision was taken by authors who were blinded to the other features and results of the studies. However, we acknowledge that this may result in a more heterogeneous population. We therefore reflected this by lowering the certainty of the evidence from studies with this limitation. 
